# Macrocycle Conformational Flexibility as a Key to Enantioselective Recognition

**DOI:** 10.1021/jacsau.6c00640

**Published:** 2026-07-13

**Authors:** Tadeu Luiz Gomes Cabral, Matthias Stein, Claudio F. Tormena

**Affiliations:** † Molecular Simulations and Design Group, 28307 Max Planck Institute for Dynamics of Complex Technical Systems, Sandtorstrasse 1, Magdeburg 39106, Germany; ‡ Chemistry Institute, University of Campinas - UNICAMP, PO Box 6154, 13083-970 Campinas, São Paulo, Brazil

**Keywords:** diffusion NMR, chiral recognition, receptor flexibility, macrocycle, DFT, conformational sampling

## Abstract

The differentiation and assignment of absolute configurations of enantiomers in mixtures remain a significant challenge for both experimental and computational methods. In recent years, diffusion-NMR (DOSY) experiments using chiral resolving agents have emerged as a promising approach for enantiodiscrimination. While many resolving agents demonstrate strong enantioselective capabilities, limitations remain in reliably assigning absolute configurations and elucidating the molecular basis of chiral recognition. In this study, we combine NMR experiments and computational approaches to investigate the differentiation of mandelic Acid (MA), α-methylbenzylamine, methyl mandelate, Mosher’s acid, 2-butanol, and camphor enantiomers by a chiral macrocycle (MAC) assembled from N,N′-bis­(6-acylamino-2-pyridinyl)­isophthalamide units and BINOL derivatives. NMR measurements reveal a stereopreference of MAC for the (*S*)-enantiomer, as evidenced by its lower diffusion coefficient compared to the (*R*)-enantiomer. Our computational modeling indicates that this enantioselectivity arises when the MAC adopts a closed conformation, rather than the expected open form. Temperature-dependent ^1^H and selective ROESY measurements further support an equilibrium between open and closed MAC conformers in solution, as also suggested by extensive conformational searches. Our combined experimental and computational approach demonstrates that only the closed MAC-enantiomer complexation simultaneously accounts for the observed diffusion-NMR and shielding differences and can rationalize the observed enantiodifferentiation. The results show that the macrocyclic receptor exhibits conformational flexibility that must be considered in chiral recognition events. Therefore, these insights advance our understanding of enantioselective interactions and provide a framework for the future design of chiral resolving agents for differentiation by diffusion-NMR and computational methods.

## Graphical abstract

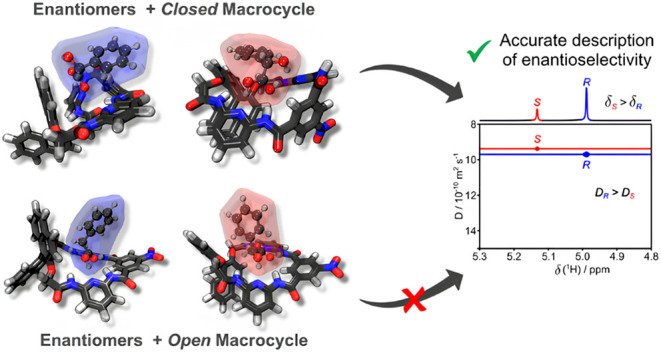

## Introduction

Chiral molecules are important building blocks in various fields, including pharmaceuticals, agriculture, materials science, and food chemistry, due to the potential risks posed by counter-enantiomers.^1–4^ The demand for enantiopure chiral compounds and the attempts at enantioselective synthesis continue at an unprecedented rate.^5,6^ In parallel, regulatory agencies, such as the U.S. Food and Drug Administration and the European Medicines Agency, require that each enantiomer of a drug candidate be individually characterized and that its physicochemical and biological properties be rigorously evaluated before approval for therapeutic use.^7,8^ The combination of stricter regulatory demands and the accelerating production of new compounds with stereocenters has intensified the need for fast and reliable stereochemical analysis. This is particularly important for the rapid characterization of both enantiomers in liquid mixtures at room or body temperatures.^9,10^

A variety of analytical techniques are available for enantiodiscriminative analysis,^11^ including chiral chromatography,^12^ circular dichroism spectroscopy,^13^ and X-ray diffraction.^14^ Although powerful, these methods often require labor-intensive, time-consuming preparative steps, such as prior physical separation, covalent derivatization, stereoselective crystallization, or the use of enantioenriched calibration standards to assign properties to both stereoisomers.^5,15,16^ Under these conditions, rapid and broadly applicable methods capable of distinguishing enantiomers in a mixture, determining the enantiomeric excess (*ee*), and assigning their absolute configuration remain a major challenge in both industrial and academic applications due to their laborious and time-consuming experimental work.^11,17,18^ In this context, nuclear magnetic resonance (NMR) spectroscopy has emerged as a powerful and versatile alternative for analyzing chiral systems. In a conventional NMR experiment, a mixture of enantiomers in an achiral medium resonates at identical chemical shifts (or frequencies) for both stereoisomers, producing overlapping signals.^17,19–22^ The introduction of a chiral resolving agent (CRA) overcomes this limitation by forming two diastereomeric complexes that differ in binding thermodynamics and lifetime, leading to distinct chemical shifts and enabling the direct spectral separation of the stereoisomers.^23,24^ Several resolving agents, including (*R*,*S*)-BINOL and derivatives, chiral metal complexes, and macrocyclic compounds, have been explored for this purpose.^20,25,26^ This strategy has proven to be successful for enantioselective discrimination and for determining the *ee* without previous physical separation. However, assigning absolute configurations to these systems remains challenging and is an ongoing area of research.^17^

Recently, our group demonstrated that combining chiral resolving agents with diffusion-NMR (Diffusion-Ordered Spectroscopy—DOSY), specifically through the chiral Matrix-Assisted DOSY (MAD) strategy, enhances the stereoanalytical capabilities of NMR.^17,19,20^ The MAD approach employs an additional matrix—typically an added compound or supramolecular assembly—to enhance differences in chemical shifts and diffusion coefficients (*D*) between analytes in a mixture.^27,28^ This strategy is not limited to chiral systems but is broadly applicable, enabling virtual separation of diverse organic compounds and alcohol isomers using micelles or functionalized nanoparticles as matrices.^29,30^ Beyond resolving enantiomers solely in the frequency dimension, matrix-assisted DOSY also provides differences in diffusion coefficients (*ΔD*), offering detailed mechanistic insights into chiral recognition and allowing the assignment of absolute configurations, a persistent challenge in solution-state NMR.^31,32^ As diffusion encodes information about molecular size, intermolecular interaction strength, and complex stability, matrix selection is crucial for achieving detectable diffusional separation.^27,28^ This stereodifferentiation was first demonstrated by Salomé and Tormena^20^ and later expanded by Cabral et al.,^17^ who showed that combining BINOL with chiral micelles (e.g., larger chiral matrices) increases the diffusion differences and enables assignment of absolute configuration. Furthermore, recent combined computational and experimental studies have clarified the molecular origins of stereoselectivity in MAD, revealing how specific binding modes and stereoselective complex-ligand interactions govern the observed NMR chiral recognition.^19^

Despite recent progress, some key aspects of chiral agents remain poorly understood. Most studies have focused on small and rigid CRAs (such as BINOL), whereas larger and more flexible agents with multiple torsional degrees of freedom and diverse conformational landscapes are still insufficiently characterized. For example, the (*R*)-chiral macrocycle Chirabite-AR (MAC, Figure 1) and its analogues are widely employed in asymmetric organocatalysis, chiral separations, and as chiral sensors.^33–37^ Although its synthesis and applications as a recognition agent for a range of analytes have been reported, only chemical shift differences (leading to the unambiguous assignment of absolute configurations) and titration experiments, assuming a single receptor conformation, were performed.^26,37^

**Figure 1:**
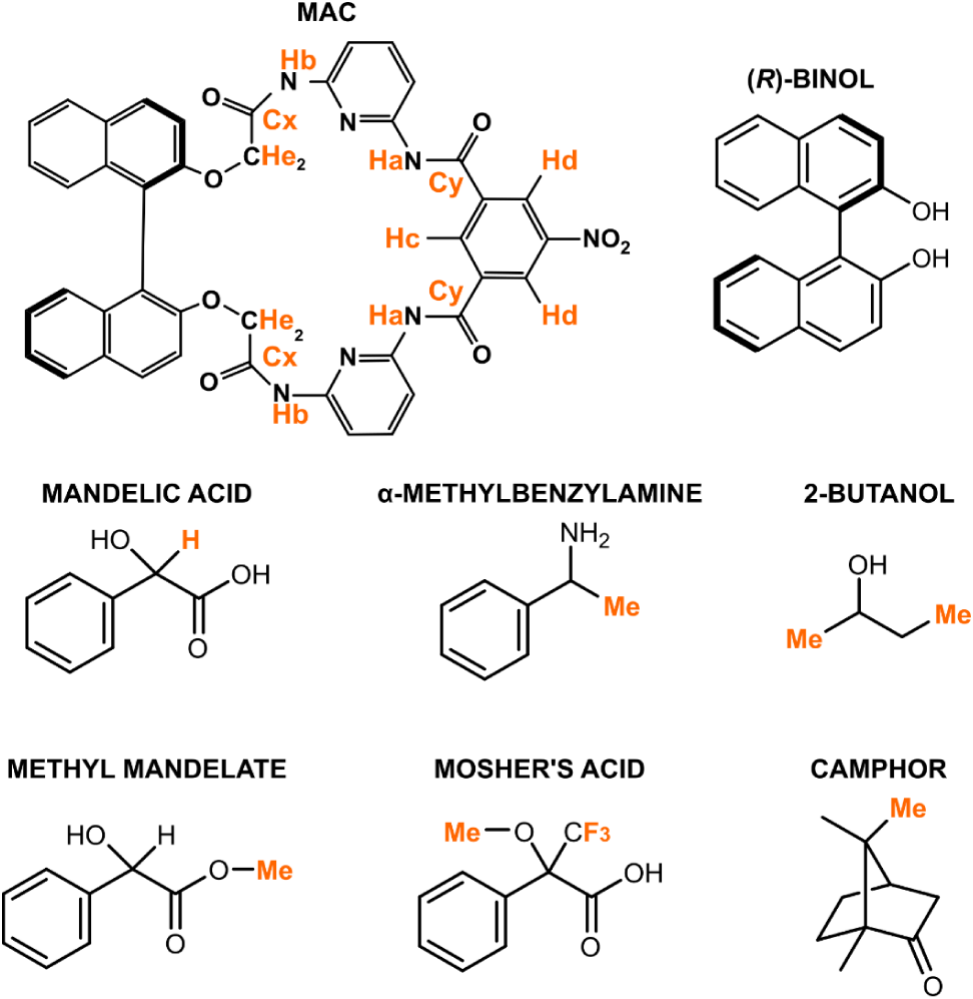
Structure of the chiral macrocycle CHIRABITE-AR (MAC), used here as a chiral resolving agent for the differentiation of various enantiomers (mandelic acid, α-methylbenzylamine, 2-butanol, methyl mandelate, Mosher’s acid, and camphor), together with the commonly employed chiral solvating agent (*R*)-BINOL. The highlighted hydrogen, methyl, and trifluoromethyl groups indicate the monitored atoms in this study.

Here, we investigate the enantiorecognition of six chiral compounds with distinct structural and functional characteristics (Figure 1). Among them, (*R*,*S*)-mandelic acid (MA) enantiomers were selected as the primary model system for discussion, as it is a widely used chiral building block in pharmaceutical and cosmetics formulations^38^ and has been a well-established model for host–guest stereodifferentiation studies.^17,19,39^ In practice, its racemates are thoroughly investigated and typically resolved by liquid chromatography,^40^ selective crystallization,^39,41^ or enzymatic synthesis^42^ In NMR study of matrix-assisted DOSY for the mandelic acid enantiodiscrimination, the larger MAC outperformed BINOL in terms of chemical shift differences (Δδ). In the diffusional differences (Δ*D*), however, the MAC only marginally improved compared to the smaller agents.^20^ Previous work could not rationalize the stereoselective trends, and the mechanistic origin of its improved frequency discrimination and its modest diffusion differentiation remained unclear.^20^ By using different resolving agents, it became obvious that differences in their molecular weight do not directly correlate with discrimination in the diffusional regime as expected.

Therefore, herein, we show that macrocycle conformational flexibility plays a decisive role in diffusion enantiodifferentiation and gives its mechanistic origin. By integrating diffusion NMR experiments, ^1^H and selective ROESY variable-temperature measurements with conformational sampling, and DFT calculations, we demonstrate that the conformational equilibrium of MAC must be considered and is key to rationalizing the observed stereoselectivity. This work thus provides fundamental insight into the stereoselective recognition of large and flexible macrocyclic receptors, offering a representative system for rigorous mechanistic analysis without evaluating a broad range of stereoisomers, which is beyond the scope of this work.^19^ We are pushing advanced experimental and computational strategies to their resolution limits in order to assign structural and thermodynamic factors governing chiral differentiation in both NMR frequency and diffusion dimensions simultaneously. Computed ^1^H and ^19^F chemical shifts and binding free energies agree well with experiments and accurately reproduce the differentiation patterns. Conformational sampling and quantum chemical refinement show that the free receptor can exist in an equilibrium between *open* and *closed* states, which is then confirmed by temperature-dependent NMR. Only the diastereomeric complexation with the *closed* form can explain the differences between homochiral and heterochiral complexes. Our findings shed light on the origin of this unexpected matrix-assisted DOSY behavior, provide a molecular-level picture of its stereoselectivity, and offer design principles for next-generation chiral matrices for absolute configuration assignments by diffusion NMR.

## Results and Discussion

The chosen macrocycle can differentiate between the (*R*,*S*)-MA enantiomers; however, the stereoselectivity has not been fully resolved so far, and no direct evidence of the chiral recognition process was obtained because of the lack of structural assignments.^20^ NMR experiments with an enantiomeric excess of the (*R*)-enantiomer were performed to identify and analyze stereoselective recognition patterns, thereby allowing the assignment and detailed analysis of the stereodiscrimination of the mandelic acid enantiomers and the other analytes examined by the chiral macrocycle.

As shown in Figure 2A, in the absence of MAC, the enantiomeric mixture of free (*R,S*)-MA exhibits superimposed ^1^H signals, preventing the assignment of stereochemistry. Upon addition of the macrocycle, both homochiral and heterochiral diastereomeric complexes are formed, resulting in a distinct chemical environment for each MA stereoisomer and thereby breaking the signals’ degeneracy, as illustrated in Figure 2B. In the presence of the chiral macrocycle, the mixture exhibits a signal separation (Δ*δ*_*RS*_ = *δ*_*R*_ – *δ*_*S*_) of −0.143 ppm (−84.1 Hz), with the (*R*)-MA enantiomer showing a greater shielding effect than the (*S*)-MA (*δ*_*R*_ < *δ*_*S*_). Interestingly, the same frequency separation trend (see Table 1 for details) was also observed for 2-butanol, camphor, α-methylbenzylamine, and Mosher’s acid (^19^F). In contrast, the ^1^H resonances of Mosher’s acid exhibited the opposite pattern behavior, with the (*S*)-enantiomer being more shielded than the (*R*)-enantiomer (*δ*_*S*_ < *δ*_*R*_, and Δ*δ*_*RS*_ displaying a positive value). Among all the analytes investigated, only methyl mandelate did not exhibit measurable signal separations under the experimental conditions, and no stereoselective discrimination could be observed.

**Figure 2:**
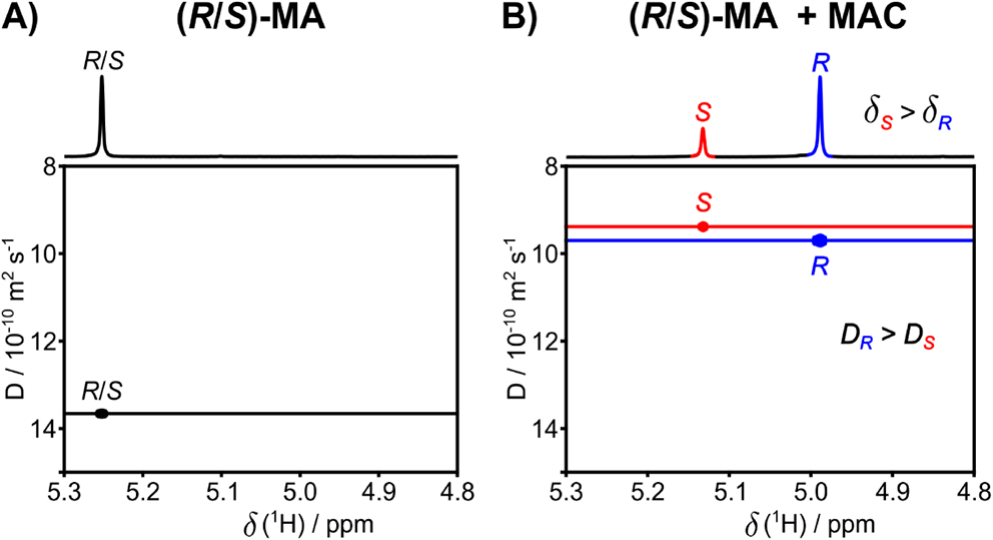
499.87 MHz ^1^H-DOSY with the least attenuated 1D spectrum displayed at the top for an A) enantiomeric mixture of 70% (*R*)-Mandelic Acid and 30% (*S*)-Mandelic Acid (MA) in deuterated chloroform, B) the same mixture in the presence of the chiral macrocycle (MAC).

**Table 1:** Experimental ^1^H and ^19^F Chemical Shifts Differences (Δδ*_RS_* = *δ_R_* *– δ_S_*, in ppm) and Diffusion Coefficients Separations (Δ*D_RS_* = *D_R_* – *D_S_*, in X 10^–10^ m^2^ s^–1^) for (*R,S*)-Analyte Enantiomers in the Presence of Chiral Macrocycle (MAC)^a^

Analyte	Δδ_*RS*_	Δ*D*_*RS*_ ± error
(R,S)-Mandelic acid	–0.143	+0.27± 0.07
(R,S)-Mosher’s Acid (^19^F)	–0.111	+0.17 ± 0.10
(R,S)-Mosher’s Acid (^1^H)	+0.068	+0.16 ± 0.03
(R,S)-α-methylbenzylamine	–0.004	+0.16 ± 0.06
(R,S)-Camphor	–0.003	+0.60 ± 0.09
(R,S)-2-butanol	–0.003	+0.80 ± 0.31
(R,S)-Methyl Mandelate	n.r.	n.r.

a Further information can be found in Supporting Information, Table S1.

For the analytes for which a measurable chemical shift difference was observed, the diffusion coefficients could also be resolved for each stereoisomer. When the chiral macrocycle is added to the mixture, the diffusion coefficients of both enantiomers decrease relative to those of free stereoisomers due to complex formation. As already seen for mandelic acid (Figure 2), all investigated analytes exhibited slower diffusion for the (*S*)-enantiomer–(*R*)-MAC complex than for the corresponding (*R*)-enantiomer–(*R*)-MAC complex (*D* ^(*S*)‑*enantiomer*^ < *D* ^(*R*)‑*enantiomer*^); see Table 1 and Supporting Information - Section 3.2 for details), suggesting the heterochiral complex to be more stable and longer-living than the homochiral species.

Under fast exchange conditions on the diffusion time scale, the experimentally observed diffusion coefficient (*D*_*obs*_) corresponds to a population-weighted average of the diffusion coefficients of free (*D*_*free*_) and complexed (*D*_*complex*_) species:

$$D_{obs}=f_{free}D_{free}+f_{complex}D_{complex}$$

where *f*_*free*_ and *f*_*complex*_ represent the mole fractions of free and complexed analyte, respectively.^17,27,28^ Stronger binding increases the population and the residence time of the analyte in the complexed state, leading to a lower observed diffusion coefficient. While this approach does not directly provide experimental values of the binding free energy (Δ*G*_*bind*_), differences in diffusion coefficients (Δ*D*) qualitatively reflect differences in the effective stability and lifetime of the complexes and, therefore, capture the relative thermodynamic information about the chiral binding equilibrium.^17,19,28^

The diffusion-NMR results demonstrate that this chiral macrocycle outperforms commonly employed resolving agents, such as BINOL, in the chiral discrimination of (*R*,*S*)-MA.^17,19,20^ For diffusion coefficient discrimination, this improvement is expected since MAC has a larger molecular weight and hydrodynamic radius than BINOL. The more substantial perturbation of analyte mobility leads to slightly higher diffusion coefficient differences.^17^ However, the diffusional separation (Δ*D*_*RS*_ = *D*_*R*_ – *D*_*S*_ = 0.27 ± 0.07 × 10^–10^ m^2^ s^–1^) is only around 3.8 times greater than for (*R*)-BINOL.^19^ In contrast, the separation in chemical shifts (Δ*δ*) is approximately 24 times larger than for BINOL, demonstrating a substantially enhanced ability to resolve MA stereoisomers in the frequency dimension. In other words, the macrocycle enhances chemical shift discrimination by over an order of magnitude relative to the smaller resolving agent. However, in the diffusion dimension, the separations Δ*D* are only a few times larger than those observed when BINOL is employed. The discrepancy between strong chemical shift discrimination and only modestly enhanced differences in the diffusion regime appears puzzling and counterintuitive. Comprehensive computational investigations were performed to rationalize the observed enantiodiscrimination, elucidate the mechanism of chiral recognition, and identify the structural and thermodynamic factors governing trends in chiral discrimination during the formation of the enantiomer-MAC diastereomeric complexes.

Molecular dynamics (MD) simulations and density functional theory (DFT) calculations have previously shown that differences in the diffusion coefficients of enantiomers correlate well with differences in Gibbs free energies of binding for homochiral vs heterochiral complexes.^19^ In general, a more negative Gibbs free energy of binding is associated with enhanced complex stability, resulting in a lower diffusion coefficient for the respective enantiomer-MAC complex.^19^ An accurate computational description of the relative binding energies of these diastereomeric complexes is essential to rationalizing the chiral recognition responsible for NMR enantioseparation. Moreover, reliable computational modeling must simultaneously reproduce the experimental trends in the chemical shift differences. Thus, the thermodynamics of binding and chemical shift differences must both agree with experimental data to draw reliable statements and conclusions.

To address this, we used a computational workflow to obtain binding free energies and chemical shifts, involving semiempirical docking and CREST (Conformer Rotamer Ensemble Search Tool) in combination with CENSO (Commandline ENergetic SOrting) to generate, conformationally sample, and refine each diastereomeric complex in an unbiased and consistent manner (as described in Supporting Information, section 2).^43–47^ As a starting point, docking calculations were performed using a macrocycle geometry analogous to available crystal structures reported for related systems in which chiral recognition was also investigated.^26,34,37^ This starting macrocycle geometry is here termed the *open* macrocycle conformation. Following docking, the resulting lowest-energy homochiral and heterochiral complexes were then subjected to conformational sampling using a combination of classical metadynamics (MTD)/MD simulations to give tens to hundreds of unique structures, which were subsequently refined at various DFT levels with increasing accuracy (see Supporting Information, Table S15).

After extensive sampling and QM refinement, the homochiral (MAC + (*R*)-MA) ensemble converged to two unique low-energy structures, while the heterochiral (MAC + (*S*)-MA) ensemble resulted in three distinct complex geometries within a narrow energy window (see Section 4.1 of the Supporting Information for the number of unique structures for the other analytes). Figure 3A and B show the lowest-energy homochiral and heterochiral complexes between mandelic acid stereoisomers and the *open* macrocycle conformation after the full DFT refinement. In both diastereomeric complexes, mandelic acid enantiomers associate through hydrogen bonds between their hydroxyl/carboxyl groups and the amide groups of the chiral macrocycle. The computed chemical shifts qualitatively reproduce the experimental trend, in which (*R*)-MA is more shielded than (*S*)-MA (see Table 2). On the other hand, the calculated binding free energy differences fail to even qualitatively match the thermodynamic trends expected from the observed diffusion differences *D* ^(*S*)‑*MA*^ < *D* ^(*R*)‑*MA*^; therefore, $\Delta G_{bind}^{MAC+\left(S\right)-MA}<\Delta G_{bind}^{MAC+\left(R\right)-MA}$ , see Table 1 and Table 2). Experimentally, the heterochiral complex diffuses more slowly and, therefore, ought to be longer-living and thermodynamically favored. However, the calculated binding free energies for this *open* conformation give the opposite ordering. A similar inconsistency between binding free energy and the experimental diffusional stereoselectivity was also observed for the complexes between the *open* MAC and 2-butanol, camphor, α-methylbenzylamine, and Mosher’s acid (Tables 1 and 2). Apparently, (*R*,*S*)-enantiomer binding to the *open* form does not rationalize the chiral selective recognition and simultaneously reproduce the observed diffusion and chemical shift difference trends. Such a discrepancy is striking and was not considered in prior literature, in which it was assumed that the *open* form is responsible for the chiral recognition.^26,34,37^

**Figure 3:**
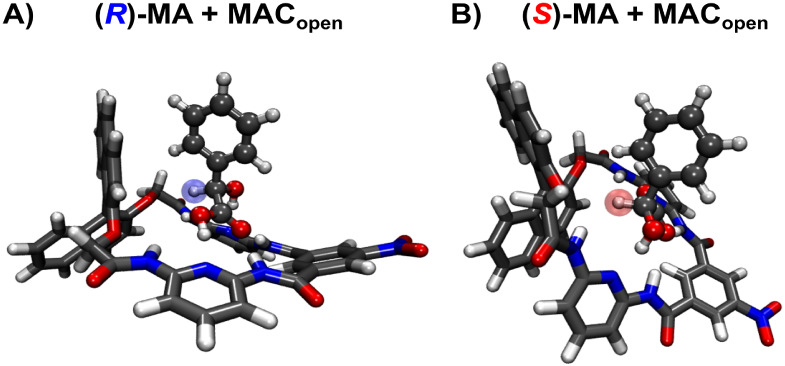
Lowest-energy structures of the complexes formed between the chiral macrocycle (*open* conformation—MAC*_open_*) and A) (*R*)-Mandelic Acid, B) (*S*)-Mandelic Acid (MA). Highlighted atoms represent the hydrogens at the chiral center of each MA enantiomer.

**Table 2:** Computed ^1^H and ^19^F Chemical Shifts Separations (Δ*δ_RS_* = *δ_R_* *– δ_S_*, in ppm) and Calculated Gibbs Free Energies of Binding Differences (ΔΔ*G_RS_* = Δ*G_R_* – Δ*G_S_*, in kcal.mol^–1^) for Enantiomers in Complex with *Closed* and *Open* Conformations of the Chiral Macrocycle (MAC)^a^ ^b^

(*R*)-MAC Conformation	Complex	Δδ_*RS*_	ΔΔG_*RS*_
*Closed*	(R,S)-Mandelic acid	–0.603	+5.700
	(R,S)-Mosher’s Acid (^19^F)	–0.213	+0.845
	(R,S)-Mosher’s Acid (^1^H)	+0.293	
	(R,S)-α-methylbenzylamine	–0.039	+1.328
	(R,S)-Camphor	–0.501	+0.273
	(R,S)-2-butanol	–0.286	+0.177
*Open*	(R,S)-Mandelic acid	–0.479	–1.600
	(R,S)-Mosher’s Acid (^19^F)	+4.700	–0.377
	(R,S)-Mosher’s Acid (^1^H)	–0.044	
	(R,S)-α-methylbenzylamine	0.179	–0.662
	(R,S)-Camphor	0.043	–0.289
	(R,S)-2-butanol	–0.109	–0.736

a Absolute values can be found in Supporting Information, Tables S16 and S17.

b The experimental diffusion pattern (*D* ^*heterochiral*^ < *D* ^*homochiral*^) implies $\Delta G_{bind}^{heterochiral}<\Delta G_{bind}^{homochiral}$ , thus, to reproduce the experimental trend ΔΔ*G*_*RS*_ must assume positive values.

This inconsistency strongly suggests the prevalence of an alternative, so far not considered, binding mode, which may be responsible for the stereoselective binding of analyte enantiomers to the receptor in solution. The prevalence of thermodynamic equilibria between different receptor conformations are observed for some macrocyclic structures.^48–52^ For example, the 24-atom triazine macrocycle has been reported to exist in both *open* and *closed* conformations, which exhibit different pH sensitivities and protonation states depending on the adopted conformation.^49^ Similarly, the chiral 24-atom glycine macrocycle can adopt either an extended or a folded structure, leading to distinct properties.^51^ None of these examples, however, refer to an equilibrium of MAC conformations in solution with respect to enantioselective recognition, as detected by the diffusion NMR studies presented here. Additionally, no alternative MAC conformation has been proposed or demonstrated to account for its use as a stereoselective agent.

In the quest for alternative receptor conformations and analyte enantiomeric binding modes, first conformational sampling of the isolated macrocycle in an implicit solvent was performed. The conformational sampling of the free MAC yielded an additional receptor conformation, which we refer to as the *closed* conformation of the macrocycle. This structure was not sampled previously since we initiated the search from the docked enantiomer-MAC complexes, in which the latter was in the o*pen* form. This hindered a full exploration of the receptor’s intrinsic conformational degrees of freedom. At the semiempirical level (GFN2-xTB), this *closed* macrocycle is 4.0 kcal mol^–1^ more stable than the *open* form. However, more accurate DFT calculations give a smaller or close-to-zero energy difference. For example, at the ωB97X-V/def2-TZVPP level, the *open* and *closed* conformers are within 0.1 kcal mol^–1^ of difference (see Supporting Information, Table S60). Thus, the *closed* and *open* conformations are expected to coexist in solution and can participate in complex formation with the analyte.

Given that both *open* and *closed* MAC states are predicted to be in equilibrium, we also sought experimental verification through variable-temperature ^1^H NMR and selective 1D ^1^H ROESY measurements of the free resolving agent to confirm the presence and relevance of the *closed* macrocycle conformation in solution. The ^1^H NMR spectra of the pure chiral macrocycle at different temperatures (Figure 4A) revealed a pronounced temperature-dependent shielding effect of proton Hd (see Figure 1), whereas the remaining aromatic signals exhibited only a minor variation. Such a temperature-dependent selective shielding suggests that lowering the temperature might create a chemical environment with a higher local electronic density around Hd, thereby enhancing its chemical shielding. Since Hd in the *open* conformation is relatively distant from electron-rich regions, this trend implies that at lower temperatures, the equilibrium tends to favor a more compact form (i.e., *closed* macrocycle arrangement), bringing Hd closer to the other chemical groups of the chiral macrocycle and thus experiencing a higher shielding effect. The selective 1D ^1^H-ROESY measurements (Figure 4B) provide direct experimental support for this hypothesis. The excitation of proton He (see Figure 1) yielded a weak but reproducible ROESY relation (inverted signals) with Hd across all of the investigated temperatures (Figure 4B). Because ROESY signals require spatial proximity, this observation further indicates that He and Hd must be close in space, an arrangement feasible only if the macrocycle adopts a *closed*, compact conformation rather than an extended, *open* form. In this scenario, the Hd can be found near aromatic groups and electron-rich regions of the chiral macrocycle, which plausibly accounts for the observed shielding effect.

**Figure 4:**
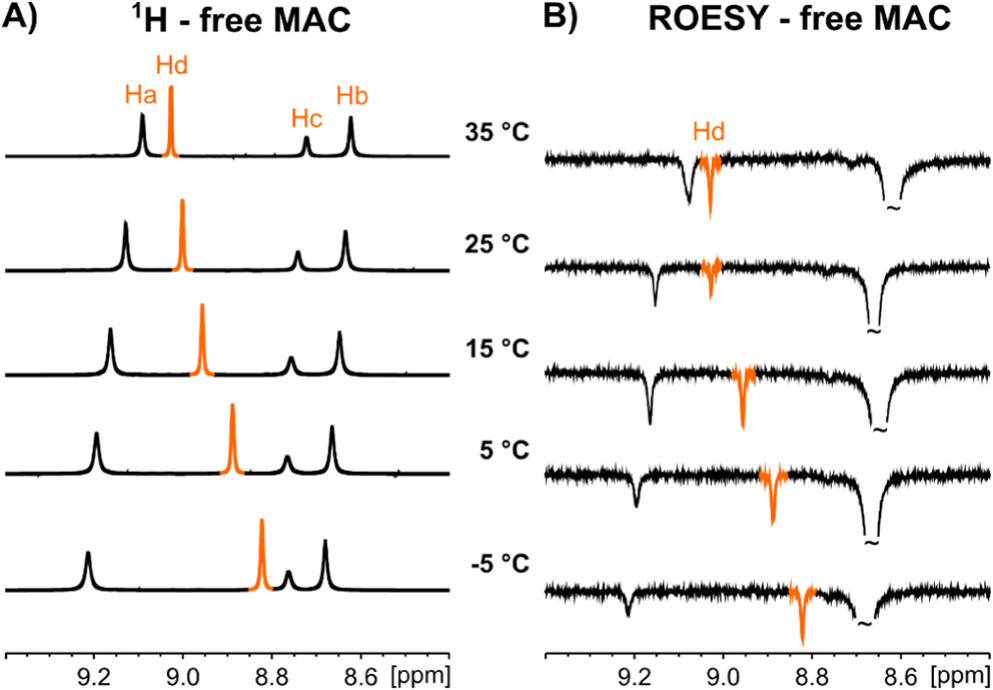
Selected spectral regions of the isolated chiral macrocycle (MAC) from 600.17 MHz A) ^1^H NMR, B) selective 1D ^1^H-ROESY spectra, both recorded at variable temperatures (−5 to 35 °C). In the ROESY spectra, inverted signals indicate through-space intramolecular interactions between MAC protons Ha, Hb, and Hd and the selectively excited He proton (see Figure 1 for structural assignment).

This is corroborated by the calculated interatomic distances in the simulated MAC conformations. In the *open* conformation, Hd and He are separated by 11.1 Å, too distant to produce measurable ROESY signals. In contrast, in the *closed* structure, this distance is around 5.7 Å, which is at the limit of ROESY detection and consistent with the low-intensity but observable ROESY signals. Thus, computational observations and ^1^H-ROESY identify and indicate the presence of the *closed* conformation in solution, its equilibrium with the *open* form, and its enhanced population at lower temperatures.

Consequently, the *closed* macrocycle conformation also has to be considered when investigating the observed chiral recognition. Thus, the docking of (*R*,*S*)-enantiomer to this new *closed* geometry was done, followed by the same sampling and refinement protocol as for the *open* conformation. The final ensembles contained distinct homochiral and heterochiral complexes (see Supporting Information: Table S15). The computed chemical shift differences and relative binding free energies (Table 2 and Supporting Information: Section 4.2–4.4) for the enantiomer complexes with the *closed* macrocycle successfully reproduce the experimentally observed trends in Δ*δ* and Δ*G*_*bind*_ (derived from Δ*D*) simultaneously. The (*R*)-enantiomer exhibits greater shielding (except for Mosher’s acid). The complexation of (*S*)-enantiomer is thermodynamically favored, which can explain the measured lower diffusion coefficient due to a lower Gibbs free energy of binding for (*S*)-stereoisomer in complex with (*R*)-MAC. The case of MA binding to the *open* MAC conformer could not reproduce the thermodynamic preference of (*S*)-MA complexation inferred from the diffusion experiments. In contrast, the calculated relative binding free energies with the *closed* form consistently reproduce the experimental preference for the heterochiral complex for all examined analytes in which experimental enantiodifferences were observed. Thus, only the *closed* receptor conformer reproduces both the observed diffusional and chemical shift orderings, providing a consistent model for rationalizing the observed stereodiscrimination.

Figure 5A and B depict the lowest-energy homochiral and heterochiral complexes between mandelic acid enantiomers and the *closed* MAC state. Unlike the *open* form, the central binding site cavity for (*R,S*)-mandelic acid molecules in the *closed* macrocycle is absent. Instead, in the compact receptor structure, both MA enantiomers form a hydrogen bond around MAC as the dominant coordination mode. For the homochiral complex, intermolecular interactions are often limited to a single hydrogen bond and van der Waals interactions, indicating an inherently weaker association. In the heterochiral complex, three distinct hydrogen bonds are observed, which are supported by additional van der Waals interactions. This explains the favorable binding of the (*S*)-MA.

**Figure 5:**
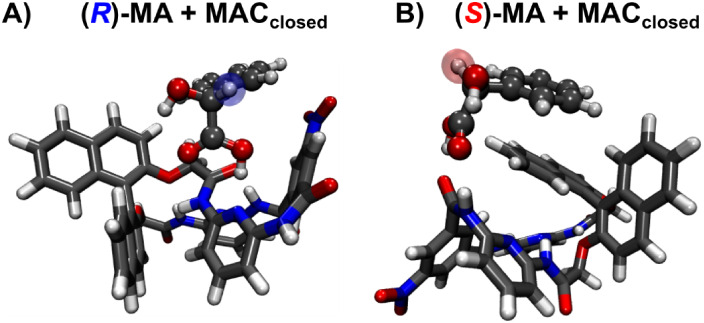
Lowest-energy structures of the complexes formed between the chiral macrocycle (*closed* conformation—MAC*_closed_*) and A) (*R*)-Mandelic Acid, B) (*S*)-Mandelic Acid (MA). Highlighted atoms represent the hydrogens at the chiral center of each MA enantiomer.

A second notable structural feature arises from the distinct orientations adopted by the hydrogen atom at the chiral center of each mandelic acid enantiomer. For the (*S*)-MA, the chiral hydrogen is solvent-exposed, whereas in (*R*)-MA it is shielded and directed toward the macrocycle, which can promote a stronger shielding effect. Both binding modes are fully consistent with those reported for similar chiral recognition systems, such as the discrimination of the MA enantiomer with BINOL, where an increased number of hydrogen-bonding contacts and specific proton orientations govern the enantiodifferentiation observed in NMR experiments.^19^ Furthermore, these two different binding modes can explain the apparent discrepancies observed between the separations in the experimental frequency and diffusion dimensions (see above), which could not be resolved beforehand.

Additional evidence for the importance of the *closed* MAC conformation is also provided by variable-temperature ^1^H NMR and 2D ^1^H–^1^H-EXSY experiments on enantiomeric mixtures of mandelic acid in the presence of the chiral macrocycle. Although the macrocycle signals do not resolve into distinct resonances for the *open* and *closed* conformations at any of the temperatures investigated (providing an initial indication that these species are in fast exchange on the NMR time scale), the ^1^H–^1^H EXSY contour maps (Supporting Information: Section 3.5) exhibit exchange cross-peaks between He and Hd atoms of the chiral macrocycle (Figure 1). These cross-peaks are consistent with chemical exchange and may originate from an equilibrium between the *open* and *closed* macrocycle conformations.^53^ Although these experiments are close to the limits of spectral resolution and sensitivity, they qualitatively support the existence of an equilibrium between the two conformational states, consistent with the ROESY and variable-temperature ^1^H NMR results obtained for the free macrocycle.

Moreover, the separation between the ^1^H signals of (*R*)-MA and (*S*)-MA in variable-temperature ^1^H NMR measurements provides further evidence that supports the enantiomer coordination to the *closed* macrocycle conformation (Figure 6).

**Figure 6:**
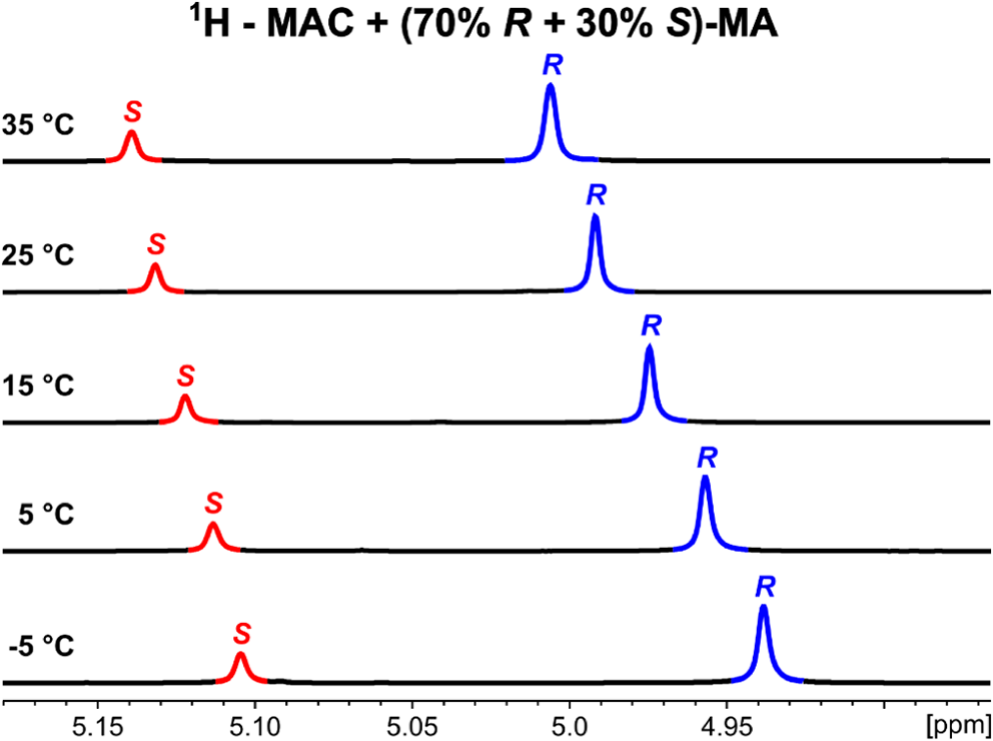
Selected spectral regions from 600.17 MHz ^1^H NMR depicting the chemical shift separation between (*R*)-enantiomer and (*S*)-enantiomer of mandelic acid (MA) in complex with the chiral macrocycle (MAC) as a function of temperature (from −5 to 35 °C).

The enantiodifference in the chemical shifts (Δδ) of the mandelic acid stereoisomer increases with decreasing temperature (from −80.2 Hz to −99.8 Hz), as shown in Figure 6 and Table S14 (Supporting Information). This behavior can be expected since lower temperatures shift the conformational equilibrium toward the thermodynamically favored diastereomeric complex. When the *closed* form is the predominant conformation for discriminating the mandelic acid enantiomers, the separations are also enhanced as the temperature decreases. The increase in shielding is supported by the calculated distances: in MA-MAC complexes, He-Hd separations range from 11.35 Å to 11.39 Å for the *open* form but reduce to to 8.19 Å–8.63 Å for the *closed* form (Supporting Information, Section 4.5).

Further relevant experimental evidence concerns the interaction between each mandelic acid enantiomer and the chiral macrocycle. For the first time, selective 1D ^1^H-ROESY measurements (Supporting Information, Section 3.4) revealed spatial proximity and, therefore, intermolecular association between both (*R*)-MA and (*S*)-MA with the chiral macrocycle. When the proton at the chiral center of MA was selectively excited, the ROESY signals were detected with He, as well as with multiple aromatic protons in the 7.0–10.5 ppm region of the MAC, suggesting the possibility of further interactions with the π-electron density of the macrocycle.

All of these experimental observations are fully consistent with our computational results and with the presence of close contacts between mandelic acid and the macrocycle in solution, driven by hydrogen bonding and van der Waals interactions. The presented results go beyond previous reports by demonstrating that the conformational equilibrium of the macrocycle governs stereoselective behavior. The dominant conformer of the macrocyclic receptor is not the one obtained in single crystals but rather an additional conformer identified in a liquid solution at room temperature. Since this recognition mechanism arises from the intrinsic flexibility of the host rather than from specific features of the analytes, the combined computational and experimental framework developed here is expected to be applicable to a range of more systems in future diffusion NMR studies employing this chiral macrocycle.

## Conclusions

Chiral resolving agents, such as chiral macrocycles, are capable of distinguishing enantiomers in solution at room temperature. In this work, we demonstrate that combining such macrocycles with diffusion NMR enables both the assignment of absolute configurations and gives insight into chiral recognition within a single, rapid experiment, demonstrating the versatility and strength of NMR relative to other analytical techniques.

Upon complexation with the macrocyclic receptor, mandelic acid stereoisomers exhibit substantial chemical shift separation but only moderate differences in diffusion coefficients, with the (*S*)-MA enantiomer displaying a larger chemical shift and a lower diffusion coefficient than the (*R*)-MA stereoisomer. The trends in diffusional separation were consistent across all analytes for which enantioselective discrimination was feasible, and thus confirming the ability of this macrocycle to enable the assignment of absolute configurations in solution by NMR. The pronounced frequency separation (most pronounced for mandelic acid) further suggests potential applications in enantiomeric excess determination, particularly when compared with classical resolving agents such as BINOL, where chemical shift differences are markedly smaller. However, our results show that the binding of stereoisomers to the *open* receptor conformation, as suggested in previous studies, does not account for the moderate differences in the diffusion coefficients. It is unexpected to observe a substantial increase in chemical shift separation relative to that of the smaller BINOL but only a moderate increase in diffusional separation. This shows that the design of larger macrocyclic receptors with more tentative sites for analyte binding does not necessarily lead to enhanced separation in the diffusional regime. Rather, the synthetic incorporation of more interaction sites also increases the number of rotatable bonds and intrinsic receptor flexibility.

Analysis of the macrocyclic conformational space reveals an equilibrium between the *open* and *closed* MAC states, which was confirmed by temperature-dependent ^1^H and selective 1D-ROESY measurements. The compact *closed* conformation is characterized by a reduced hydrodynamic radius in solution, which rationalizes the moderate increase in diffusion discrimination. Our computational results show that complexes formed by the *closed* state and a series of chiral analytes provide a consistent explanation for both the experimentally observed diffusion trends (which reflect the thermodynamics governing chiral complex formation) and the chemical shift differences.

The central importance of conformational preferences in stereodifferentiation underscores that effective resolving agents must be carefully designed not only with respect to their size and number of interaction sites but also their degrees of flexibility. Their ability to fold, reorganize, and adopt compact, stereochemically relevant arrangements is another key determinant of describing how efficiently enantiomers are recognized, differentiated, and separated. The crystallized state of the receptor is not necessarily the only one in liquid solution, and the number of accessible conformers should also be considered. In this work, it is demonstrated that sophisticated NMR studies have to be augmented by an extensive computational exploration of the increasing conformational degrees of freedom for complex analytes and complex receptor molecules. These aspects will enhance the mechanistic understanding when developing the next generation of chiral macrocycle-resolving agents for absolute configuration assignment.

## Methods

All compounds were commercially available and used without further purification. NMR samples were prepared using 30 mM of the chiral macrocycle (MAC)—CHIRABITE-AR—and 30 mM of an enantiomeric mixture in 500 μL (for mandelic acid—MA) and in 150 μL (for 2-butanol, camphor, α-methylbenzylamine, methyl mandelate, and Mosher’s acid) of deuterated chloroform (CDCl_3_) containing 0.03% (v/v) tetramethylsilane (TMS) as an internal reference. The enantiomeric mixture consisted of approximately 70% (*R*)-enantiomer and 30% (*S*)-enantiomer.

The NMR measurements were conducted at 298 K. Most experiments were recorded using a Bruker Avance NEO spectrometer operating at 600.17 MHz for ^1^H and equipped with a CryoProbe TCI Prodigy. The diffusion-NMR measurements were performed on a Bruker Avance III operating at 499.87 MHz for ^1^H, equipped with a BBO probe and a *z*-gradient system with a maximum nominal gradient strength of 50.3 G cm^–1^. Additional 2D experiments, including ^1^H–^1^H COSY, multiplicity-edited ^1^H–^13^C HSQC (decoupling during acquisition), ^1^H–^13^C HMBC, 2D ^1^H–^1^H ROESY, variable-temperature ^1^H NMR, ^1^H–^1^H EXSY, and selective 1D ^1^H ROESY were acquired following standard Bruker routines. Diffusion experiments were performed using the ^1^H-Oneshot^54^ and ^19^F-Oneshot^55^ pulse sequences with 16 gradient increments, where the gradient amplitude increased quadratically from 10% to 80% of the maximum nominal gradient. For each increment, 16 scans, 16 dummy scans, and 32k points were recorded. The diffusion delay (Δ) and gradient duration (*δ*) were optimized to achieve approximately 80% signal attenuation between the first and last increment.

Diffusion data sets were processed using GNAT v1.2.3 and v2.1,^56^ applying Fourier transformation with Lorentzian and/or Gaussian apodization, with the line-broadening parameters optimized individually for each analyte to achieve adequate spectral resolution and reliable fitting of the diffusion decay curve.^57^ Manual and individual phase and baseline adjustments were also employed. Diffusion coefficients and associated uncertainties were obtained by monoexponential fitting of the modified Stejskal-Tanner equation.^57^ Further experimental details, acquisition parameters, and specifications of processing procedures are provided in Supporting Information, section 1.

The initial structures of the chiral macrocycle and the (*R,S*)-enantiomers of each of the six analytes were constructed in Avogadro and preoptimized using the semiempirical GFN2 method as implemented in the xTB program.^44,46^ The diastereomeric complexes were then generated using the automated docking workflow implemented in xTB (automated interaction site screening-aISS),^47^ employing the ALPB implicit solvent model for chloroform.^58^ The lowest-energy docked complex structure for each homochiral and heterochiral complex was selected for further complex conformational sampling.

The conformational search was carried out using CREST 3.0.2^45^ with noncovalent interactions enabled and ALPB implicit solvent. Sampling was performed using iterative metadynamics and molecular dynamics simulations, combined with genetic crossing, to explore low-energy conformations extensively. Structures from CREST were refined by geometry optimization, followed by single-point Hessian evaluation to obtain thermochemical corrections at the semiempirical level (GFN2-xTB). High-energy and redundant structures were removed using RMSD, rotational constant, and relative energy filtering (Conformer-Rotamer Ensemble GENeration—CREGEN procedure).^45,59^ The remaining conformers were clustered using principal component analysis, followed by k-means clustering to reduce the ensemble to representative structures.^19,59,60^ The same conformational search protocol was applied to explore the intrinsic conformational space of the chiral macrocycle and identify the *closed* conformer.

The clustered ensembles were submitted to a DFT refinement using the CENSO 1.2.0 workflow^43^ and the ORCA 5.0.4 program^61^ through a structured four-stage procedure, from which Gibbs free energies and NMR shielding constants were extracted. In the first stage (PART 0), electronic energies were evaluated using single-point calculations at the B97-D3/def2-SV­(P)+gCP level, and solvation effects were described using the ALPB model at the GFN2-xTB level. In PART 1, these energies were recomputed using r^2^SCAN-3c/def2-mTZVPP together with the SMD (Solvation Model based on Density) implicit solvent. The SMD solvation model was employed to approximate the solvation effects of chloroform through a continuum description and to maintain consistency with the experimental solvent conditions. Although implicit solvation does not explicitly account for specific solvent–solute interactions, this approximation is expected to provide a reliable description of relative energetic trends in a nonpolar medium such as CHCl_3_, while reducing the computational cost compared to explicitly solvation.^62^ Moreover, in our previous studies, we have shown that the choice of implicit solvation model does not significantly affect the description of chiral recognition mechanisms in similar systems.^19^ At this second stage, single-point Hessian (SPH) calculations were also performed at the GFN2-xTB/ALPB level, employing the modified rigid-rotor harmonic oscillator (mRRHO) approximation to obtain thermochemical corrections and preliminary Gibbs free energies.^63,64^

In the third phase (PART 2), each structure was reoptimized twice: first using r^2^SCAN-3c/def2-mTZVPP, followed by a full geometry optimization at the ωB97X-V/def2-TZVPP level, both performed with SMD solvation. Updated thermostatistical corrections were again determined after each reoptimization, as described in PART 1. In the final step (PART 3), single-point DFT calculations were performed using ωB97X-V/def2-TZVPP with SMD solvation and thermochemical corrections. The NMR chemical shifts (δ) and binding Gibbs free energies were computed at the same level of theory for the low-lying diastereomeric complexes in the final ensemble. All reported energetic values and NMR parameters correspond to Boltzmann-weighted averages taken over the final conformer set.

Additional details regarding the theoretical workflow, including preoptimization steps, diastereomeric complex generation, conformational sampling, DFT refinement, and data analysis, are provided in the EU Open Research Repository zenodo (see Data Availability Statement).

## Supplementary Material



## Data Availability

All NMR acquisition files, computational results for macrocycle and enantiomer initial and refined structures, and main output files are available at an open public repository at 10.25824/redu/ZL36LD.

## Notes

Additional experimental and computational data supporting the findings of this study are provided in the Supporting Information. This includes full NMR acquisition parameters, contour plots and spectra, diffusion measurement data, selective ROESY analyses, docking results, conformational sampling data, and an extended description of the DFT refinement protocol. Information on the workflow architecture, CREST sampling configurations, and CENSO refinement steps is also provided. Further details on Gibbs free energies, binding free energies, computed NMR chemical shifts, and intermolecular distances are provided to ensure complete transparency and reproducibility of this work (PDF).
