# Supplementary material for: Macrocycle Conformational Flexibility as a Key to Enantioselective Recognition
Source: JACS Au. 2026 Jul 13;6(7):4128–37. doi: 10.1021/jacsau.6c00640 (PMC13417255; doi:10.1021/jacsau.6c00640)
Supplement: Supplementary file 1 [file au6c00640_si_001.pdf]

Supporting Information

Macrocycle Conformational Flexibility as a Key to Enantioselective Recognition

Tadeu Luiz Gomes Cabral[a,b], Matthias Stein[*,b], Claudio F Tormena[*,a]

[a]Institute of Chemistry, Physical Organic Chemistry Lab, University of Campinas - UNICAMP, Campinas - São Paulo, Brazil
[b]Max Planck Institute for Dynamics of Complex Technical Systems, Molecular Simulation and Design Group, Magdeburg, Germany

Contents

1 Detailed Experimental Procedures **S3**

2 Detailed Computational Procedures **S5**

3 Further Information on NMR Results **S7**

3.1 Characterization data S7

3.2 Experimental Diffusion Data S19

3.2.1 General Trends in the Experimental Enantiomeric Discrimination S19

3.2.2 Mandelic Acid S20

3.2.3 2-butanol S21

3.2.4 Camphor S23

3.2.5 Methylbenzylamine S25

3.2.6 Mosher's Acid S27

3.2.7 Methyl Mandelate S29

3.3 Temperature Variation Measurements S30

3.4 Additional Selective ROESY Experiments S33

3.5 Variable-Temperature 2D ^{1}H-^{1}H EXSY Experiments S34

3.5.1 EXSY Experiments at 50.0 °C S34

3.5.2 EXSY Experiments at 25.0 °C S36

3.5.3 EXSY Experiments at -25.0 °C S38

4 Further Information on Computational Results **S39**

4.1 Number of Diastereomeric Complex Conformers S40

4.2 General Trends in Computed Enantiomeric Discrimination S41

4.3 Diastereomeric Binding and Gibbs Free Energies S43

4.3.1 Refined Ensemble of the (*R*,*S*)-MA in Complex with the *Open* MAC S43

4.3.2 Refined Ensemble of the (*R*,*S*)-MA in Complex with the *Closed* MAC S43

4.3.3 Refined Ensemble of the (*R*,*S*)-2-Butanol in Complex with the *Open* MAC . S44

4.3.4 Refined Ensemble of the (*R*,*S*)-2-Butanol in Complex with the *Closed* MAC . S45

4.3.5 Refined Ensemble of the (*R*,*S*)-Camphor in Complex with the *Open* MAC . . S45

4.3.6 Refined Ensemble of the (*R*,*S*)-Camphor in Complex with the *Closed* MAC . S46

4.3.7 Refined Ensemble of the (*R*,*S*)-Methylbenzylamine in Complex with the *Open* MAC S47

4.3.8 Refined Ensemble of the (*R*,*S*)-Methylbenzylamine in Complex with the *Closed* MAC S47

4.3.9 Refined Ensemble of the (*R*,*S*)-Mosher's Acid in Complex with the *Open* MACS48

4.3.10 Refined Ensemble of the (*R*,*S*)-Mosher's Acid in Complex with the *Closed* MAC S48

4.4 Calculated NMR Chemical Shifts S49

4.4.1 Refined Ensemble of the (*R*,*S*)-MA in Complex with the *Open* MAC S49

4.4.2 Refined Ensemble of the (*R*,*S*)-MA Complex with the *Closed* MAC S50
4.4.3 Refined Ensemble of the (*R*,*S*)-2-butanol in Complex with the *Open* MAC . . S50
4.4.4 Refined Ensemble of the (*R*,*S*)-2-butanol Complex with the *Closed* MAC . . S51
4.4.5 Refined Ensemble of the (*R*,*S*)-Camphor in Complex with the *Open* MAC . . S51
4.4.6 Refined Ensemble of the (*R*,*S*)-Camphor Complex with the *Closed* MAC . . S52
4.4.7 Refined Ensemble of the (*R*,*S*)-Methylbenzylamine in Complex with the *Open* MAC S53
4.4.8 Refined Ensemble of the (*R*,*S*)-Methylbenzylamine Complex with the *Closed* MAC S53
4.4.9 Refined Ensemble of the (*R*,*S*)-Mosher's Acid in Complex with the *Open* MACS54
4.4.10 Refined Ensemble of the (*R*,*S*)-Mosher's Acid Complex with the *Closed* MAC S54
4.5 Interatomic Distances for Selected Hydrogen Atoms of the MAC S55
4.6 Gibbs Free Energy of the Free *Open* and *Closed* MAC S55

1 Detailed Experimental Procedures

All compounds were commercially available and used without further purification. NMR samples were prepared by combining stock solutions to yield a final mixture composed of 30 mM of the chiral macrocycle (MAC) and 30 mM of an enantiomeric mixture of mandelic acid (MA; containing approximately 70% (*R*)-MA and 30% (*S*)-MA) in a total volume of 500 µL of deuterated chloroform ($CDCl_3$) containing 0.03 v/v% tetramethylsilane (TMS) as the internal standard. The appropriate volumes of each stock solution were transferred to a plastic microtube and mixed for 90 sec. The resulting solution was then transferred to a 5 mm NMR tube for the NMR analysis.

For the analytes 2-butanol, camphor, α-methylbenzylamine, methyl mandelate, and Mosher's acid, for which only diffusion-NMR experiments were performed, the samples were prepared by dissolving each analyte at a concentration of 30 mM (with an enantiomeric composition of approximately 70% (*R*)-enantiomer and 30% (*S*)-enantiomer) together with 30 mM of the chiral macrocycle in a total volume of 150 µL of deuterated chloroform ($CDCl_3$) containing 0.03% v/v tetramethylsilane (TMS) as an internal standard. Sample preparation and mixing procedures were carried out following the same protocol described for Mandelic Acid. The resulting solutions were subsequently transferred to 3 mm NMR tubes for NMR analysis.

The NMR measurements were performed at 298 K (except when the temperature was changed). Most experiments were conducted on a Bruker Avance NEO spectrometer operating at 600.17 MHz for ^{1}H and equipped with a CryoProbe Prodigy TCI probe. The diffusion experiments were recorded on a Bruker Avance III spectrometer operating at 499.87 MHz for ^{1}H equipped with a BBO probe. The BBO probe featured a z-gradient coil with a maximum nominal gradient strength of 50.3 G cm^{-1}.

The ^{1}H NMR spectrum was acquired with a spectral width of 10000 Hz, a transmitter frequency offset of 3000 Hz, 32 scans, and 64k data points. The processing was carried out with 128k points and a line broadening of 0.3 Hz. The ^{13}C{ ^{1}H } NMR spectrum was recorded with a spectral width of 31250 Hz, a transmitter offset of 14650 Hz, 2048 scans, 4 dummy scans, and 64k data points, using a *WALTZ65* decoupling with 0.096857 W of power level. The data were processed with 128k points and a line broadening (LB) of 2 Hz.

The 2D ^{1}H–^{1}H COSY experiment was acquired with spectral widths of 10000 Hz in both dimensions, a transmitter offset of 3000 Hz, 16 scans, 16 dummy scans, 2048 data points in the direct dimension, and 256 increments. Non-uniform sampling (NUS) of 25% was used with 128 hypercomplex points in the indirect dimension. The COSY data were processed with 1024 points and zero line broadening in each dimension.

The 2D ^{1}H–^{13}C multiplicity-edited HSQC experiment was acquired with a direct-dimension spectral width of 10000 Hz, transmitter offset of 3000 Hz, 8 scans, 16 dummy scans, 4096 data points, and a *bi_p5m4sp_4sp.2* decoupling (power level of 7.9139 W) with CNST2 = 145 (one-bond $^1J_{CH}$ coupling constant - used to optimize magnetization transfer between ^{1}H and ^{13}C during the HSQC pulse sequence). The indirect dimension used a spectral width of 31250 Hz, a transmitter offset of 14650 Hz, and 256 increments. NUS of 25% with 128 hypercomplex points was applied. The HSQC was processed with 32k points in the direct dimension (QSINE window function, LB = 0

Hz) and 2048 points in the indirect dimension.

The 2D ^1H–^{13}C HMBC experiment was acquired with a direct-dimension spectral width of 10000 Hz, offset 3000 Hz, 32 scans, 16 dummy scans, 4096 data points, and CNST13 = 8 (corresponds to the long-range $^{2-3}J_{CH}$ coupling constant). The indirect dimension used a spectral width of 31250 Hz, offset 14650 Hz, and 256 increments. NUS of 25% with 128 hypercomplex points was applied. The HMBC processing employed 16k points in the direct dimension (QSINE window function, LB = 0 Hz) and 512 points in the indirect dimension.

The 2D ^1H–^1H EXSY experiments were acquired with spectral widths of 11 904.76 Hz in both dimensions, a transmitter offset of 3701.25 Hz, 8 scans, 16 dummy scans, 4096 data points in the direct dimension for acquisitions at −25 °C and 50 °C, and 2048 data points for acquisitions at 25 °C, and 512 increments in the indirect dimension. For these experiments, the NOESY pulse sequence (*noesygpphpp*, Bruker) was employed with mixing times ranging from 500 to 3000 ms. The EXSY data were processed with 8192 points in the direct dimension (QSINE window function, LB = 0.3 Hz) and 1024 points in the indirect dimension, followed by automatic phase and baseline correction.

The 2D ^1H–^1H ROESY experiment was acquired with spectral widths of 10000 Hz in both dimensions, transmitter offset 3000 Hz, 16 scans, 16 dummy scans, 4096 data points in the direct dimension, and 846 increments. The presaturation power level was 2.5315×10^{-5} W, and the pulsed-spinlock ROESY mixing field was applied at 0.078132 W. The ROESY data were processed with 8192 points in the direct dimension (QSINE window function, LB = 0 Hz) and 1024 points in the indirect dimension.

The 1D ^1H selective ROESY experiment was acquired with a spectral width of 10000 Hz, transmitter offset 3000 Hz, 1024 scans, 4 dummy scans, and 32k data points, with selective excitation centered at ~4.53 ppm. The 1D selective ROESY data were processed, containing 32k points with a line broadening of 0.5 Hz.

The diffusion experiments were performed using the ^1H-Oneshot[1] and ^{19}F-Oneshot[2] pulse sequence with 16 gradient increments, in which the gradient strength was varied quadratically from 10% to 80% of the maximum nominal gradient value. For each increment, 16 scans, 16 dummy scans, and 32k data points were acquired. The diffusion time (Δ, d20) and the gradient pulse duration (δ, p30) were optimized for each experiment to achieve approximately 80% attenuation between the first and last increment. As no internal reference was used for the ^{19}F-NMR experiments, the signal at a lower chemical shift was referenced to -70.0 ppm.

All diffusion data were processed using GNAT v1.2.3 and v2.1 software[3] running in MATLAB R2019a. Fourier transformation was performed using Lorentzian and/or Gaussian window functions, with 64k points and the line-broadening parameters optimized individually for each analyte to achieve adequate spectral resolution and reliable fitting of the diffusion decay curves.[4] This was followed by manual phase correction of each individual increment, together with manual baseline correction.[4] Diffusion coefficients and their associated uncertainties were determined by fitting a monoexponential decay function based on the modified Stejskal–Tanner equation to selected resonances identified by peak picking. When necessary, spectral regions were pruned from the fitting procedure and from the generation of the DOSY plots to improve and simplify the diffusion analysis.

2 Detailed Computational Procedures

The initial structures of the chiral macrocycle (MAC) and each enantiomer of mandelic acid, 2-butanol, camphor, α-methylbenzylamine, methyl mandelate, and Mosher's acid, were built in Avogadro[5] and pre-optimized using xTB 6.7.1[6,7]. Subsequently, the default xTB docking algorithm implemented in xTB 6.7.0,[6,8] employing the ALPB implicit solvent model for chloroform[9] and the semi-empirical tight-binding method GFN2-xTB,[7] was used to generate the diastereomeric complexes between MAC and each enantiomer (Homochiral complexes: (*R*)-MAC with (*R*)-enantiomer; Heterochiral complexes: (*R*)-MAC with (*S*)-enantiomer). This docking procedure is a recently developed automated protocol (automated computational interaction site screening - aISS) designed to efficiently generate molecular aggregate structures. It combines a genetic algorithm to perform the initial search with the xTB-IFF intermolecular force field, and subsequently refines structures using GFN-FF and GFN2-xTB geometry optimizations.[8]

From the ensemble of 15 docked structures, the lowest-energy diastereomeric complex for each Stereoisomeric analyte was selected for further conformer/rotamer sampling using the Conformer–Rotamer Ensemble Sampling Tool (CREST 3.0.2).[10,11] Sampling was performed at the GFN2-xTB level with the non-covalent interaction (NCI) mode enabled for non-covalent complexes, using the ALPB implicit solvation model for chloroform and an energy window of 6 kcal.mol^{-1}. In this step, the iterative metadynamics and genetic crossing (iMTD-GC) workflow was applied, in which metadynamics employing root-mean-square-deviation (RMSD) as collective variables and genetic *Z*-matrix crossing are used to explore the complex conformations.[11]

Low-lying conformers from the CREST sampling were then refined following a protocol analogous to that established in our previous work, which demonstrated that this workflow reproduces experimental stereoselectivity trends quite well.[12] Firstly, the conformers were re-optimized in CREST at the same theoretical level used for conformational sampling but with the optimization criteria set to *very tight*. Then, thermochemical properties were computed through single-point Hessian (SPH) calculations using the `-prop hess` option in CREST.[13] High-energy or redundant structures were removed by filtering based on RMSD, electronic energies, and rotational constants. The remaining conformers were automatically clustered using principal component analysis (PCA) followed by *k*-means clustering, as implemented in the CREST program, thereby reducing the ensemble to a representative subset for subsequent DFT refinement. Further statistical details on the clustering can be found in the CREST *output* files available in the online repository (DOI: 10.25824/redu/ZL36LD).

The refinement of the clustered ensembles was carried out using the Command-line ENergetic SOrting workflow (CENSO 1.2.0)[14] in combination with ORCA 5.0.4[15], employing a four-step protocol. In PART0, the electronic energies were obtained from single-point calculations at the B97-D3/def2-SV(P)+gCP level of theory, with solvation contributions estimated using the ALPB model at the GFN2-xTB level.

In the second step (In PART1), single-point energies were recalculated at the r^{2}SCAN-3c/def2-mTZVPP level, including solvation via the SMD model.[16] SPH calculations

using the modified rigid-rotor harmonic oscillator (mRRHO) model at the GFN2-xTB/ALPB level provided thermostatistical corrections and Gibbs free energies.[13]

In PART2 (third step), two consecutive geometry optimizations were performed: first, an initial optimization at r^{2}SCAN-3c/def2-mTZVPP, followed by full re-optimization at the ωB97X-V/def2-TZVPP level of theory, both employing the SMD solvation model. Thermochemical corrections were again calculated as described above.

The fourth and last step (PART3) consisted of final single-point DFT calculations at the ωB97X-V/def2-TZVPP level using the SMD solvation model and mRRHO thermochemical corrections at the GFN2-xTB/ALPB level from the xTB algorithm. At this stage, a more rigorous SCF convergence criterion (*scfconv7*) is applied relative to the previous steps. NMR chemical shifts (δ) were finally computed at the same level of theory, and binding Gibbs free energies were calculated for all structures in the final ensemble. The final computed ^{1}H NMR chemical shifts were obtained by referencing the isotropic shielding constants to tetramethylsilane (TMS), used as the standard reference ($\sigma_{\mathrm{TMS}} = 31.609$ ppm from DFT calculations), and calculating the corresponding chemical shifts for the investigated nuclei according to

$$\delta_i = \sigma_{\mathrm{TMS}} - \sigma_i \tag{1}$$

where δ_i is the calculated chemical shift of nucleus i, σ_i is its computed isotropic shielding constant, and σ_{TMS} is the isotropic shielding constant calculated for tetramethylsilane at the same level of theory. The enantiomeric discrimination in the frequency dimension was quantified by the difference in chemical shifts between the two enantiomers, defined as

$$\Delta\delta_{RS} = \delta_R - \delta_S \tag{2}$$

where δ_R and δ_S correspond to the calculated chemical shifts of the (*R*)-enantiomer and (*S*)-enantiomers, respectively.

For ^{19}F NMR, no internal reference was employed in the experimental measurements. To maintain consistency with the experimental protocol, the calculated ^{19}F chemical shifts were therefore analyzed directly in terms of relative shielding differences rather than referenced chemical shifts. In both cases, enantiomeric discrimination was quantified by the difference between the ^{19}F-NMR responses of the two enantiomers, defined as

$$\Delta\delta_{RS} = \delta_R - \delta_S = (\sigma_{\mathrm{ref}} - \sigma_R) - (\sigma_{\mathrm{ref}} - \sigma_S) \tag{3}$$

$$\Delta\delta_{RS} = \sigma_S - \sigma_R \tag{4}$$

All reported NMR properties and thermodynamic quantities also correspond to Boltzmann-weighted averages. Additionally, all implicit solvation models used throughout the workflow were employed to model chloroform as the solvent.

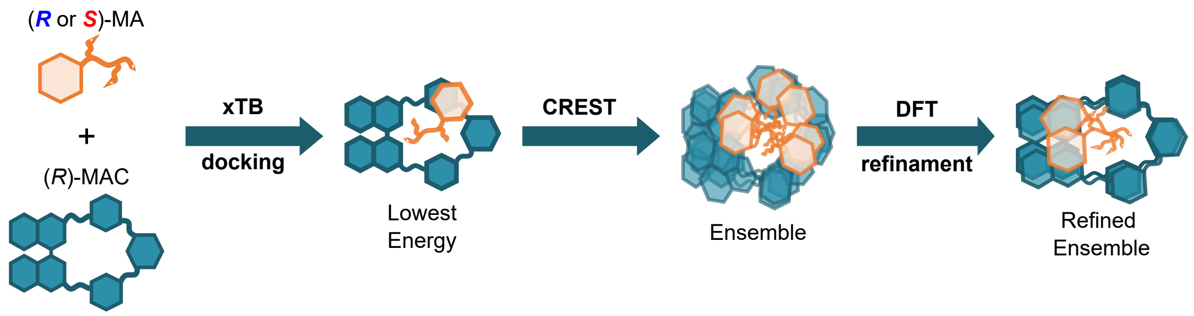

Figure S1: General workflow for generating the diastereomeric complexes between the chiral macrocycle (MAC) and each enantiomer of mandelic acid (MA) via xTB docking, followed by conformer/rotamer ensemble generation using CREST and subsequent refinement of these ensembles at the DFT level.

An overview of the workflow for conformer generation and subsequent refinement at both the semiempirical and DFT levels is shown in Figure S1. The number of unique conformers at each stage of the computational protocol is provided in Section S4.

For assessing the macrocycle conformations, the same protocol described above (CREST sampling) was applied. This approach has sampled the *closed* conformations that appeared as low-lying structures. Consequently, the lowest-energy *closed* conformation generated by the metadynamics simulations was selected as the initial structure for docking and subsequent refinement, as follows the workflow illustrated in Figure S1.

For understanding the equilibrium between the *open* and *closed* MAC conformations, the lowest-energy geometries of both forms were further refined at semi-empirical and DFT levels of theory. For the semi-empirical procedures, the geometries were optimized using the xTB program, and single-point Hessians were calculated with the CREST program, both at the GFN2/ALPB level of theory. For the DFT calculations, the lowest-energy MAC conformers sampled were further refined using the same CENSO quantum mechanical workflow described above. The main computational results are presented in Section S4.6.

3 Further Information on NMR Results

3.1 Characterization data

From the ^{1}H NMR spectrum (600 MHz, $CDCl_3$) of the chiral macrocycle, as displayed at Figure S3, several signals can be identified (chemical shifts in δ, ppm): δ 4.285 (d, J = 15.9 Hz, 2H); 4.56 (d, J = 16.0 Hz, 2H); 7.26 (s, $CHCl_3$ residual); 7.28 (d, J = 8.5 Hz, 2H); 7.39 (ddd, J = 8.2 Hz, 6.8 Hz, and 1.1 Hz, 2H); 7.44 (d, J = 9.0 Hz, 2H); 7.48 (ddd, J = 8.2 Hz, 6.8 Hz, and 1.1 Hz, 2H); 7.69 (t, J = 8.0 Hz, 2H); 7.88 (d, J = 8.2 Hz, 2H); 7.91 (d, J = 8.2 Hz, 2H), 7.98 (d, J = 9.0 Hz, 2H), 8.07 (d, J = 8.0 Hz, 2H), 8.64 (s, 2H); 8.73 (s, 1H); 9.04 (s, 2H); and 9.11 (s, 2H).

In this study, the signals at δ 4.28 (d, J = 15.9 Hz, 2H) and 4.56 (d, J = 16.0 Hz, 2H) are identified as diastereotopic protons belonging to H_e (Figure S2). This assignment is supported by the multiplicity-edited HSQC spectrum (Figure S7), in which only these protons exhibit the phase characteristic of a $-CH_2$ group. The HMBC spectrum (Figure S10) further confirms this attribution:

H_e displays a clear correlation with the carbonyl carbon at 167.63 ppm (C_x, Figure S2). The same carbon (C_x) also shows an HMBC correlation with the signal at 8.64 ppm (s, 2H), assigned to the amide proton H_b.

A second carbonyl carbon at 161.49 ppm is therefore assigned as C_y. This carbon (C_y) shows HMBC correlations with the resonances at 8.73 ppm (s, 1H), 9.04 ppm (s, 2H), and 9.11 ppm (s, 2H). Since the resonance at 9.11 ppm shows no HSQC correlation — consistent with the behavior of amide proton H_b (Figures S7 and S8) — yet displays an HMBC correlation with C_y, it must correspond to the remaining amide proton H_a (Figure S2). The resonances at 8.73 ppm and 9.04 ppm are therefore assigned to H_c and H_d, respectively. The integrals of these signals support this assignment.

The ROESY spectra (Figures S11 and S12) further corroborate these assignments by confirming the expected spatial proximity between H_a and H_c. The COSY spectra (Figures S5 and S6) and the ^{13}C NMR data (Figure S4) provide additional support for the final structural assignments.

A similar assignment strategy was employed to characterize the macrocycle resonances H_a, H_b, H_c, H_d, H_e, C_x, and C_y in the diastereomeric complexes formed with the mandelic acid enantiomers. The key spectroscopic data supporting these assignments are presented in Figures S13–S17. All raw NMR data, including the original FID files, are available in an openly accessible online repository at DOI: 10.25824/redu/ZL36LD.

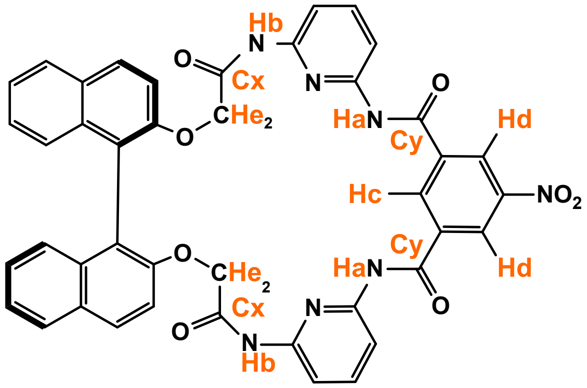

Figure S2: Chemical structure of the chiral macrocycle (MAC) with the carbon and hydrogen atoms highlighted as monitored in this study.

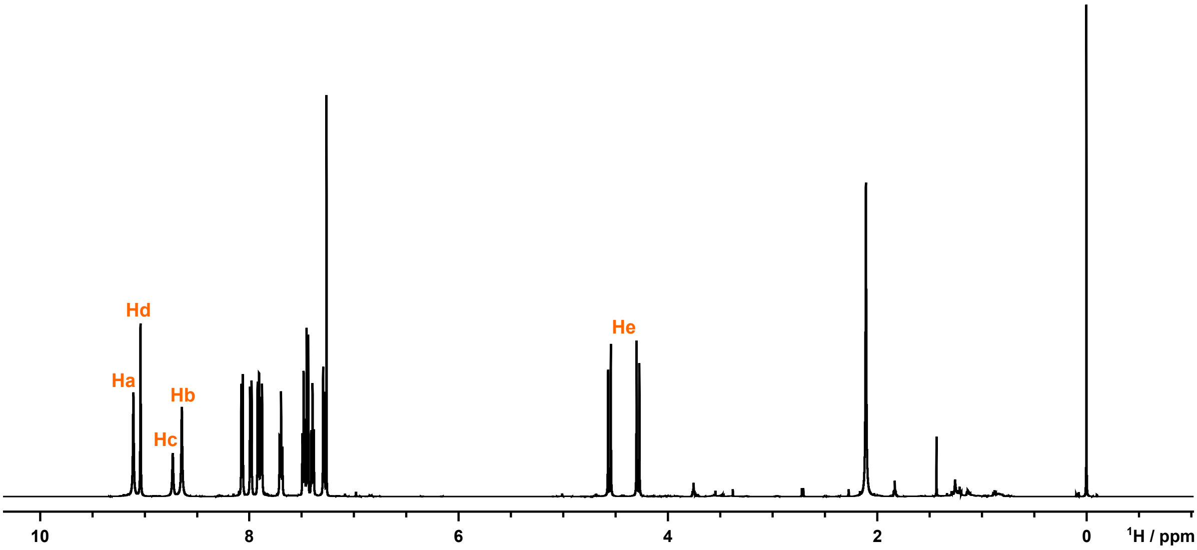

Figure S3: 600 MHz ^{1}H NMR spectrum of the chiral macrocycle in $CDCl_3$. The resonances assigned to protons H_a, H_b, H_c, H_d, and H_e are highlighted.

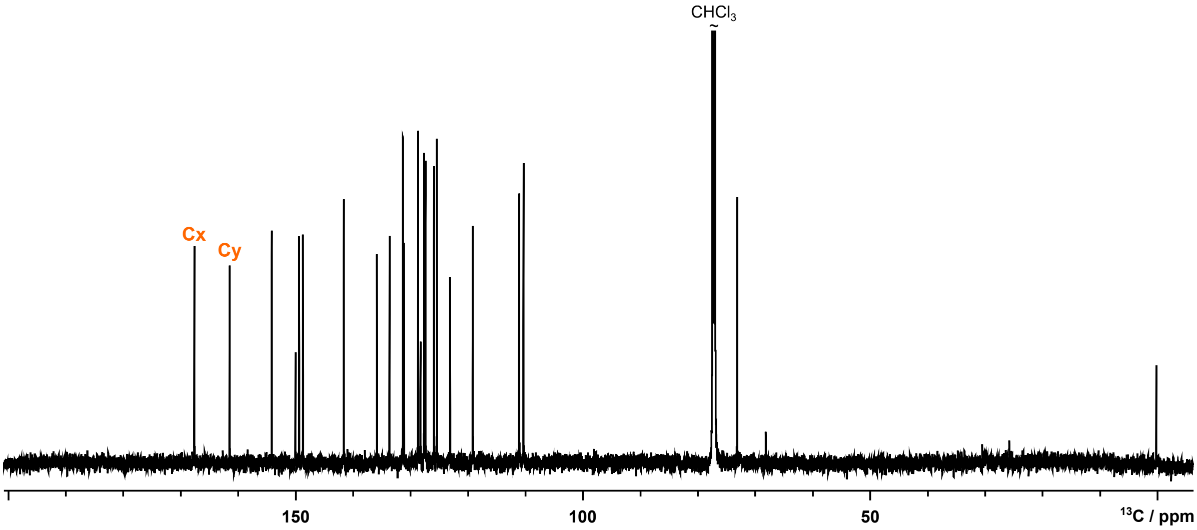

Figure S4: 150 MHz ^{13}C-{^{1}H} NMR spectrum of the chiral macrocycle in $CDCl_3$. The resonances assigned to protons C_x and C_y.

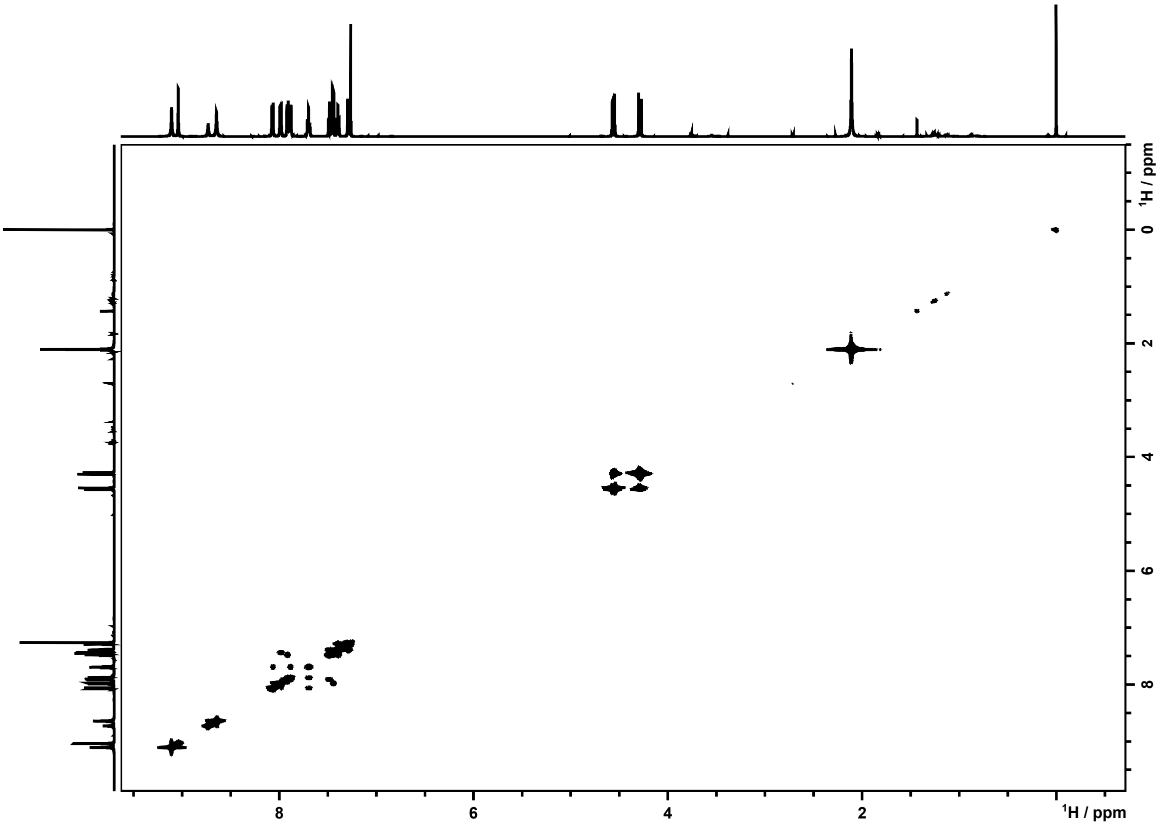

Figure S5: 600 MHz 2D ^1H–^1H COSY contour map of the chiral macrocycle in $CDCl_3$.

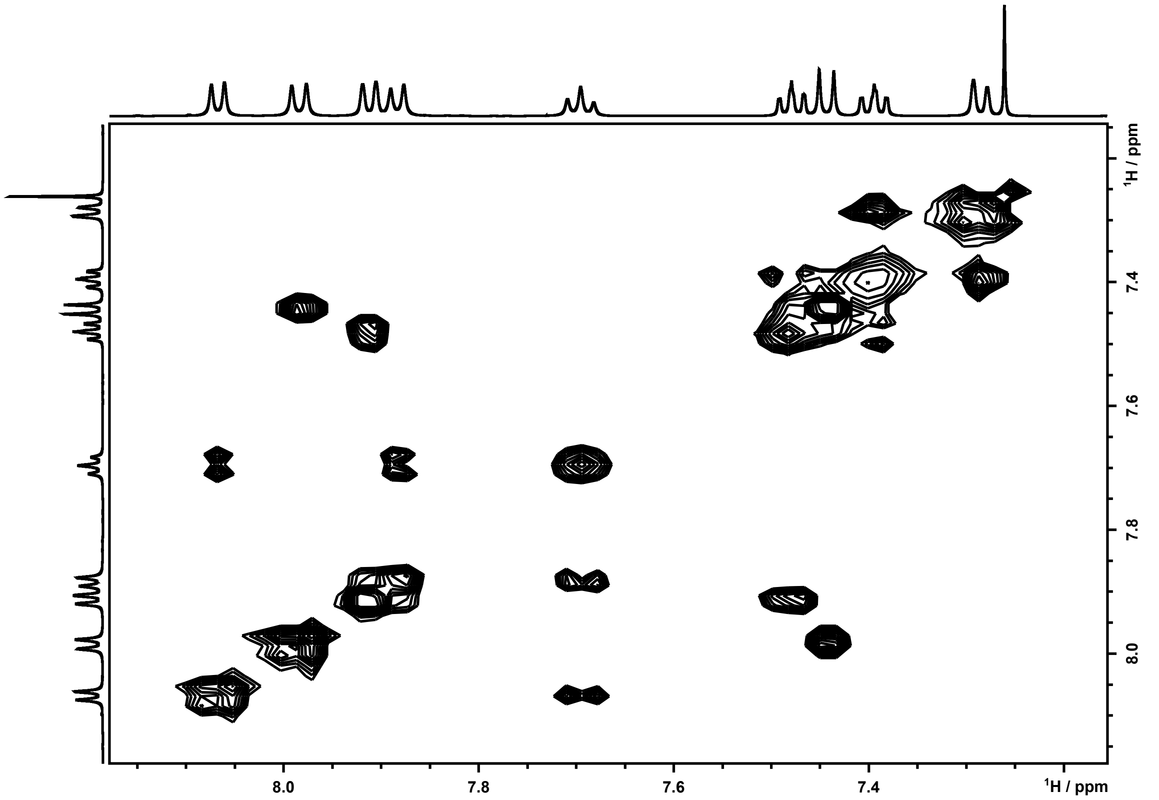

Figure S6: 600 MHz 2D ^1H–^1H COSY contour map showing the region from ~7.15 to ~8.20 ppm for the chiral macrocycle in $CDCl_3$.

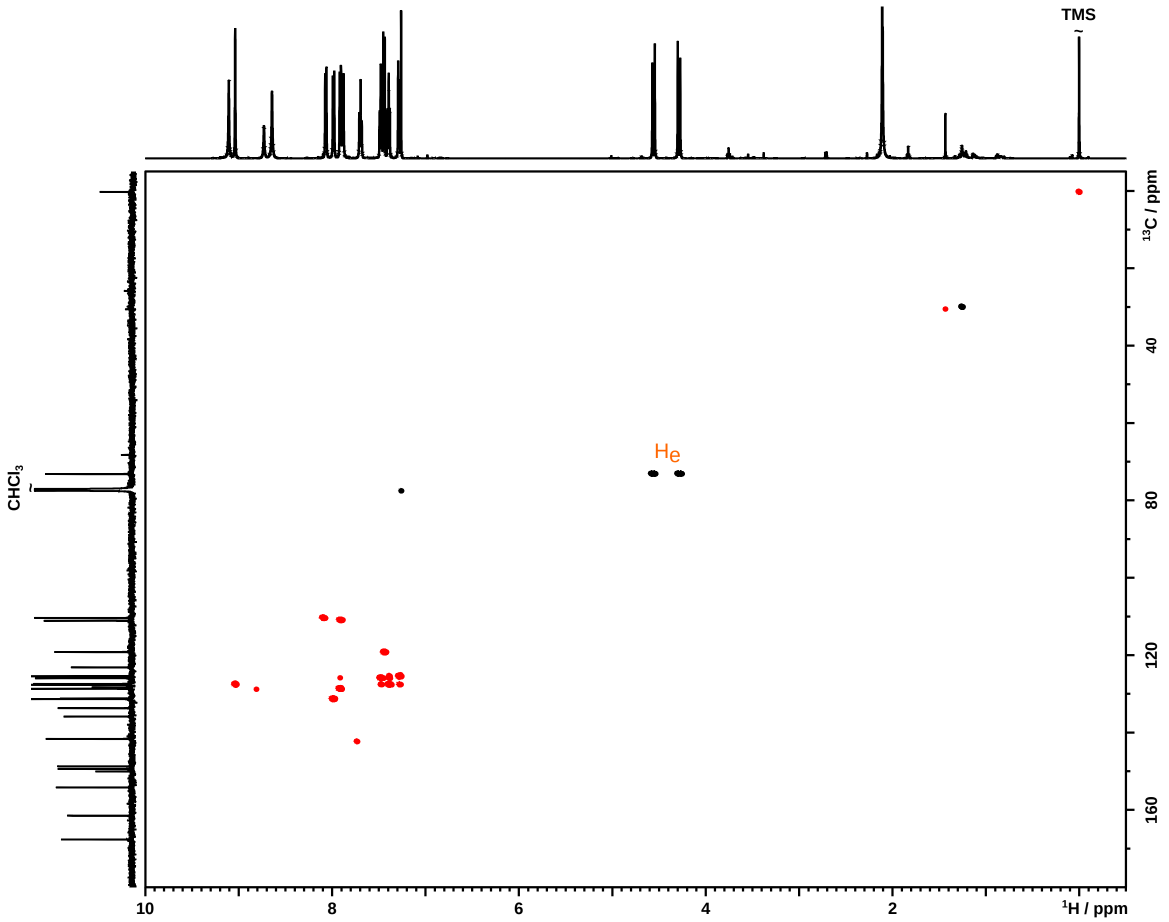

Figure S7: 600 MHz 2D ^{1}H–^{13}C multiplicity-edited HSQC contour map of the chiral macrocycle in $CDCl_3$. In the multiplicity-edited HSQC spectrum, signals in red correspond to CH_3 and CH groups, whereas signals in black indicate CH_2 groups. The highlighted correlation in the map corresponds to proton H_e of the macrocycle.

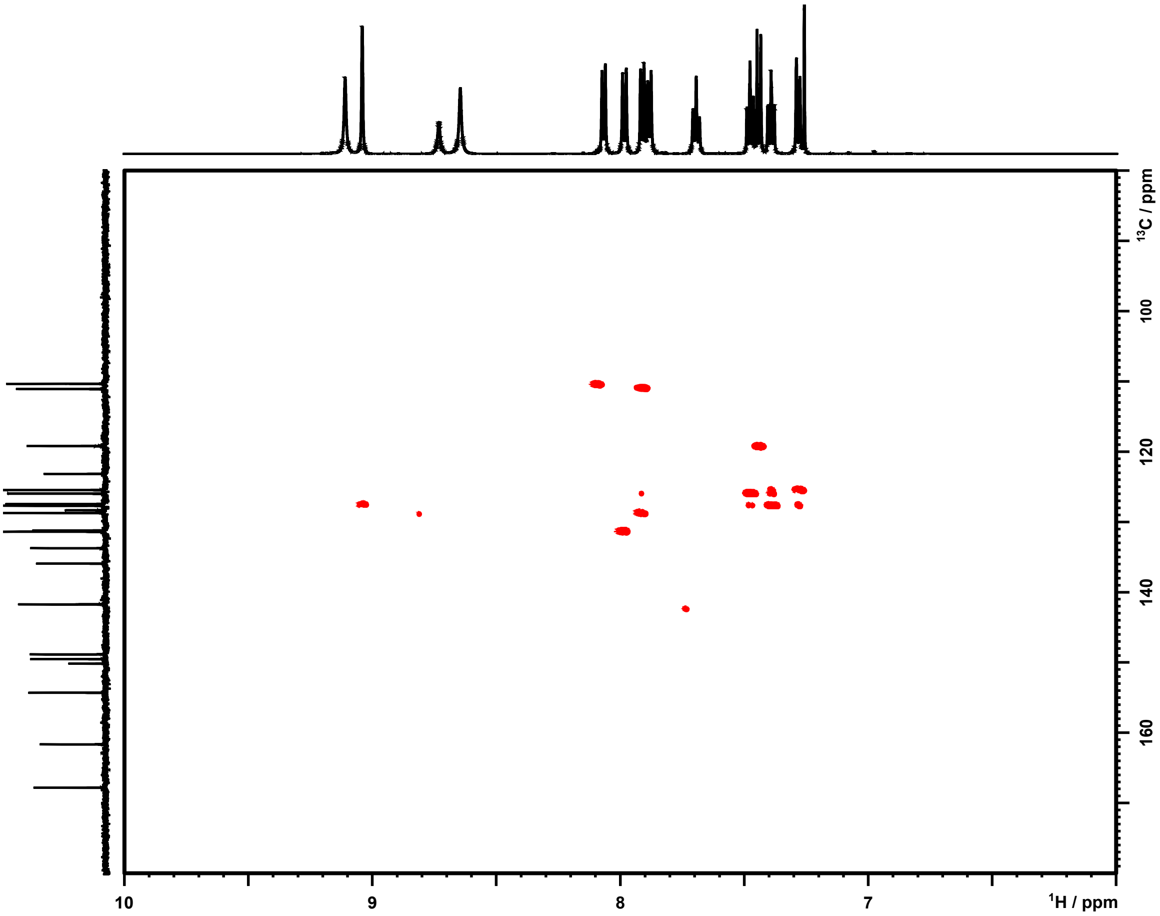

Figure S8: 600 MHz 2D ^{1}H–^{13}C HSQC contour plot showing the region from ~6.0 to ~10.0 ppm in the ^{1}H dimension and from ~80 to ~180 ppm in the ^{13}C dimension for the chiral macrocycle in $CDCl_3$.

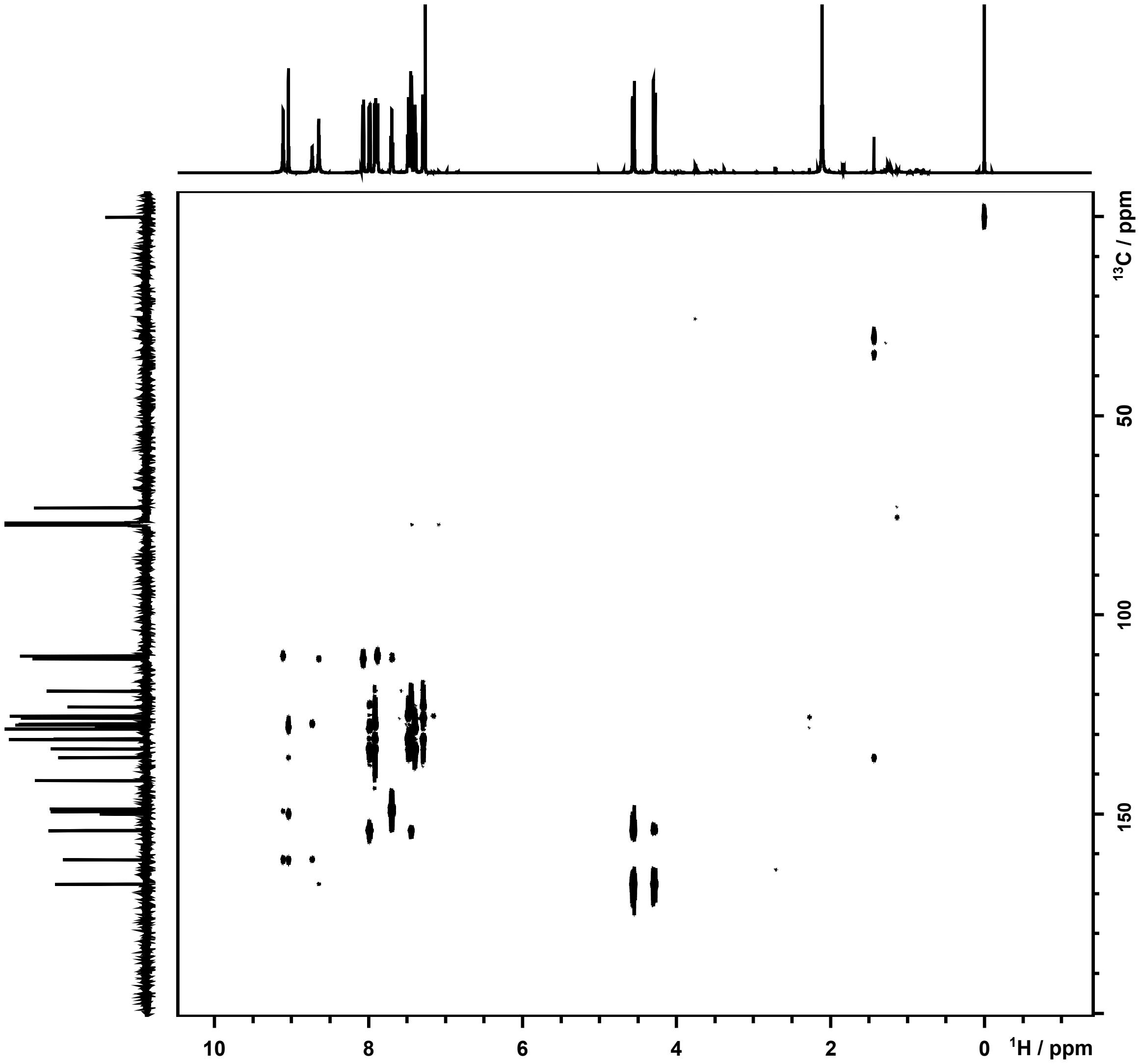

Figure S9: 600 MHz 2D ^{1}H–^{13}C HMBC contour map of the chiral macrocycle in $CDCl_3$.

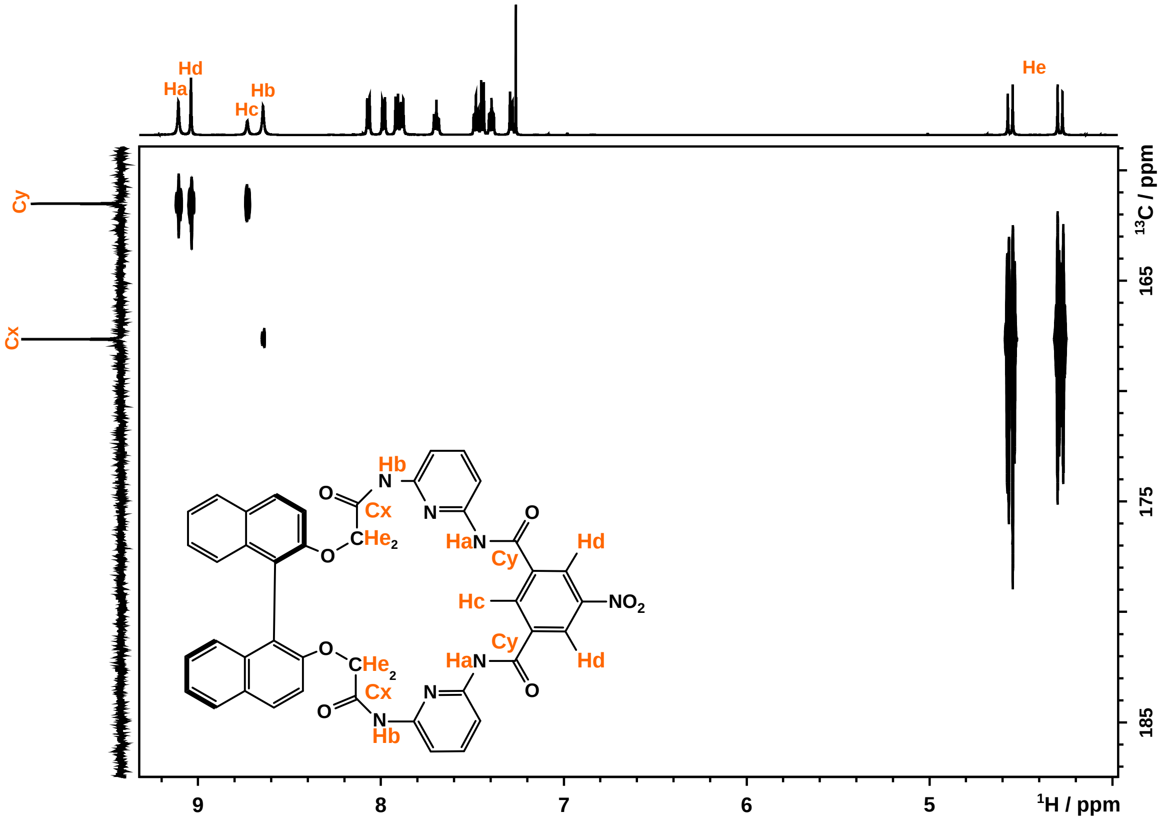

Figure S10: 600 MHz 2D ^{1}H–^{13}C HMBC contour plot showing the region from ~4.0 to ~9.2 ppm in the ^{1}H dimension and from ~159 to ~187 ppm in the ^{13}C dimension for the chiral macrocycle in $CDCl_3$. The plot highlights the amide carbons (C_x and C_y) and their correlations with protons H_a,H_b, H_c, H_d, and H_e. The key correlations relevant for structural assignment include C_x–H_b, and H_e; and C_y–H_a, H_c, and H_d.

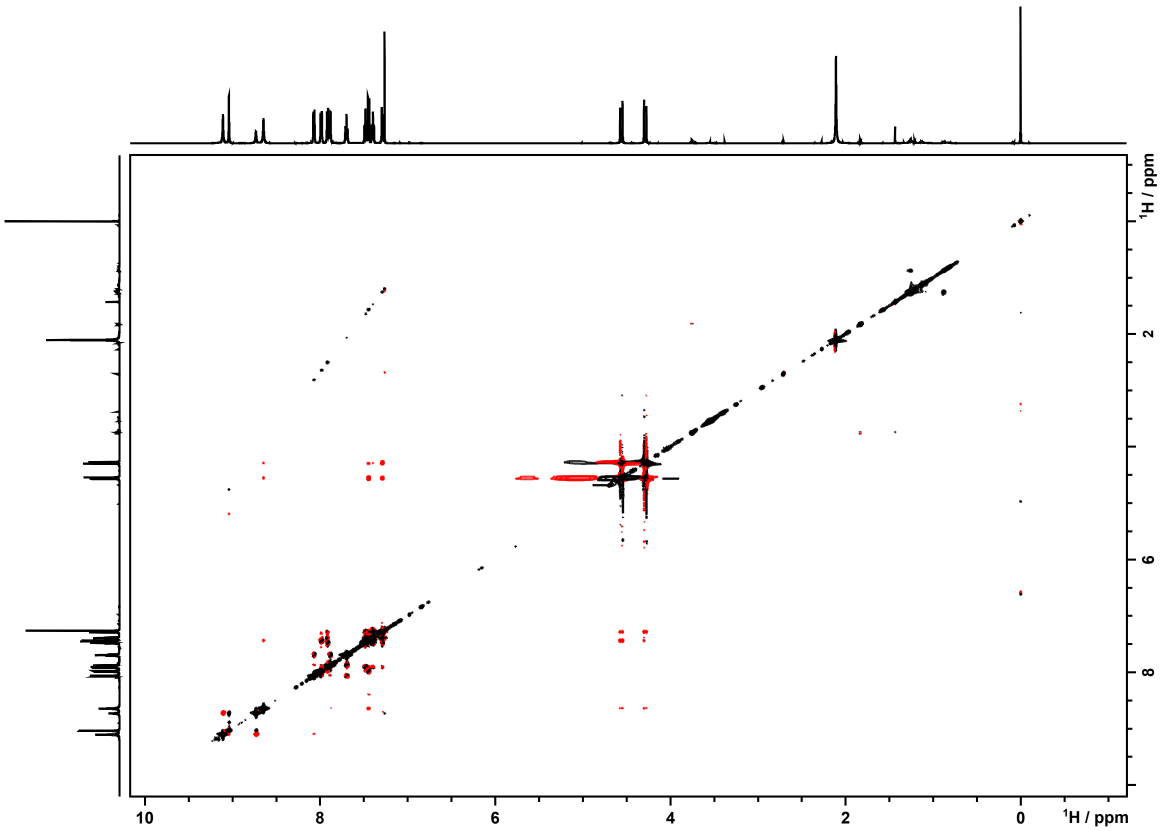

Figure S11: 600 MHz 2D ^{1}H–^{1}H ROESY contour map of the chiral macrocycle in $CDCl_3$.

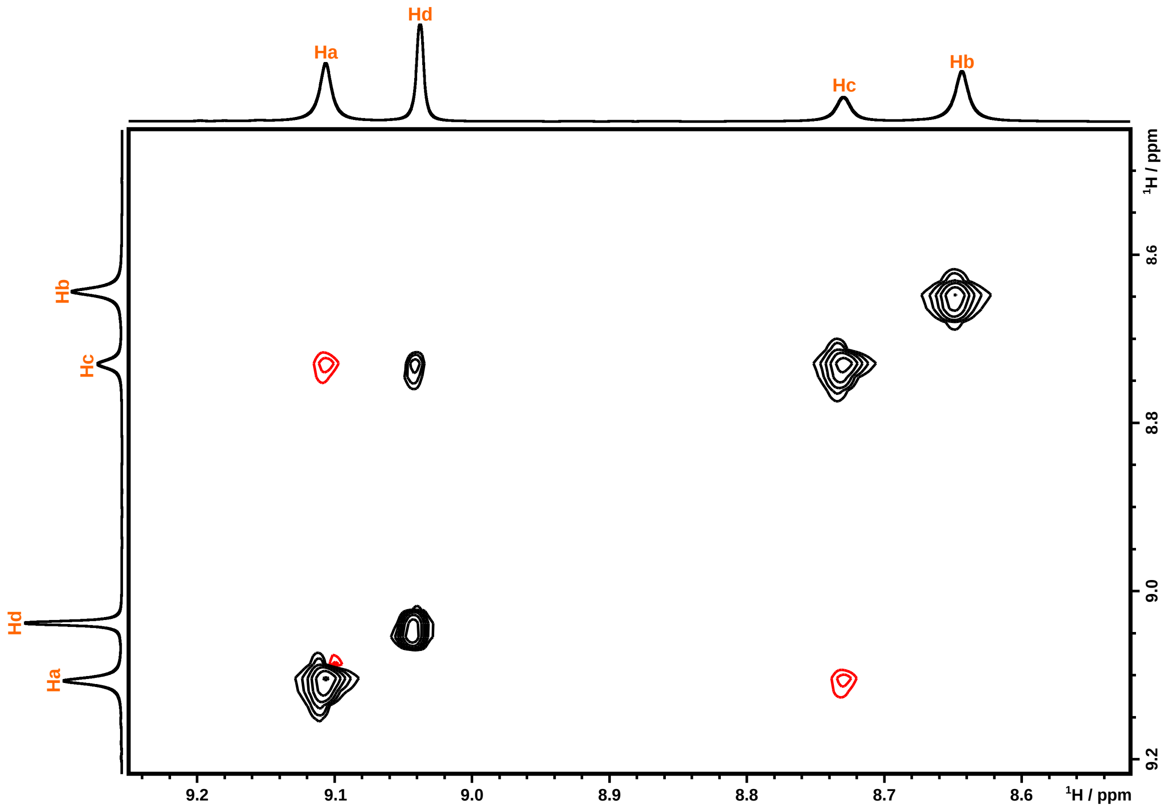

Figure S12: 600 MHz 2D ^{1}H–^{1}H ROESY contour plot showing the region from ~8.5 to ~9.3 ppm for the chiral macrocycle in $CDCl_3$. The red cross-peak in this region confirms the spatial proximity between protons H_a and H_c. The signals corresponding to proton H_a, H_b, H_c, and H_d of the macrocycle are highlighted.

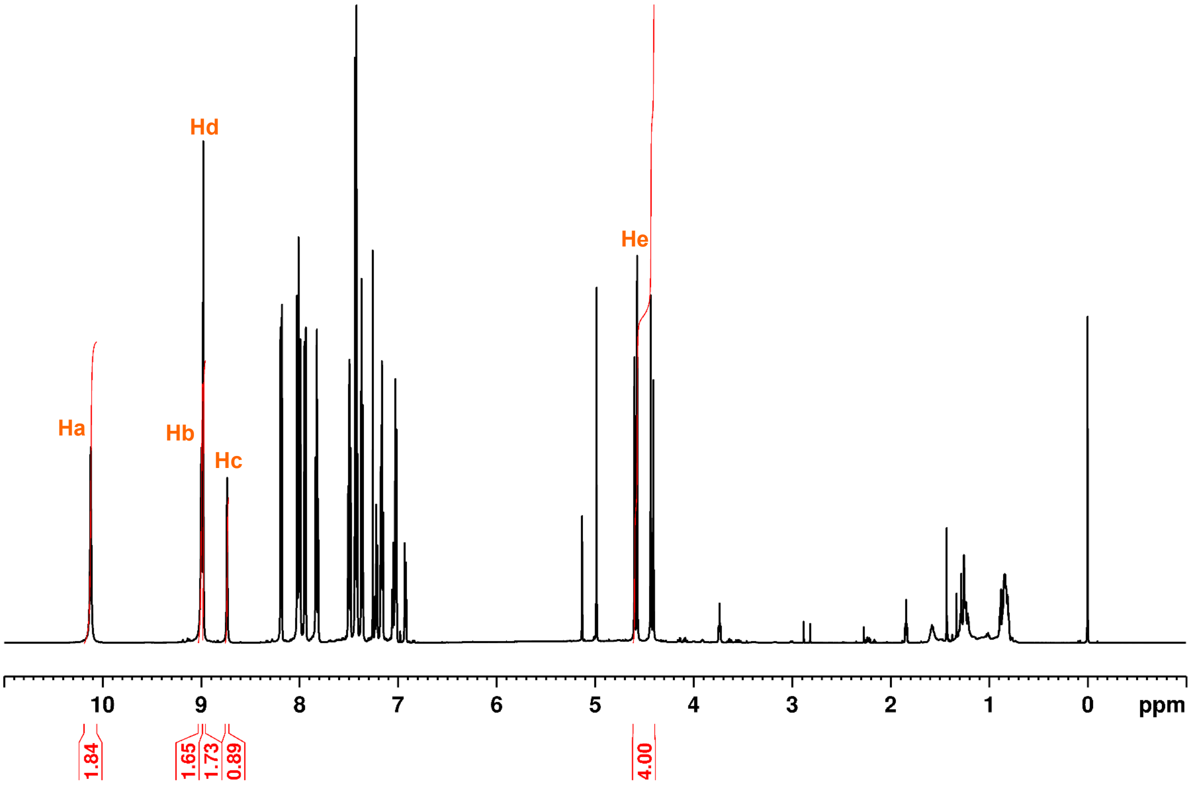

Figure S13: 600 MHz ^{1}H NMR spectrum of mandelic acid (MA) in the presence of the chiral macrocycle in $CDCl_3$. The enantiomeric composition was 70% (*R*)-MA and 30% (*S*)-MA. Resonances corresponding to macrocycle protons H_a, H_b, H_c, H_d, and H_e are highlighted.

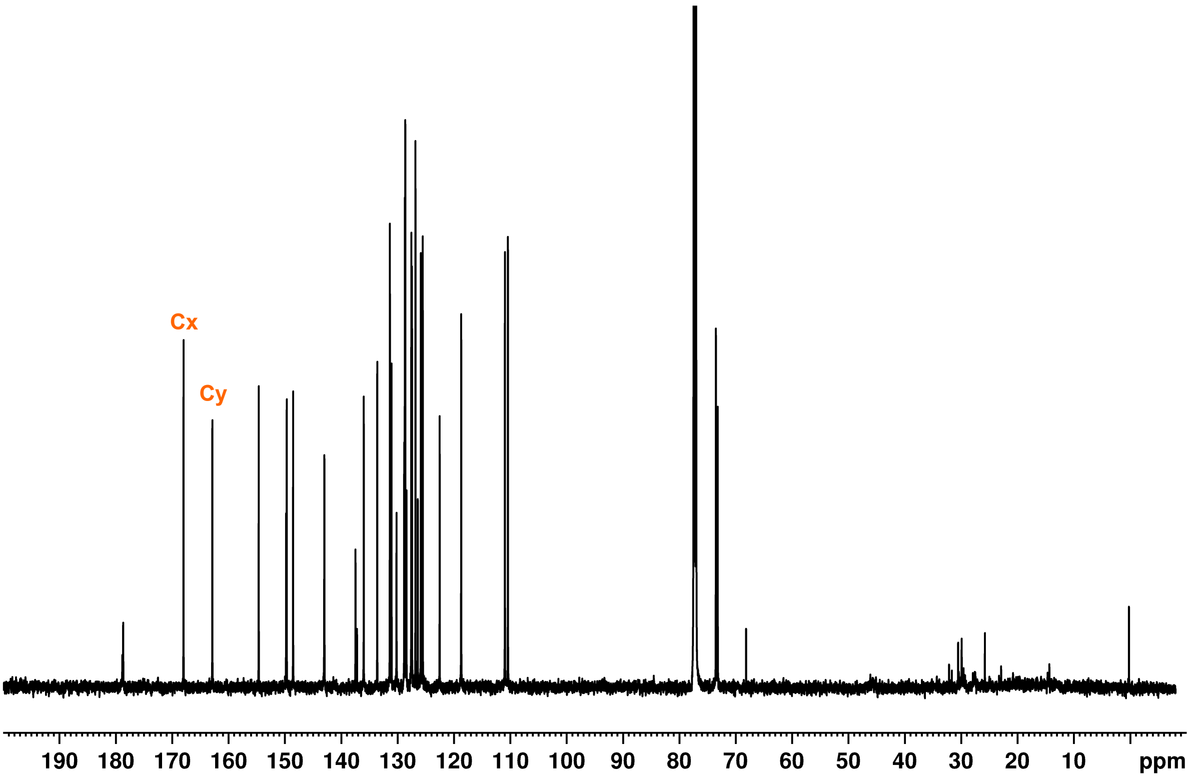

Figure S14: 150 MHz ^{13}C NMR spectrum of mandelic acid (MA) in the presence of the chiral macrocycle in $CDCl_3$. The enantiomeric composition was 70% (*R*)-MA and 30% (*S*)-MA. Resonances corresponding to macrocycle carbons C_x and C_y are highlighted.

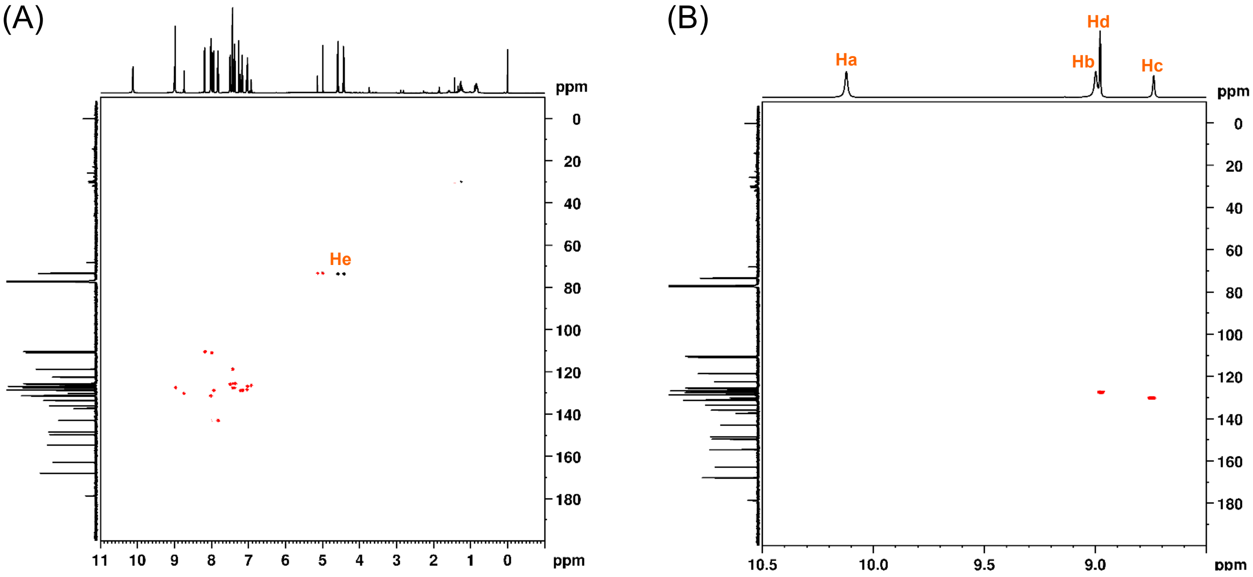

Figure S15: 600 MHz 2D ^{1}H–^{13}C multiplicity-edited HSQC contour map for mandelic acid (MA) in the presence of the chiral macrocycle in $CDCl_3$. The enantiomeric composition was 70% (*R*)-MA and 30% (*S*)-MA. **A)** Full HSQC, and **B)** Expanded view showing the region from 8.5 to 10.5 ppm in the ^{1}H dimension and from −10 to 200 ppm in the ^{13}C dimension. Resonances corresponding to macrocycle protons H_a, H_b, H_c, H_d, and H_e are highlighted. In the multiplicity-edited HSQC spectrum, red correlations correspond to CH_3 and CH groups, whereas black correlations correspond to CH_2 groups.

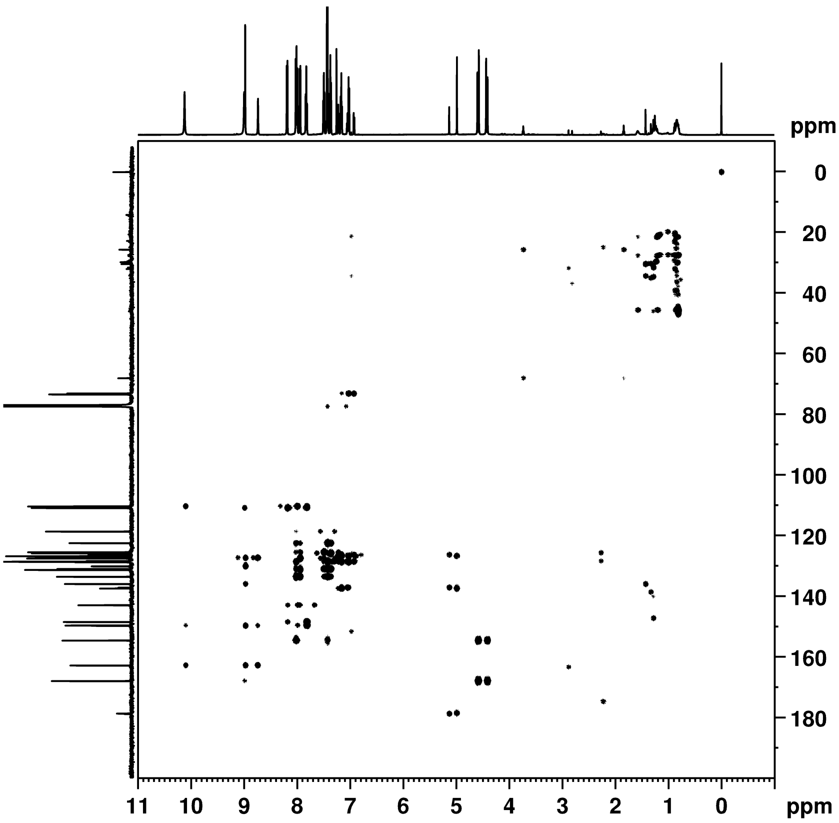

Figure S16: 600 MHz 2D ^{1}H–^{13}C HMBC contour map for mandelic acid (MA) in the presence of the chiral macrocycle in $CDCl_3$. The enantiomeric composition was 70% (*R*)-MA and 30% (*S*)-MA.

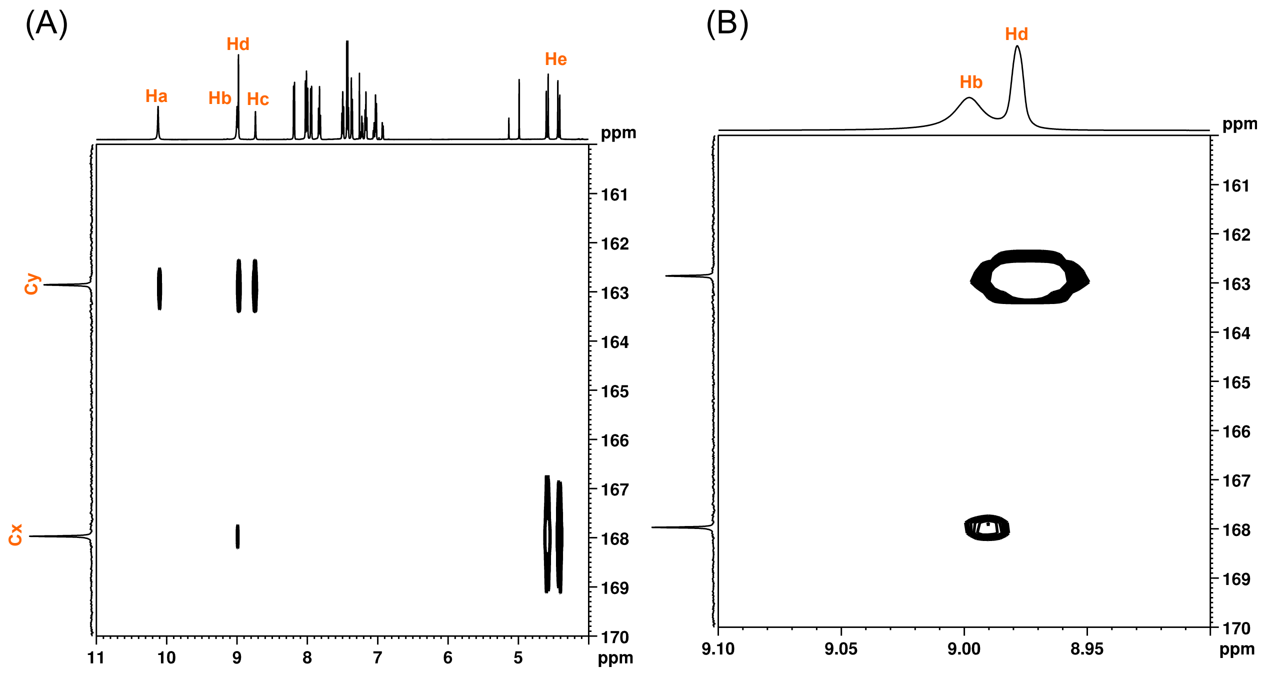

Figure S17: 600 MHz 2D ^{1}H–^{13}C HMBC contour map for mandelic acid (MA) in the presence of the chiral macrocycle in $CDCl_3$. The enantiomeric composition was 70% (*R*)-MA and 30% (*S*)-MA. Expanded view showing from 160 to 170 ppm in the ^{13}C dimension and the region from **A)** 4.0 to 11.0 ppm, and **B)** 8.9 to 9.1 ppm in the ^{1}H dimension. Resonances corresponding to macrocycle protons H_a, H_b, H_c, H_d, and H_e, and carbons C_x, and C_y are highlighted.

3.2 Experimental Diffusion Data

3.2.1 General Trends in the Experimental Enantiomeric Discrimination

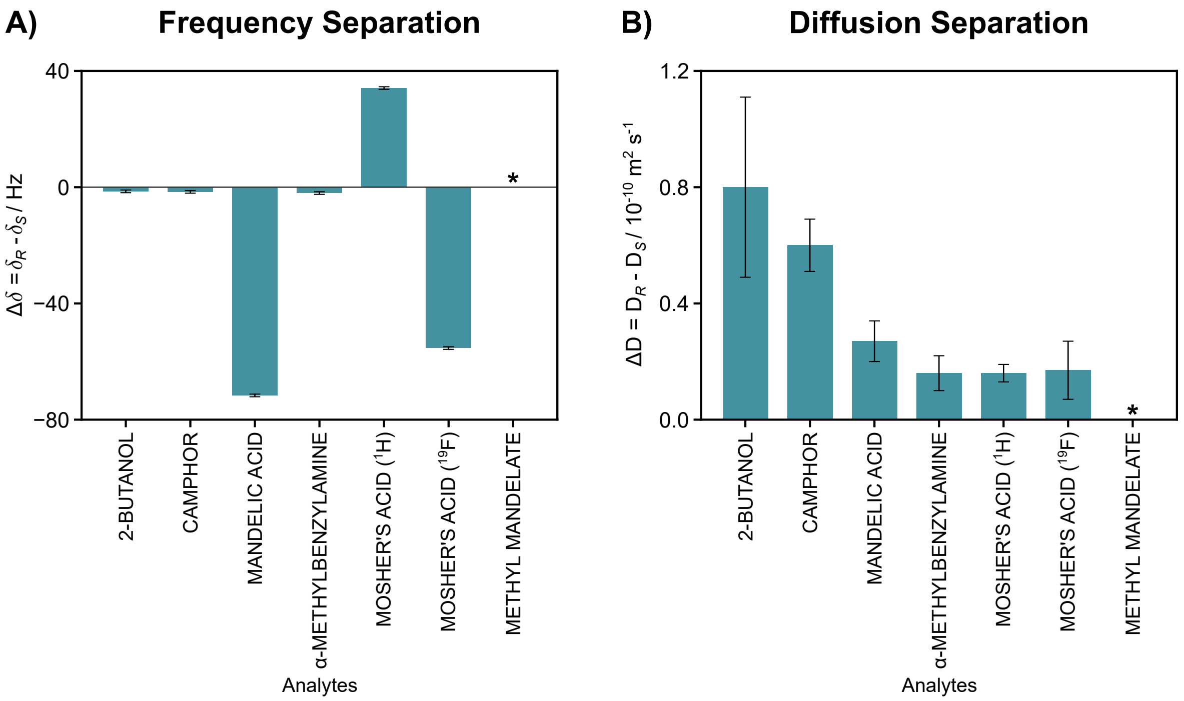

Figure S18: Comparison of the enantiomeric discrimination achieved with the chiral macrocycle for different analytes. **A)** Frequency separation, expressed as $\Delta\delta_{RS} = \delta_R - \delta_S$ (Hz), where δ_R and δ_S denote the chemical shifts of the (*R*)- and (*S*)-enantiomers, respectively. **B)** Diffusion separation, expressed as $\Delta D = D_R - D_S$ ($\times 10^{-10}$ m^2 s^{-1}), where D_R and D_S denote the diffusion coefficients of the (*R*)- and (*S*)-enantiomers, respectively. Error bars represent the uncertainties associated with the determination of $\Delta\delta_{RS}$ (FID resolution) and ΔD (propagated errors).

Table S1: Comparison of the frequency and diffusion discrimination achieved for different analytes in the presence of the chiral macrocycle. Frequency separations are reported as $\Delta\delta_{RS} = \delta_R - \delta_S$ in both Hz and ppm. Diffusion separations are reported as $\Delta D = D_R - D_S$ in units of 10^{-10} m^2 s^{-1}.

**Analyte**	$\Delta\delta_{RS}$ **(Hz)**	$\Delta\delta_{RS}$ **(ppm)**	ΔD	**Error**
Mandelic acid	-71.660	-0.143	0.27	0.07
2-Butanol	-1.420	-0.003	0.80	0.31
Camphor	-1.605	-0.003	0.60	0.09
α-Methylbenzylamine	-2.003	-0.004	0.16	0.06
Mosher's acid (^{1}H)	34.110	0.068	0.16	0.03
Mosher's acid (^{19}F)	-55.355	-0.111	0.17	0.10
Methyl mandelate	-	-	-	-

3.2.2 Mandelic Acid

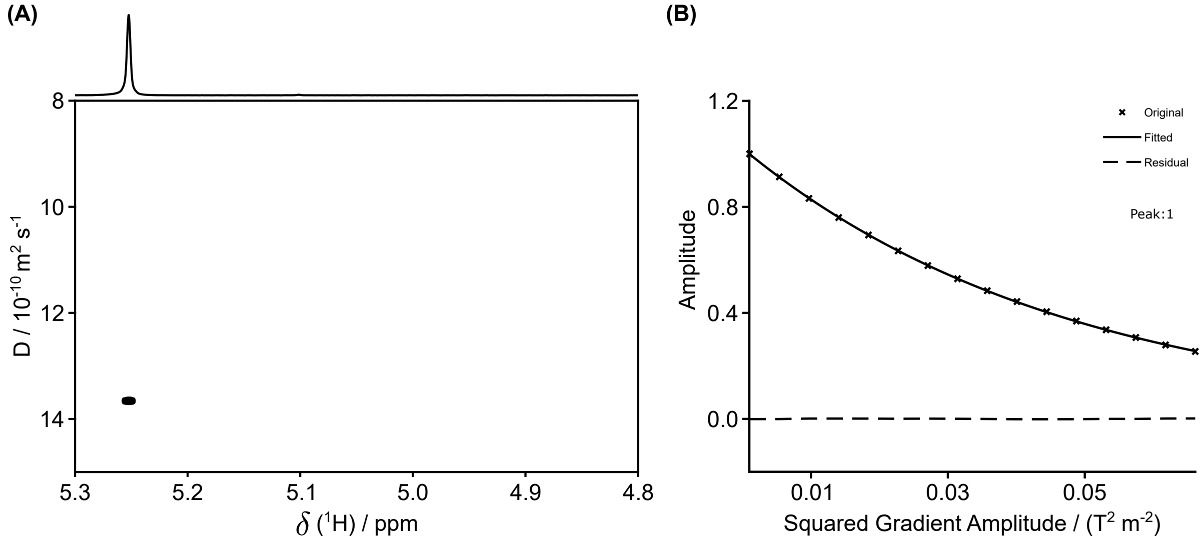

Figure S19: A) 500 MHz ^{1}H DOSY plot, with the least attenuated 1D spectrum shown at the top, and **B)** the corresponding signal fittings for an enantiomeric mixture of mandelic acid (MA) in the absence of a chiral macrocycle. The sample consists of 70% (*R*)-MA and 30% (*S*)-MA in $CDCl_3$. The DOSY data were processed using a Lorentzian window function with a line broadening of 0.3 Hz.

Table S2: ^{1}H DOSY (500 MHz) data for an enantiomeric mixture of mandelic acid (MA) consisting of 70% (*R*)-MA and 30% (*S*)-MA in $CDCl_3$. Chemical shift values are reported in ppm, and diffusion coefficients are expressed in units of 10^{-10} m^2 s^{-1}.

Compound	Frequency	Exp. Ampl.	Fit. Ampl.	Error	Diff. coef.	Error
(*R*/*S*)-MA	5.25229	0.82353	0.84072	0.00057	13.65866	0.01753

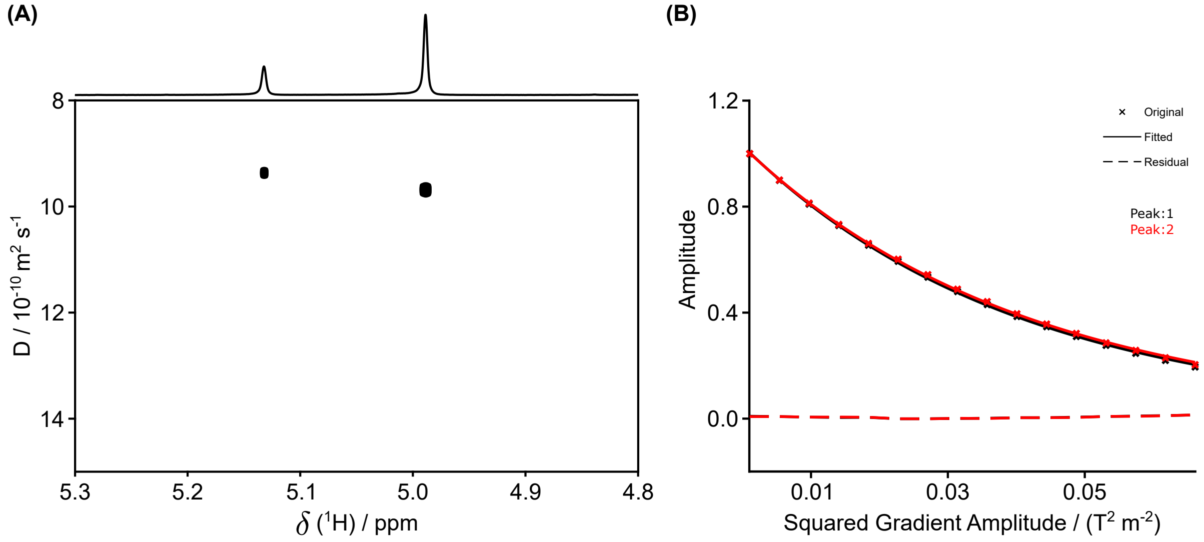

Figure S20: A) 500 MHz ^{1}H DOSY plot, with the least attenuated 1D spectrum shown at the top, and **B)** the corresponding signal fittings for an enantiomeric mixture of mandelic acid (MA) in the presence of the chiral macrocycle (MAC). The sample consists of 70% (*R*)-MA and 30% (*S*)-MA in $CDCl_3$. The DOSY data were processed using a Lorentzian window function with a line broadening of 0.3 Hz.

Table S3: ^{1}H DOSY (500 MHz) data for an enantiomeric mixture of mandelic acid (MA) consisting of 70% (*R*)-MA and 30% (*S*)-MA in the presence of the chiral macrocycle (MAC) in $CDCl_3$. Chemical shift values are reported in ppm, and diffusion coefficients are expressed in units of 10^{-10} m^2 s^{-1}.

Compound	Frequency	Exp. Ampl.	Fit. Ampl.	Error	Diff. coef.	Error
(*R*)-MA	4.98878	0.50763	0.52288	0.00152	9.66761	0.04964
(*S*)-MA	5.13210	0.18115	0.18620	0.00053	9.39724	0.04771

$$\Delta D_{RS} \pm \text{error}_{RS} = 0.27 \pm 0.07 \qquad (5)$$

The average diffusion coefficients and propagated errors presented here were calculated following the procedures described in the literature.[12,17]

3.2.3 2-butanol

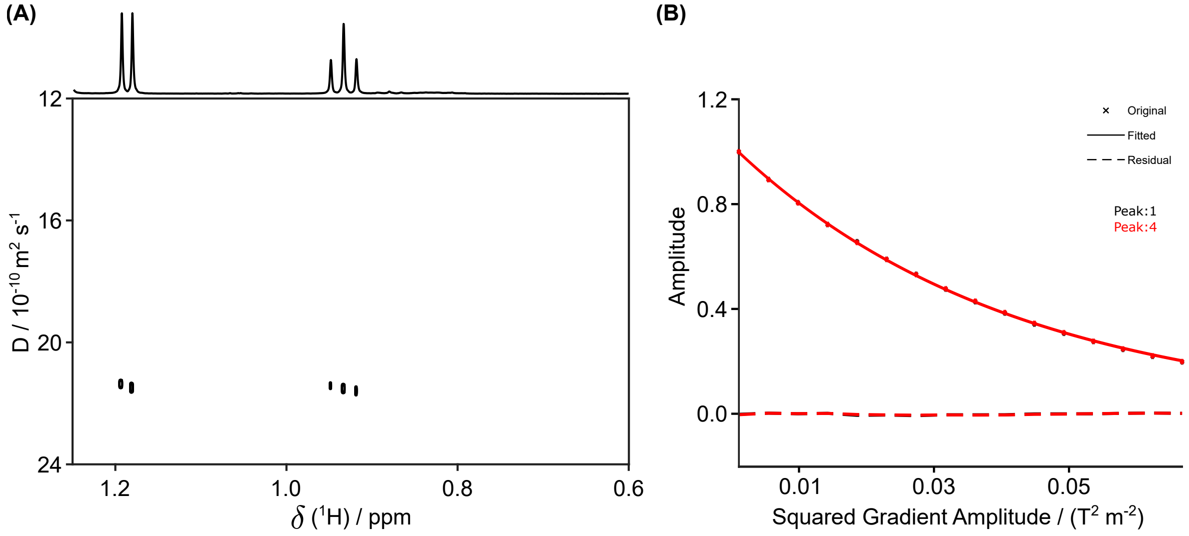

Figure S21: A) 500 MHz ^{1}H DOSY plot, with the least attenuated 1D spectrum shown at the top, and **B)** the corresponding signal fittings for an enantiomeric mixture of 2-butanol in the absence of a chiral macrocycle. The sample consists of 70% (*R*)-2-butanol and 30% (*S*)-2-butanol in $CDCl_3$. The DOSY data were processed using a Lorentzian window function with a line broadening of 0.5 Hz.

Table S4: ^{1}H DOSY (500 MHz) data for an enantiomeric mixture of 2-butanol consisting of 70% (*R*)-2-butanol and 30% (*S*)-2-butanol in $CDCl_3$. Chemical shift values are reported in ppm, and diffusion coefficients are expressed in units of 10^{-10} m^2 s^{-1}.

Compound	Frequency	Exp. Ampl.	Fit. Ampl.	Error	Diff. coef.	Error
(*R*/*S*)-2-butanol	0.91895	0.41894	0.42953	0.00093	21.59132	0.08308
(*R*/*S*)-2-butanol	0.93383	0.86773	0.88922	0.00165	21.51360	0.07089
(*R*/*S*)-2-butanol	0.94871	0.41904	0.42902	0.00066	21.42076	0.05880
(*R*/*S*)-2-butanol	1.18132	1.00000	1.02353	0.00199	21.48614	0.07405
(*R*/*S*)-2-butanol	1.19368	0.99926	1.02424	0.00167	21.36345	0.06177

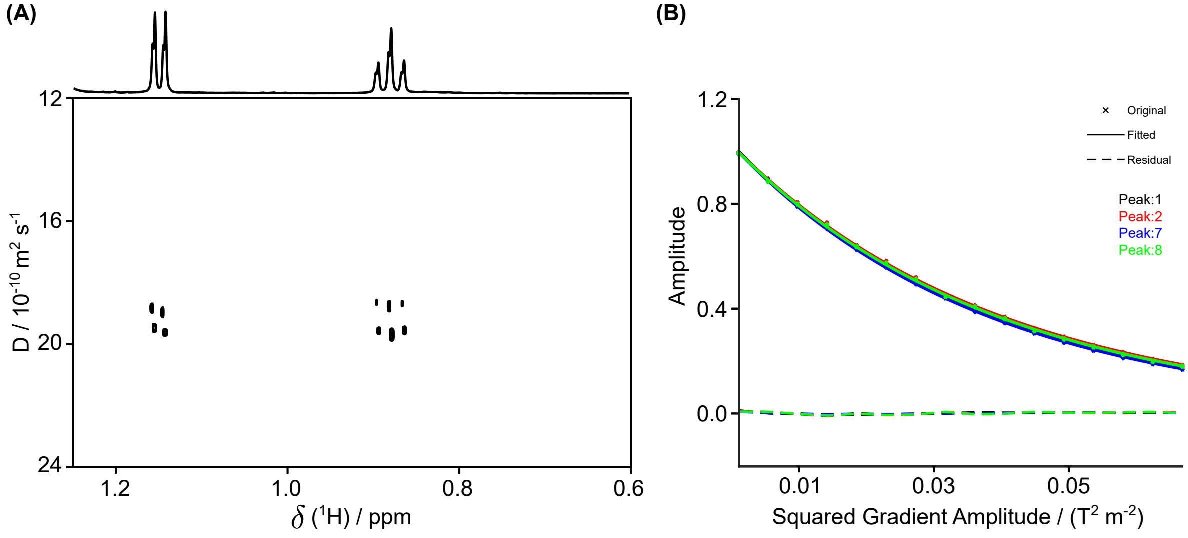

Figure S22: A) 500 MHz ^{1}H DOSY plot, with the least attenuated 1D spectrum shown at the top, and **B)** the corresponding signal fittings for an enantiomeric mixture of 2-butanol in the presence of the chiral macrocycle (MAC). The sample consists of 70% (*R*)-2-butanol and 30% (*S*)-2-butanol in $CDCl_3$. The DOSY data were processed using a Lorentzian window function with a line broadening of 0.5 Hz.

Table S5: ^{1}H DOSY (500 MHz) data for an enantiomeric mixture of 2-butanol consisting of 70% (*R*)-2-butanol and 30% (*S*)-2-butanol in the presence of the chiral macrocycle (MAC) in $CDCl_3$. Chemical shift values are reported in ppm, and diffusion coefficients are expressed in units of 10^{-10} m^2 s^{-1}.

Compound	Frequency	Exp. Ampl.	Fit. Ampl.	Error	Diff. coef.	Error
(*R*)-2-butanol	0.86365	0.40249	0.41806	0.00127	19.54908	0.10159
(*S*)-2-butanol	0.86663	0.25804	0.26689	0.00088	18.67689	0.10748
(*R*)-2-butanol	0.87876	0.78638	0.81809	0.00296	19.68875	0.12164
(*S*)-2-butanol	0.88174	0.50451	0.52153	0.00200	18.76349	0.12534
(*R*)-2-butanol	0.89364	0.37614	0.39022	0.00102	19.56419	0.08726
(*S*)-2-butanol	0.89662	0.25357	0.26113	0.00074	18.64175	0.09262
(*R*)-2-butanol	1.14273	1.00000	1.03350	0.00172	19.61829	0.05574
(*S*)-2-butanol	1.14548	0.58747	0.60705	0.00198	18.96331	0.10703
(*R*)-2-butanol	1.15533	0.96982	1.00122	0.00205	19.47135	0.06850
(*S*)-2-butanol	1.15784	0.60913	0.62679	0.00184	18.82715	0.09620

3.2.4 Camphor

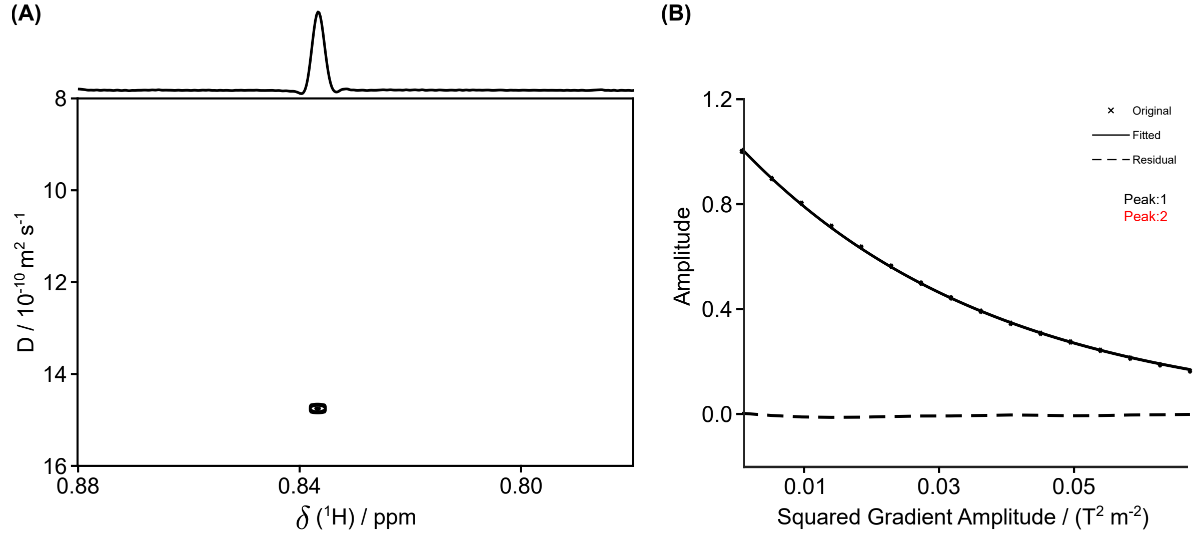

Figure S23: A) 500 MHz ^{1}H DOSY plot, with the least attenuated 1D spectrum shown at the top, and **B)** the corresponding signal fittings for an enantiomeric mixture of Camphor in the absence of a chiral macrocycle. The sample consists of 70% (*R*)-Camphor and 30% (*S*)-Camphor in $CDCl_3$. The DOSY data were processed using Lorentzian and Gaussian window functions with line broadening of -1.0 and 0.6 Hz, respectively.

Table S6: ^{1}H DOSY (500 MHz) data for an enantiomeric mixture of Camphor consisting of 70% (*R*)-Camphor and 30% (*S*)-Camphor in $CDCl_3$. Chemical shift values are reported in ppm, and diffusion coefficients are expressed in units of 10^{-10} m^2 s^{-1}.

Compound	Frequency	Exp. Ampl.	Fit. Ampl.	Error	Diff. coef.	Error
(*R*/*S*)-Camphor	0.83683	0.65394	0.67861	0.00189	14.75118	0.06995

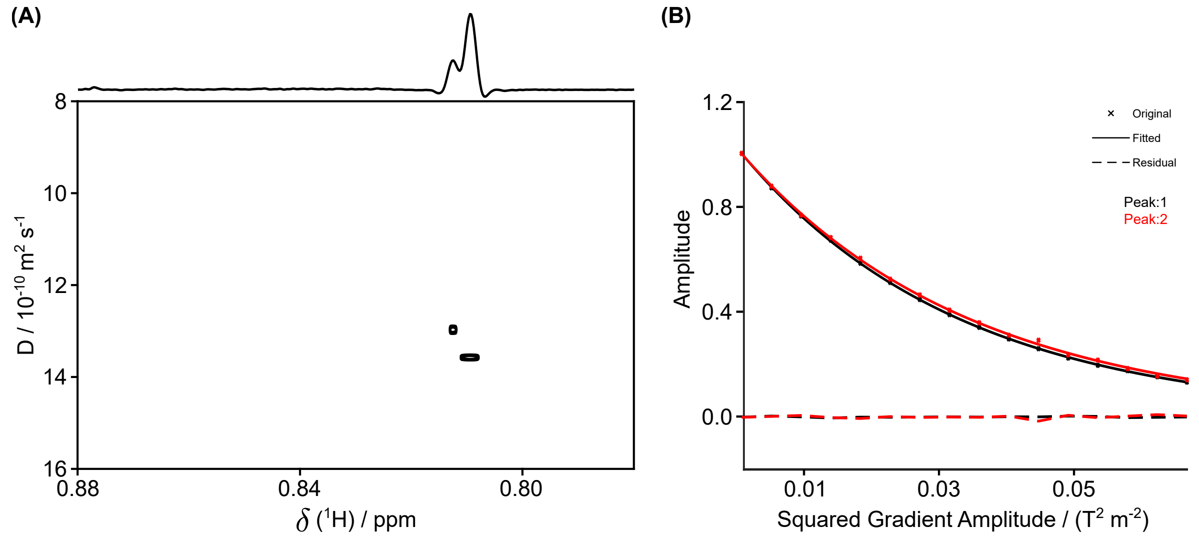

Figure S24: A) 500 MHz ^{1}H DOSY plot, with the least attenuated 1D spectrum shown at the top, and **B)** the corresponding signal fittings for an enantiomeric mixture of Camphor in the presence of the chiral macrocycle (MAC). The sample consists of 70% (*R*)-Camphor and 30% (*S*)-Camphor in $CDCl_3$. The DOSY data were processed using Lorentzian and Gaussian window functions with line broadening of -1.0 and 0.6 Hz, respectively.

Table S7: ^{1}H DOSY (500 MHz) data for an enantiomeric mixture of Camphor consisting of 70% (*R*)-Camphor and 30% (*S*)-Camphor in the presence of the chiral macrocycle (MAC) in $CDCl_3$. Chemical shift values are reported in ppm, and diffusion coefficients are expressed in units of 10^{-10} m^2 s^{-1}.

Compound	Frequency	Exp. Ampl.	Fit. Ampl.	Error	Diff. coef.	Error
(*R*)-Camphor	0.80949	0.36053	0.37200	0.00052	13.57482	0.03114
(*S*)-Camphor	0.81270	0.13838	0.14252	0.00059	12.97207	0.08853

3.2.5 Methylbenzylamine

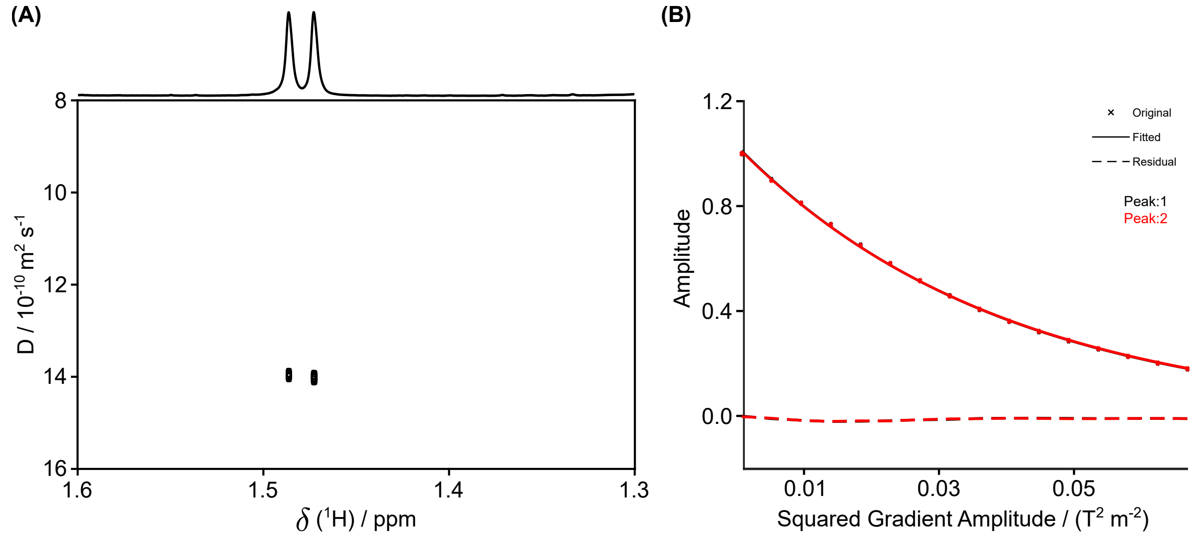

Figure S25: A) 500 MHz ^{1}H DOSY plot, with the least attenuated 1D spectrum shown at the top, and **B)** the corresponding signal fittings for an enantiomeric mixture of Methylbenzylamine in the absence of a chiral macrocycle. The sample consists of 70% (*R*)-Methylbenzylamine and 30% (*S*)-Methylbenzylamine in $CDCl_3$. The DOSY data were processed using a Lorentzian window function with a line broadening of 0.5 Hz.

Table S8: ^{1}H DOSY (500 MHz) data for an enantiomeric mixture of Methylbenzylamine consisting of 70% (*R*)-Methylbenzylamine and 30% (*S*)-Methylbenzylamine in $CDCl_3$. Chemical shift values are reported in ppm, and diffusion coefficients are expressed in units of 10^{-10} m^2 s^{-1}.

Compound	Frequency	Exp. Ampl.	Fit. Ampl.	Error	Diff. coef.	Error
(*R*/*S*)-α-Methylbenzylamine	1.47300	1.00000	1.03863	0.00367	14.01740	0.08614
(*R*/*S*)-α-Methylbenzylamine	1.48651	0.99897	1.03525	0.00330	13.96234	0.07753

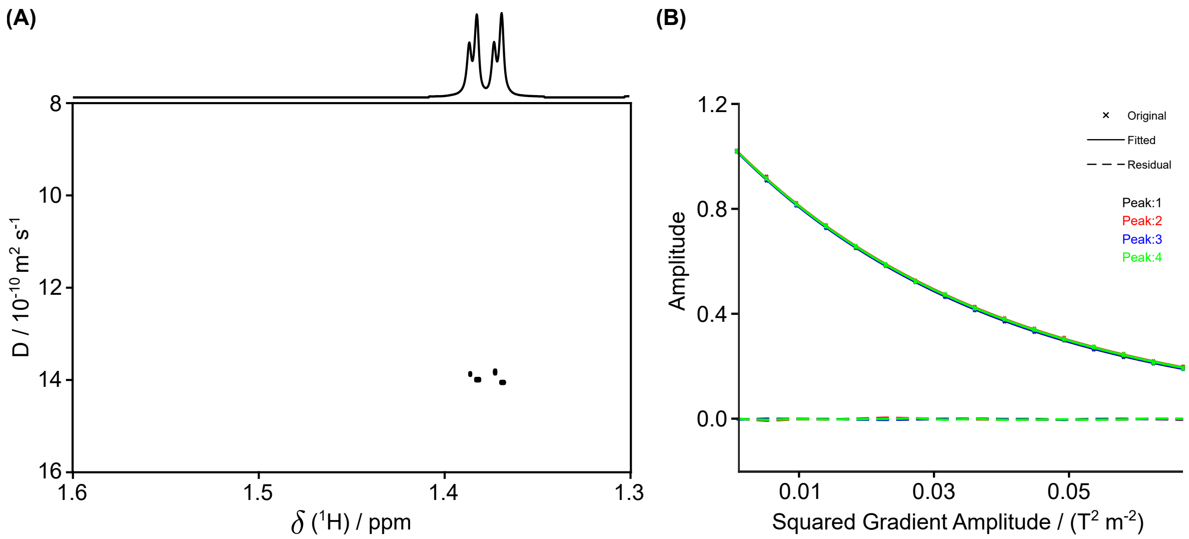

Figure S26: A) 500 MHz ^{1}H DOSY plot, with the least attenuated 1D spectrum shown at the top, and **B)** the corresponding signal fittings for an enantiomeric mixture of Methylbenzylamine in the presence of the chiral macrocycle (MAC). The sample consists of 70% (*R*)-Methylbenzylamine and 30% (*S*)-Methylbenzylamine in $CDCl_3$. The DOSY data were processed using a Lorentzian window function with a line broadening of 0.5 Hz.

Table S9: ^{1}H DOSY (500 MHz) data for an enantiomeric mixture of Methylbenzylamine consisting of 70% (*R*)-Methylbenzylamine and 30% (*S*)-Methylbenzylamine in the presence of the chiral macrocycle (MAC) in $CDCl_3$. Chemical shift values are reported in ppm, and diffusion coefficients are expressed in units of 10^{-10} m^2 s^{-1}.

Compound	Frequency	Exp. Ampl.	Fit. Ampl.	Error	Diff. coef.	Error
(*R*)-α-Methylbenzylamine	1.36895	0.98856	1.01545	0.00079	14.05307	0.01904
(*S*)-α-Methylbenzylamine	1.37284	0.65814	0.67699	0.00116	13.82735	0.04130
(*R*)-α-Methylbenzylamine	1.38223	0.98459	1.00930	0.00090	13.97504	0.02181
(*S*)-α-Methylbenzylamine	1.38635	0.64727	0.66504	0.00078	13.87151	0.02830

3.2.6 Mosher's Acid

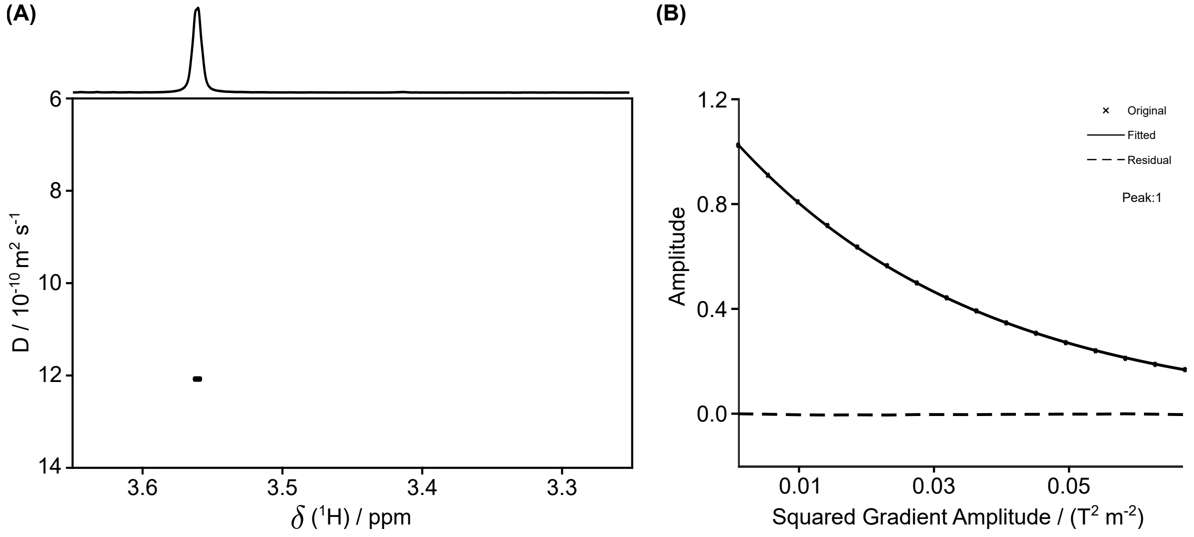

Figure S27: A) 500 MHz ^{1}H DOSY plot, with the least attenuated 1D spectrum shown at the top, and **B)** the corresponding signal fittings for an enantiomeric mixture of Mosher's Acid in the absence of a chiral macrocycle. The sample consists of 70% (*R*)-Mosher's Acid and 30% (*S*)-Mosher's Acid in $CDCl_3$. The DOSY data were processed using a Lorentzian window function with a line broadening of 1.0 Hz.

Table S10: ^{1}H DOSY (500 MHz) data for an enantiomeric mixture of Mosher's Acid consisting of 70% (*R*)-Mosher's Acid and 30% (*S*)-Mosher's Acid in $CDCl_3$. Chemical shift values are reported in ppm, and diffusion coefficients are expressed in units of 10^{-10} m^2 s^{-1}.

Compound	Frequency	Exp. Ampl.	Fit. Ampl.	Error	Diff. coef.	Error
(*R*/*S*)-Mosher's acid	3.56032	0.99761	1.02910	0.00103	12.07674	0.02046

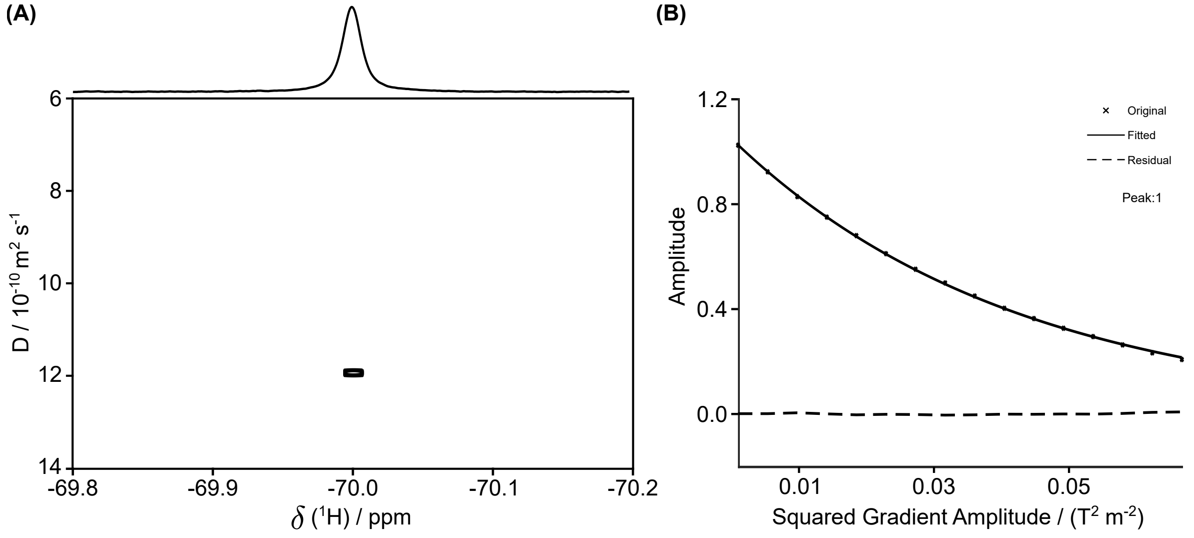

Figure S28: A) 470 MHz ^{19}F DOSY plot, with the least attenuated 1D spectrum shown at the top, and **B)** the corresponding signal fittings for an enantiomeric mixture of Mosher's Acid in the absence of a chiral macrocycle. The sample consists of 70% (*R*)-Mosher's Acid and 30% (*S*)-Mosher's Acid in $CDCl_3$. The DOSY data were processed using a Lorentzian window function with a line broadening of 1.0 Hz.

Table S11: ^{19}F DOSY (470 MHz) data for an enantiomeric mixture of Mosher's Acid consisting of 70% (*R*)-Mosher's Acid and 30% (*S*)-Mosher's Acid in $CDCl_3$. Chemical shift values are reported in ppm, and diffusion coefficients are expressed in units of 10^{-10} m^2 s^{-1}.

Compound	Frequency	Exp. Ampl.	Fit. Ampl.	Error	Diff. coef.	Error
(*R*/*S*)-Mosher's acid	-70.00034	1.00000	1.02606	0.00231	11.92684	0.04794

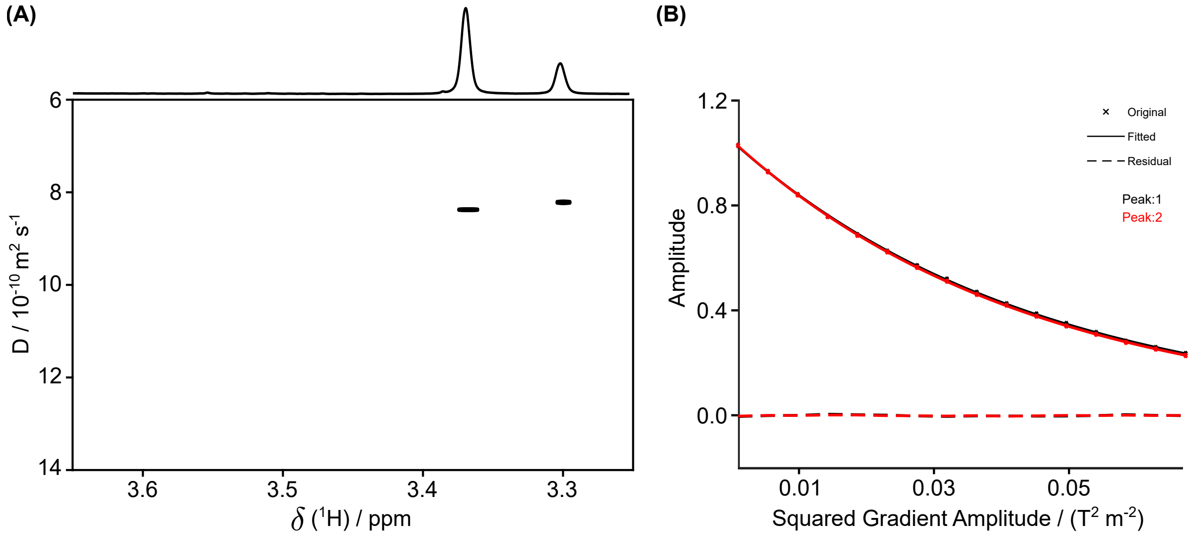

Figure S29: A) 500 MHz ^{1}H DOSY plot, with the least attenuated 1D spectrum shown at the top, and **B)** the corresponding signal fittings for an enantiomeric mixture of Mosher's Acid in the presence of the chiral macrocycle (MAC). The sample consists of 70% (*R*)-Mosher's Acid and 30% (*S*)-Mosher's Acid in $CDCl_3$. The DOSY data were processed using a Lorentzian window function with a line broadening of 1.0 Hz.

Table S12: ^{1}H DOSY (500 MHz) data for an enantiomeric mixture of Mosher's Acid consisting of 70% (*R*)-Mosher's Acid and 30% (*S*)-Mosher's Acid in the presence of the chiral macrocycle (MAC) in $CDCl_3$. Chemical shift values are reported in ppm, and diffusion coefficients are expressed in units of 10^{-10} m^2 s^{-1}.

Compound	Frequency	Exp. Ampl.	Fit. Ampl.	Error	Diff. coef.	Error
(*S*)-Mosher's acid	3.29966	0.35695	0.36405	0.00062	8.21627	0.02571
(*R*)-Mosher's acid	3.36788	0.99779	1.01996	0.00090	8.37573	0.01339

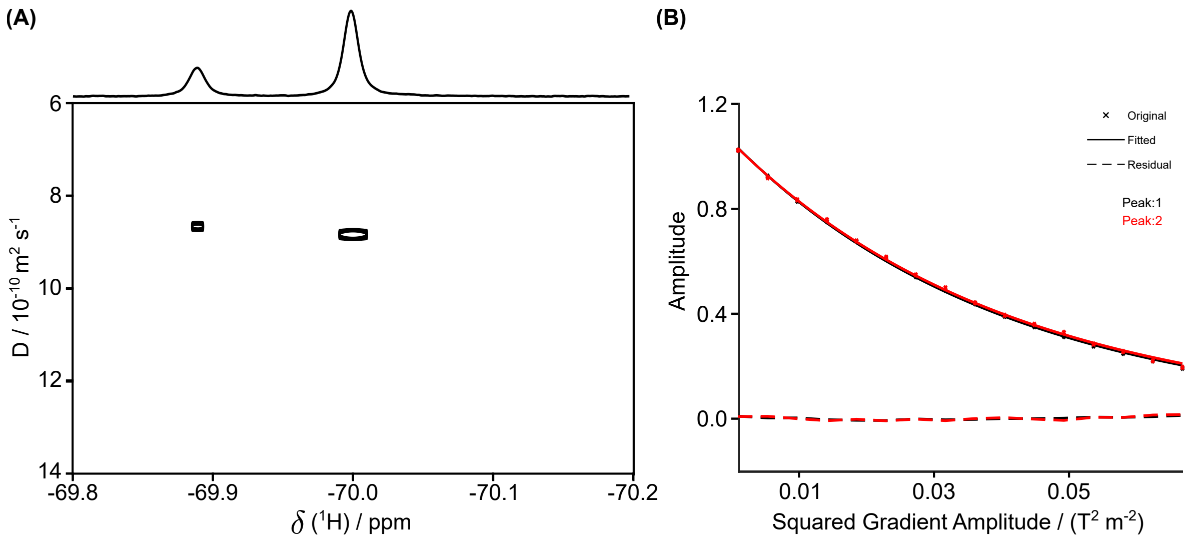

Figure S30: A) 470 MHz ^{19}F DOSY plot, with the least attenuated 1D spectrum shown at the top, and **B)** the corresponding signal fittings for an enantiomeric mixture of Mosher's Acid in the presence of the chiral macrocycle (MAC). The sample consists of 70% (*R*)-Mosher's Acid and 30% (*S*)-Mosher's Acid in $CDCl_3$. The DOSY data were processed using a Lorentzian window function with a line broadening of 1.0 Hz.

Table S13: ^{19}F DOSY (470 MHz) data for an enantiomeric mixture of Mosher's Acid consisting of 70% (*R*)-Mosher's Acid and 30% (*S*)-Mosher's Acid in the presence of the chiral macrocycle (MAC) in $CDCl_3$. Chemical shift values are reported in ppm, and diffusion coefficients are expressed in units of 10^{-10} m^2 s^{-1}.

Compound	Frequency	Exp. Ampl.	Fit. Ampl.	Error	Diff. coef.	Error
(*R*)-Mosher's acid	-69.99992	1.00000	1.03489	0.00392	8.83204	0.05880
(*S*)-Mosher's acid	-69.88921	0.33434	0.34531	0.00173	8.65902	0.07686

3.2.7 Methyl Mandelate

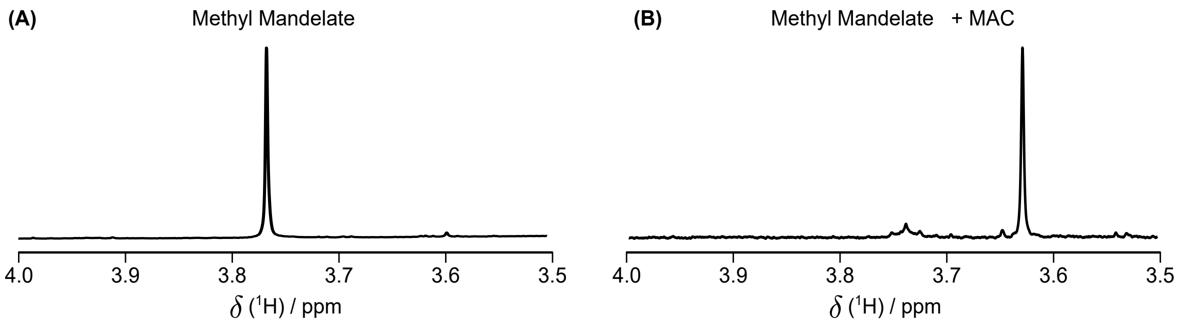

Figure S31: A) 500 MHz ^{1}H NMR spectral region between 3.5 and 4.0 ppm showing the resonance corresponding to the methoxy group of an enantiomeric mixture of methyl mandelate in the absence of the chiral macrocycle (MAC), and **B)** the corresponding spectral region in the presence of the resolving agent. The sample consists of 70% (*R*)-methyl mandelate and 30% (*S*)-methyl mandelate in $CDCl_3$. No enantiomeric discrimination was observed under these conditions. The ^{1}H data were processed using a Lorentzian window function with a line broadening of 0.5 Hz.

3.3 Temperature Variation Measurements

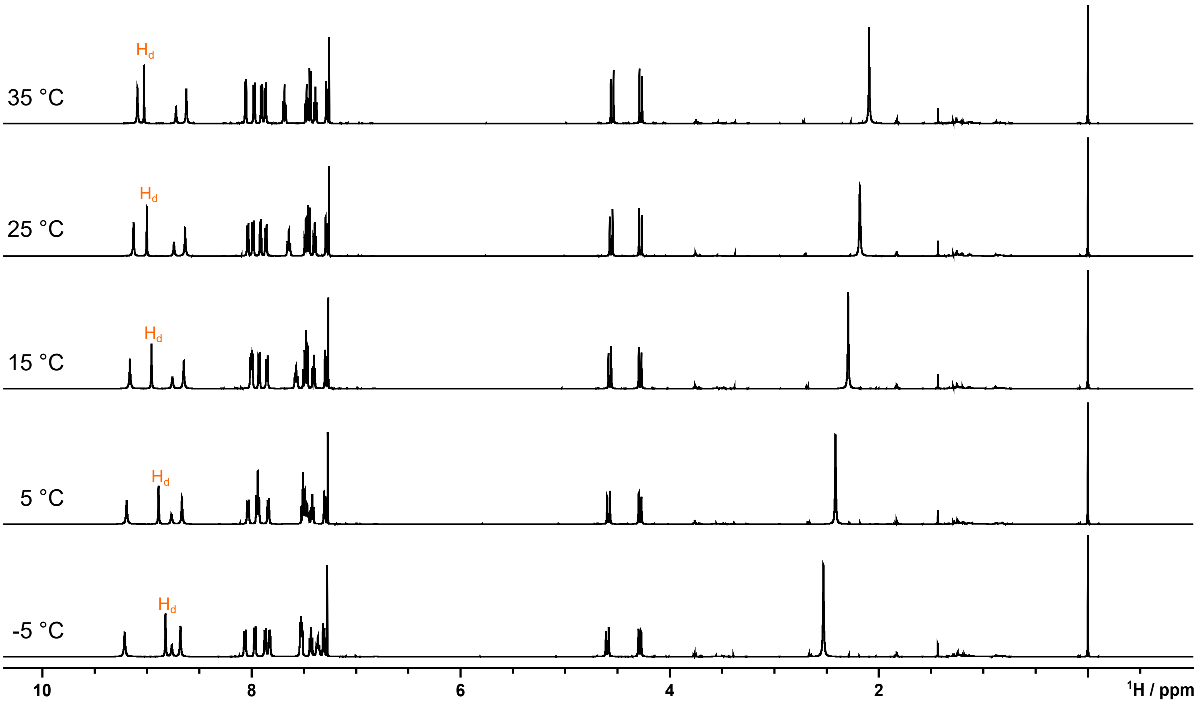

Figure S32: 600 MHz ^{1}H NMR spectra of the chiral macrocycle in $CDCl_3$ acquired over a temperature range from $-5\,°C$ to $35\,°C$. The signal corresponding to proton H_d of the macrocycle is highlighted.

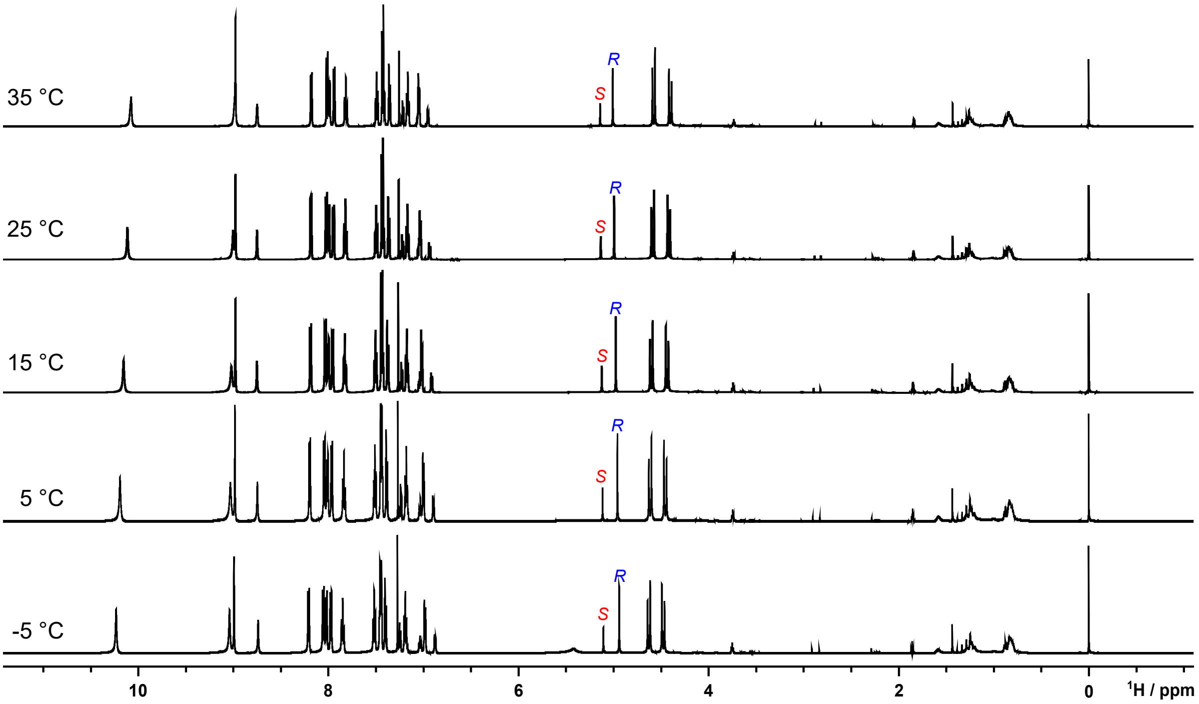

Figure S33: 600 MHz ^{1}H NMR spectra of the enantiomeric mixture (70 % *R* + 30% *S*) of mandelic acid in the presence of the chiral macrocycle in $CDCl_3$ acquired over a temperature range from $-5\,°C$ to $35\,°C$. The signals corresponding to the proton at the chiral center of (*R*)-enantiomer and (*S*)-enantiomer are highlighted.

Table S14: Difference between the ^{1}H chemical shifts of the (R)- and (S)-enantiomers, expressed as $\Delta\delta_{RS} = \delta_R - \delta_S$ (in Hz), for an enantiomeric mixture of mandelic acid in the presence of the chiral macrocycle in $CDCl_3$. Measurements were acquired at various temperatures (in °C) on a 600 MHz spectrometer. The sample contains 70% (R)-enantiomer and 30% (S)-enantiomer.

**Temperature** (°C)	$\Delta\delta_{RS}$ **(Hz)**
35	−80.2
25	−84.1
15	−88.6
5	−93.9
−5	−99.8

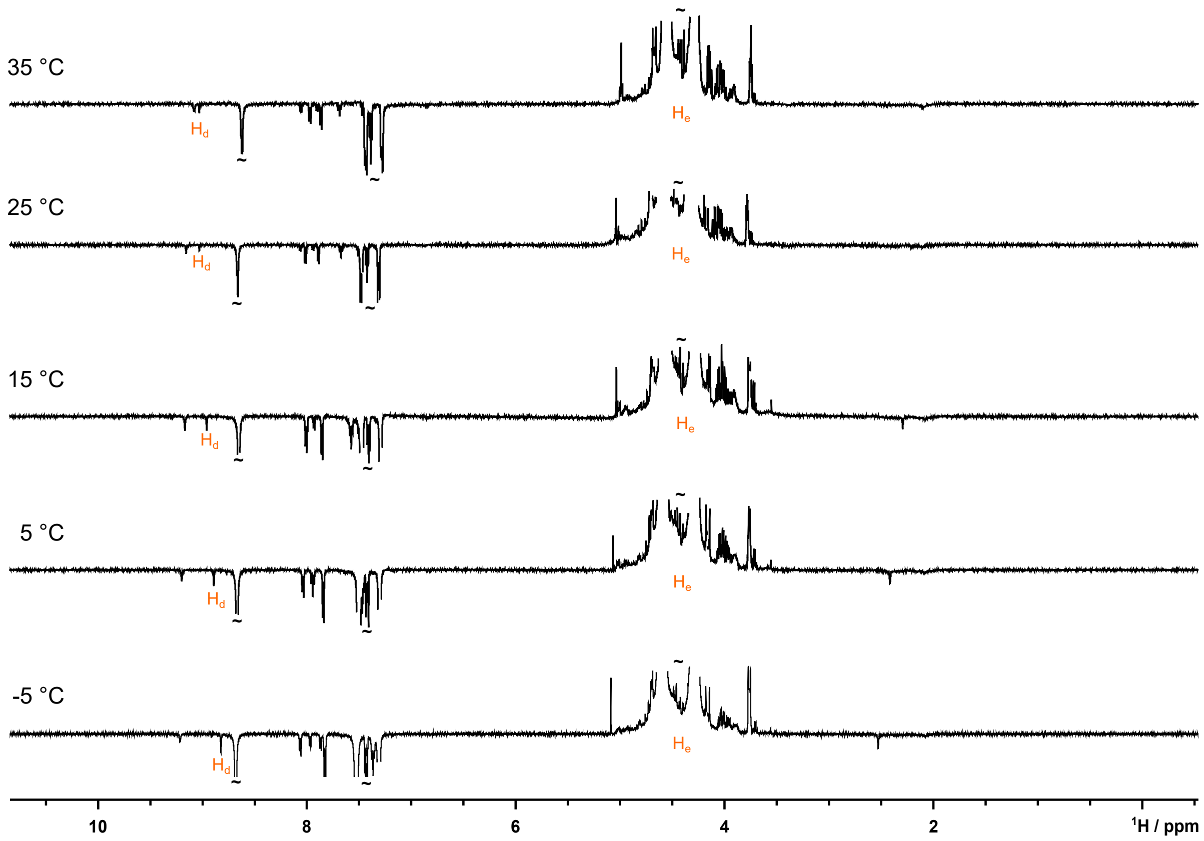

Figure S34: 600 MHz 1D ^{1}H selective ROESY spectra of the chiral macrocycle in $CDCl_3$ acquired across the temperature range −5 °C to 35 °C. In the ROESY spectra, the inverted cross-relaxation signals report on spatial interactions between the macrocycle protons and the selectively excited H_e. The resonances corresponding to protons H_d and H_e are highlighted.

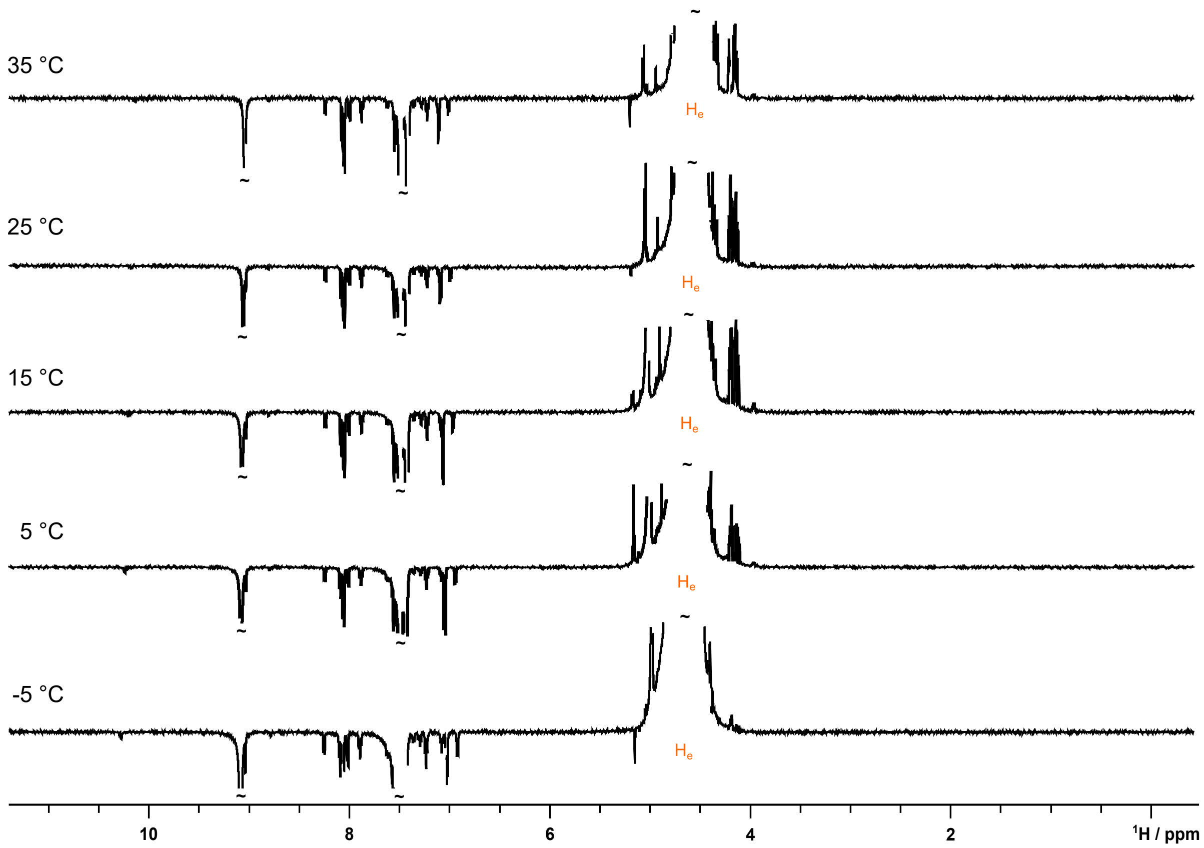

Figure S35: 600 MHz 1D ^{1}H selective ROESY spectra of the enantiomeric mixture (70 % *R* + 30% *S*) of mandelic acid in the presence of the chiral macrocycle in $CDCl_3$ acquired across the temperature range −5 °C to 35 °C. In the ROESY spectra, the inverted cross-relaxation signals report on spatial interactions between the macrocycle protons and the selectively excited H_e. The resonances corresponding to protons H_e are highlighted.

3.4 Additional Selective ROESY Experiments

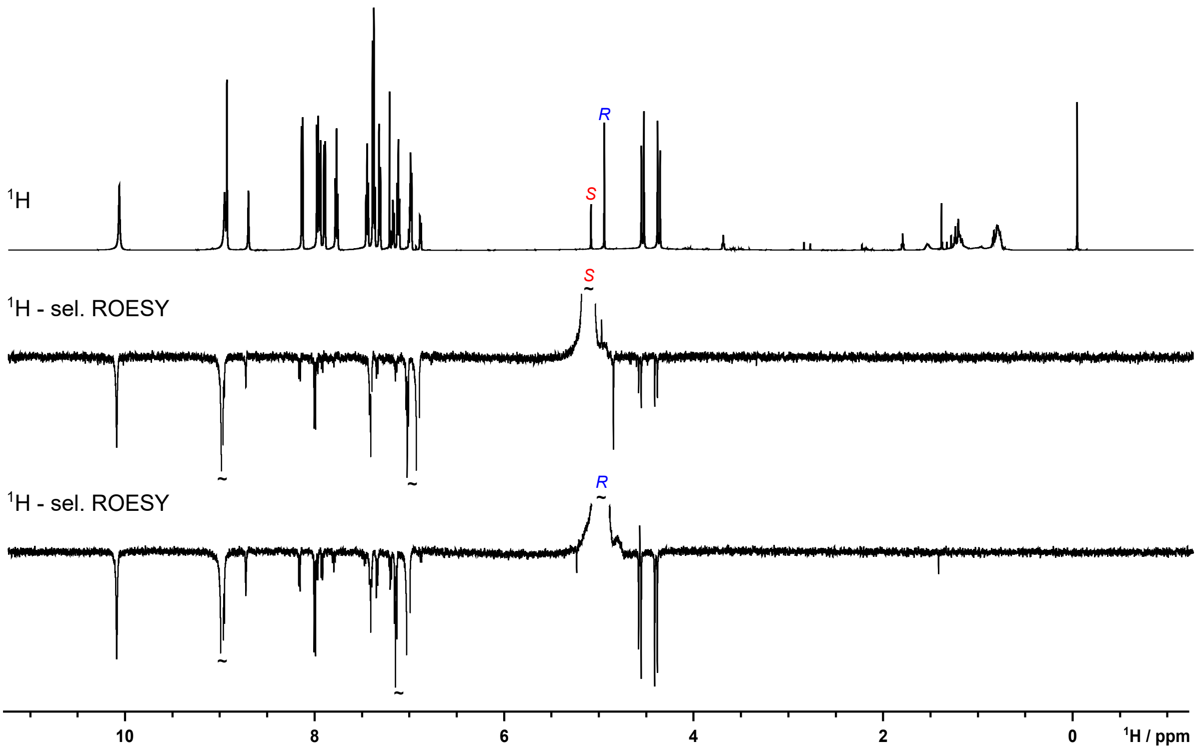

Figure S36: 600 MHz ^{1}H NMR spectrum of the enantiomeric mixture of mandelic acid (70% *R* + 30% *S*) in the presence of the chiral macrocycle in $CDCl_3$ (top). Shown below are the 600 MHz 1D ^{1}H selective ROESY spectra of the same mixture, with selective excitation of the proton at the chiral center of the (*S*)-enantiomer (middle) and the (*R*)-enantiomer (bottom). In the ROESY spectra, the inverted cross-relaxation responses indicate through-space interactions between the protons at the chiral center of mandelic acid and the protons of the macrocycle. The resonances corresponding to the excited protons of the (*R*)- and (*S*)-enantiomers are highlighted.

3.5 Variable-Temperature 2D ^1H-^1H EXSY Experiments

3.5.1 EXSY Experiments at 50.0 °C

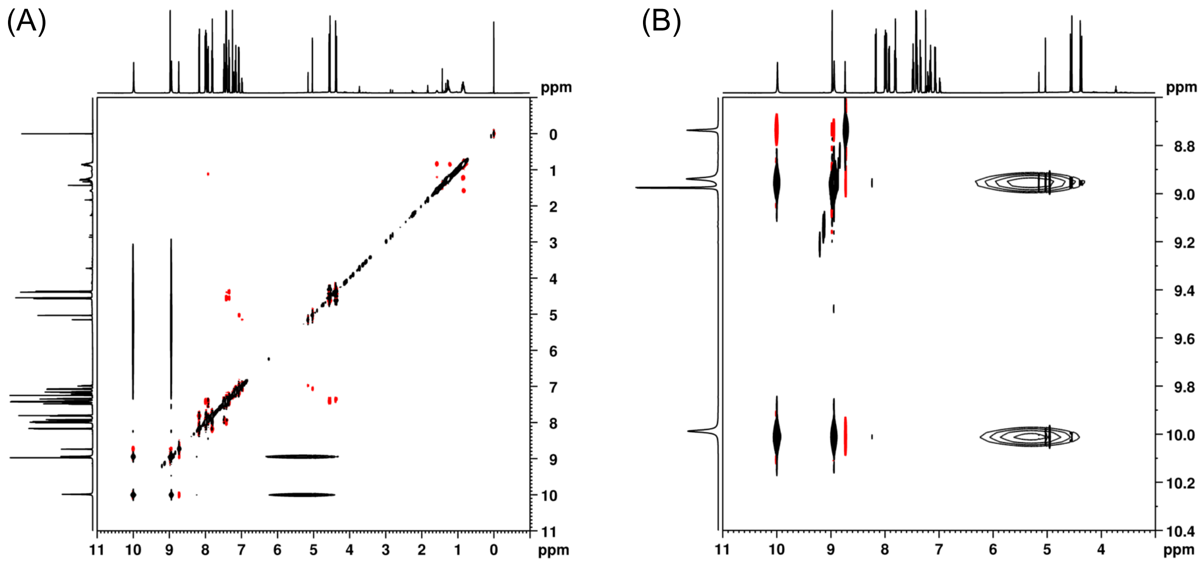

Figure S37: 600 MHz ^1H–^1H EXSY contour maps for an enantiomeric mixture of mandelic acid (MA), containing 70% (*R*)-MA, in the presence of the chiral macrocycle in $CDCl_3$. The spectrum was recorded at 50.0 °C with a mixing time of 500 ms. **A)** Full spectral region and **B)** expanded view of the 3.0–11.0 ppm and 8.6–10.4 ppm regions. Exchange cross-peaks are highlighted in black, whereas NOE cross-peaks are shown in red.

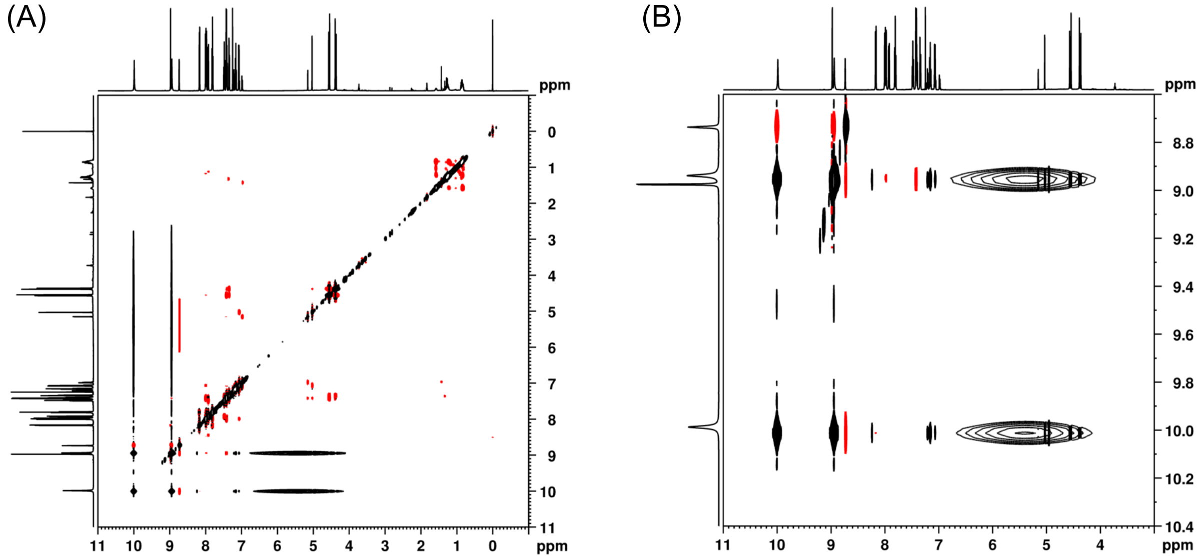

Figure S38: 600 MHz ^1H–^1H EXSY contour maps for an enantiomeric mixture of mandelic acid (MA), containing 70% (*R*)-MA, in the presence of the chiral macrocycle in $CDCl_3$. The spectrum was recorded at 50.0 °C with a mixing time of 1000 ms. **A)** Full spectral region and **B)** expanded view of the 3.0–11.0 ppm and 8.6–10.4 ppm regions. Exchange cross-peaks are highlighted in black, whereas NOE cross-peaks are shown in red. The contour plot intensity was divided by a factor of 8.

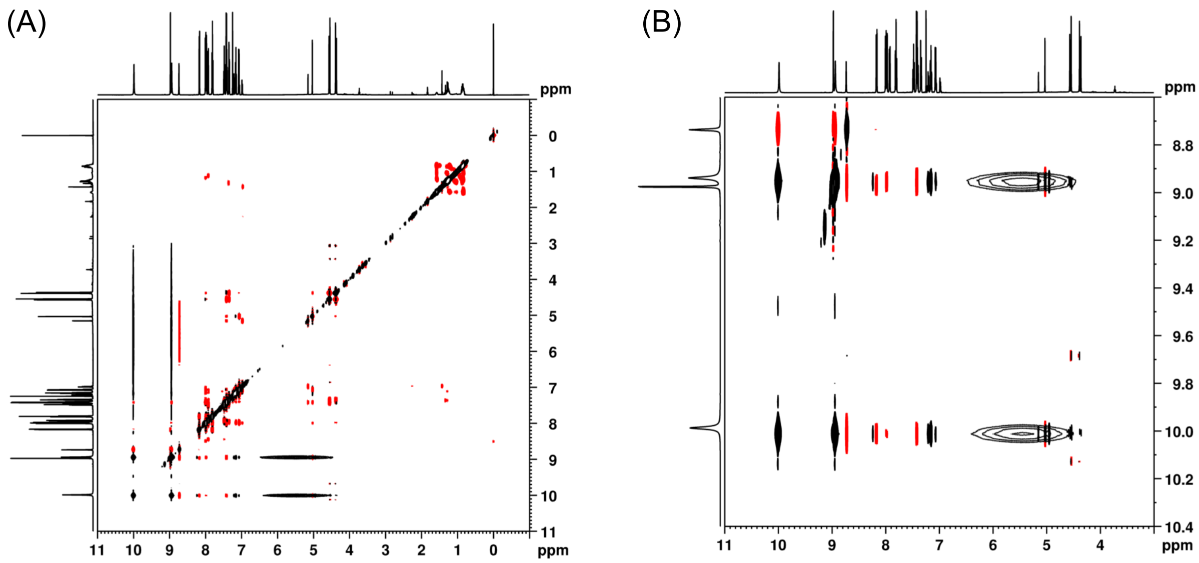

Figure S39: 600 MHz ^{1}H–^{1}H EXSY contour maps for an enantiomeric mixture of mandelic acid (MA), containing 70% (*R*)-MA, in the presence of the chiral macrocycle in $CDCl_3$. The spectrum was recorded at 50.0 °C with a mixing time of 2000 ms. **A)** Full spectral region and **B)** expanded view of the 3.0–11.0 ppm and 8.6–10.4 ppm regions. Exchange cross-peaks are highlighted in black, whereas NOE cross-peaks are shown in red. The contour plot intensity was divided by a factor of 4.

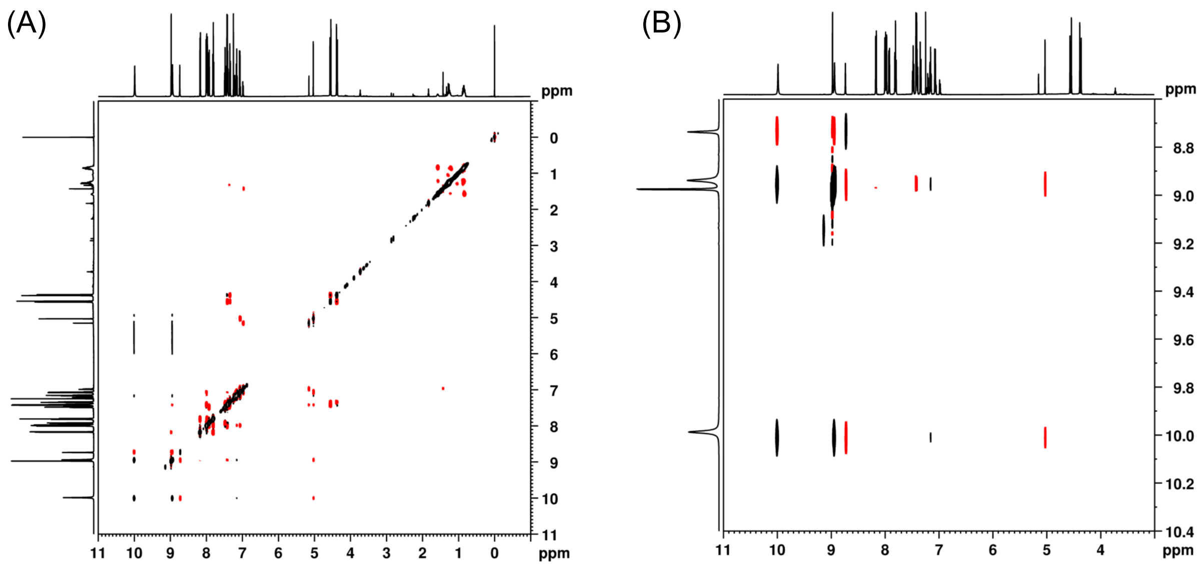

Figure S40: 600 MHz ^{1}H–^{1}H EXSY contour maps for an enantiomeric mixture of mandelic acid (MA), containing 70% (*R*)-MA, in the presence of the chiral macrocycle in $CDCl_3$. The spectrum was recorded at 50.0 °C with a mixing time of 3000 ms. **A)** Full spectral region and **B)** expanded view of the 3.0–11.0 ppm and 8.6–10.4 ppm regions. Exchange cross-peaks are highlighted in black, whereas NOE cross-peaks are shown in red. The contour plot intensity was divided by a factor of 6.

3.5.2 EXSY Experiments at 25.0 °C

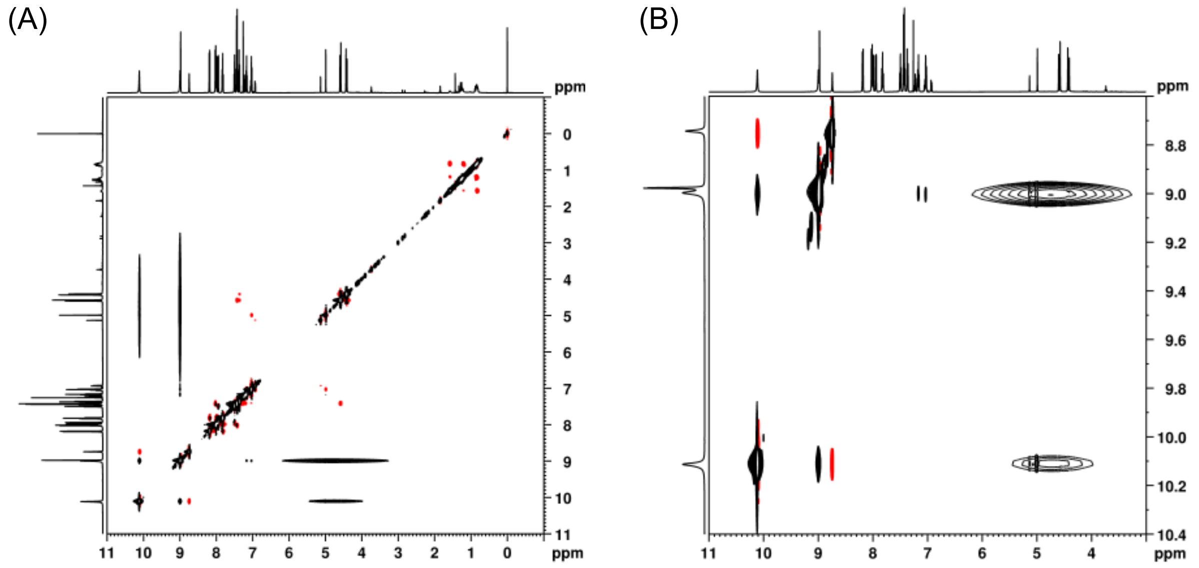

Figure S41: 600 MHz ^{1}H–^{1}H EXSY contour maps for an enantiomeric mixture of mandelic acid (MA), containing 70% (*R*)-MA, in the presence of the chiral macrocycle in $CDCl_3$. The spectrum was recorded at 25.0 °C with a mixing time of 500 ms. **A)** Full spectral region and **B)** expanded view of the 3.0–11.0 ppm and 8.6–10.4 ppm regions. Exchange cross-peaks are highlighted in black, whereas NOE cross-peaks are shown in red.

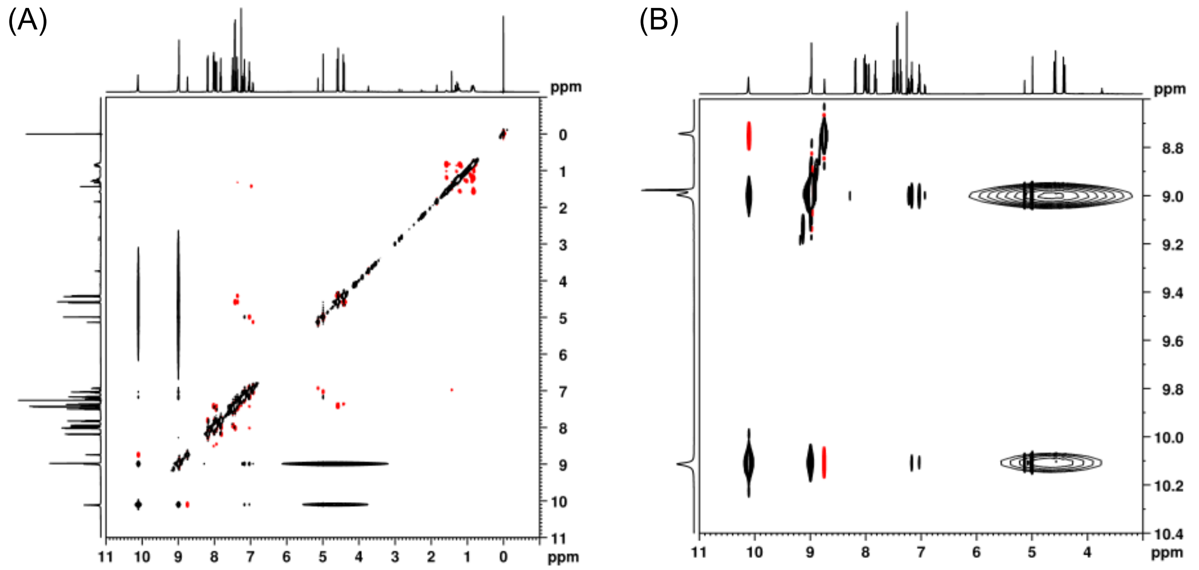

Figure S42: 600 MHz ^{1}H–^{1}H EXSY contour maps for an enantiomeric mixture of mandelic acid (MA), containing 70% (*R*)-MA, in the presence of the chiral macrocycle in $CDCl_3$. The spectrum was recorded at 25.0 °C with a mixing time of 1000 ms. **A)** Full spectral region and **B)** expanded view of the 3.0–11.0 ppm and 8.6–10.4 ppm regions. Exchange cross-peaks are highlighted in black, whereas NOE cross-peaks are shown in red. The contour plot intensity was divided by a factor of 4.

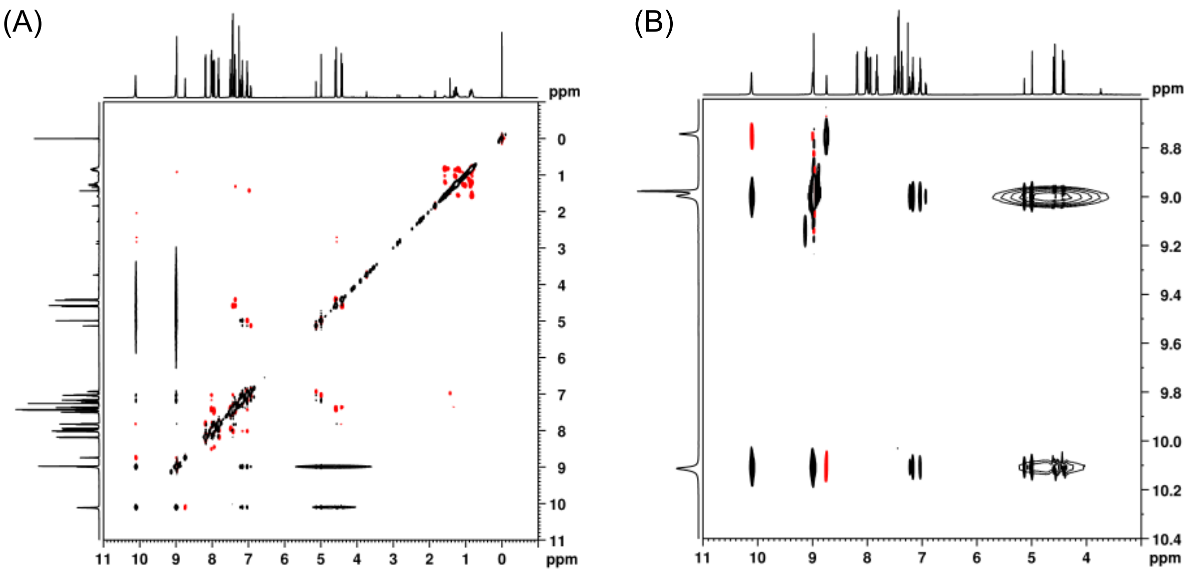

Figure S43: 600 MHz ^{1}H–^{1}H EXSY contour maps for an enantiomeric mixture of mandelic acid (MA), containing 70% (*R*)-MA, in the presence of the chiral macrocycle in $CDCl_3$. The spectrum was recorded at 25.0 °C with a mixing time of 2000 ms. **A)** Full spectral region and **B)** expanded view of the 3.0–11.0 ppm and 8.6–10.4 ppm regions. Exchange cross-peaks are highlighted in black, whereas NOE cross-peaks are shown in red. The contour plot intensity was divided by a factor of 6.

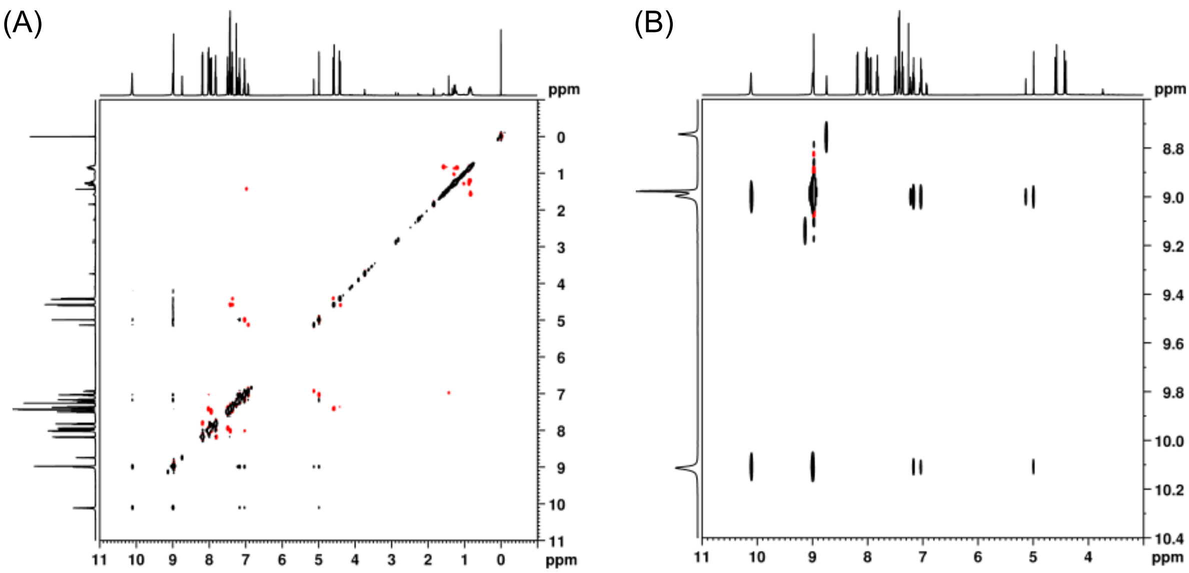

Figure S44: 600 MHz ^{1}H–^{1}H EXSY contour maps for an enantiomeric mixture of mandelic acid (MA), containing 70% (*R*)-MA, in the presence of the chiral macrocycle in $CDCl_3$. The spectrum was recorded at 25.0 °C with a mixing time of 3000 ms. **A)** Full spectral region and **B)** expanded view of the 3.0–11.0 ppm and 8.6–10.4 ppm regions. Exchange cross-peaks are highlighted in black, whereas NOE cross-peaks are shown in red. The contour plot intensity was multiplied by a factor of 8.

3.5.3 EXSY Experiments at -25.0 °C

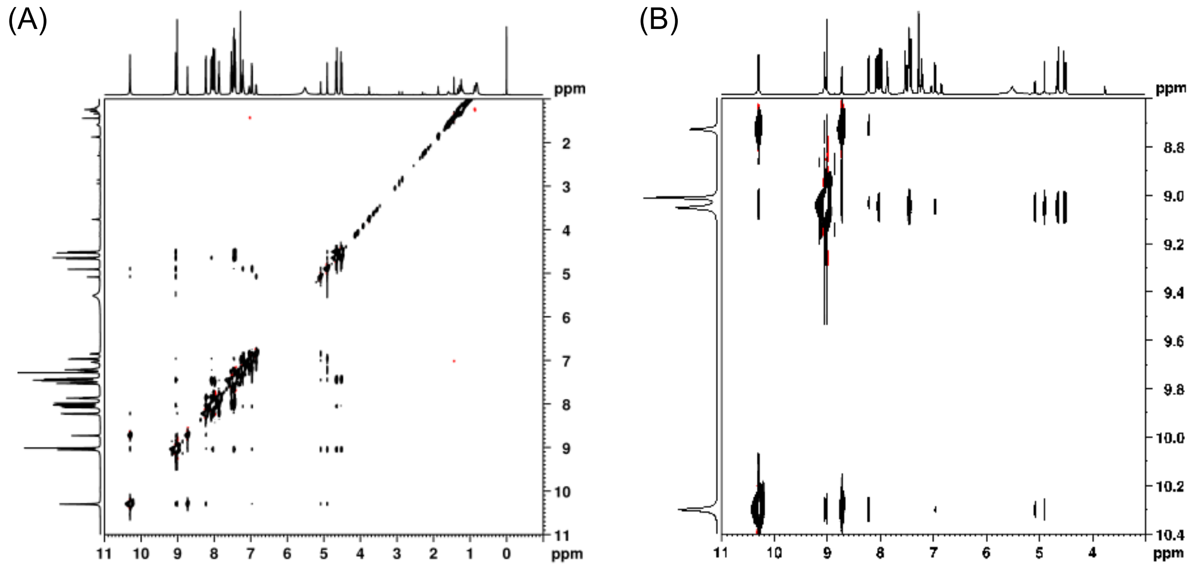

Figure S45: 600 MHz ^{1}H–^{1}H EXSY contour maps for an enantiomeric mixture of mandelic acid (MA), containing 70% (*R*)-MA, in the presence of the chiral macrocycle in $CDCl_3$. The spectrum was recorded at -25.0 °C with a mixing time of 500 ms. **A)** Full spectral region and **B)** expanded view of the 3.0–11.0 ppm and 8.6–10.4 ppm regions. Exchange cross-peaks are highlighted in black, whereas NOE cross-peaks are shown in red.

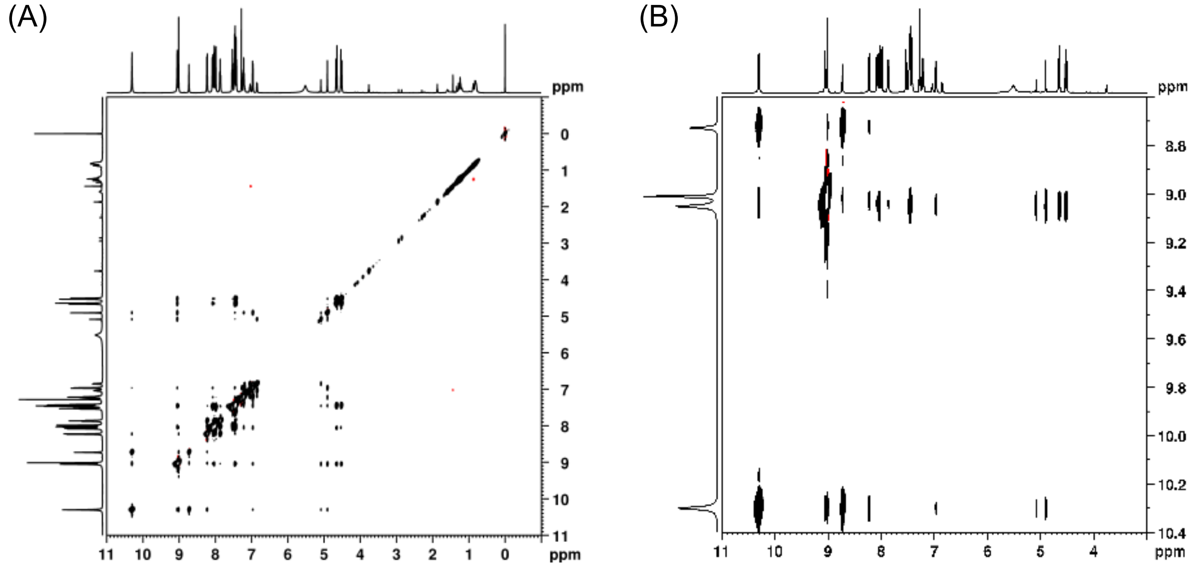

Figure S46: 600 MHz ^{1}H–^{1}H EXSY contour maps for an enantiomeric mixture of mandelic acid (MA), containing 70% (*R*)-MA, in the presence of the chiral macrocycle in $CDCl_3$. The spectrum was recorded at -25.0 °C with a mixing time of 1000 ms. **A)** Full spectral region and **B)** expanded view of the 3.0–11.0 ppm and 8.6–10.4 ppm regions. Exchange cross-peaks are highlighted in black, whereas NOE cross-peaks are shown in red. The contour plot intensity was divided by a factor of 12.

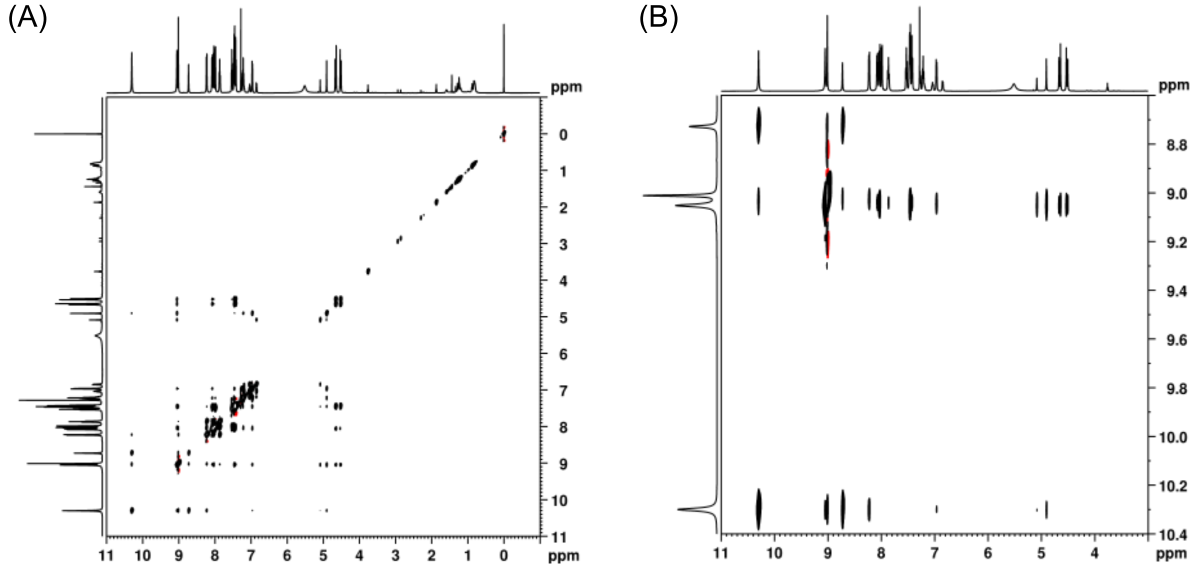

Figure S47: 600 MHz ^{1}H–^{1}H EXSY contour maps for an enantiomeric mixture of mandelic acid (MA), containing 70% (*R*)-MA, in the presence of the chiral macrocycle in $CDCl_3$. The spectrum was recorded at -25.0 °C with a mixing time of 2000 ms. **A)** Full spectral region and **B)** expanded view of the 3.0–11.0 ppm and 8.6–10.4 ppm regions. Exchange cross-peaks are highlighted in black, whereas NOE cross-peaks are shown in red. The contour plot intensity was divided by a factor of 12.

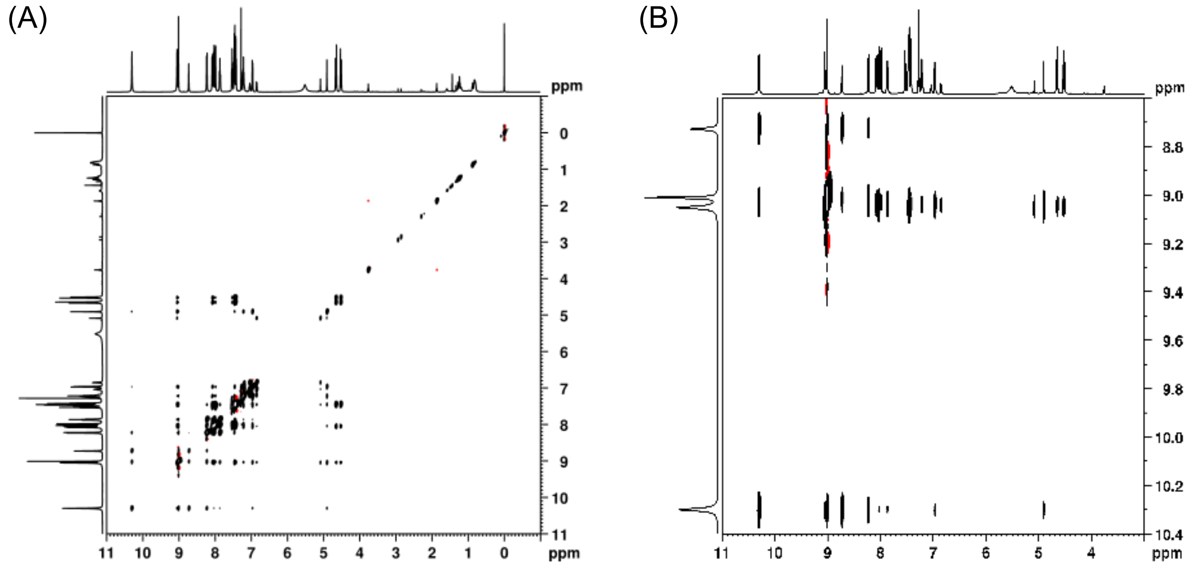

Figure S48: 600 MHz ^{1}H–^{1}H EXSY contour maps for an enantiomeric mixture of mandelic acid (MA), containing 70% (*R*)-MA, in the presence of the chiral macrocycle in $CDCl_3$. The spectrum was recorded at -25.0 °C with a mixing time of 3000 ms. **A)** Full spectral region and **B)** expanded view of the 3.0–11.0 ppm and 8.6–10.4 ppm regions. Exchange cross-peaks are highlighted in black, whereas NOE cross-peaks are shown in red. The contour plot intensity was divided by a factor of 10.

4 Further Information on Computational Results

The number of conformers retained at each stage of the workflow, together with the enantiomeric differentiation trends, calculated Gibbs free energies and the computed ^{1}H and $_{19}$F NMR chemical shifts for the final ensembles of each analyte are summarized below. Complete details, including

input files, optimized coordinates, output files, and additional energy and chemical shift data, are available in the freely accessible online repository at DOI: 10.25824/redu/ZL36LD.

4.1 Number of Diastereomeric Complex Conformers

Table S15: Number of diastereomeric complexes (homochiral and heterochiral) retained at each stage of the computational workflow using the *open* and *closed* conformations of the macrocycle (MAC) for the enantiodifferentiation of various chiral analytes.

Analyte	MAC Conf.	Complex Chirality	Conf. Search	REOPT	HESS	CLUSTER	PART0	PART1	PART2	PART3
Mandelic Acid	*open*	Homochiral	33	31	30	9	2	2	2	2
		Heterochiral	59	45	43	9	5	3	3	3
	closed	Homochiral	63	55	50	11	5	4	2	2
		Heterochiral	53	49	47	15	4	3	1	1
Camphor	*open*	Homochiral	166	127	117	27	6	5	5	5
		Heterochiral	111	83	80	18	8	6	6	6
	closed	Homochiral	299	270	250	22	10	3	3	3
		Heterochiral	377	293	270	33	12	5	4	4
Methylbenzylamine	*open*	Homochiral	79	69	66	26	12	10	4	2
		Heterochiral	27	25	25	4	3	3	3	3
	closed	Homochiral	184	154	127	33	20	8	6	6
		Heterochiral	201	144	134	36	7	3	2	2
2-Butanol	*open*	Homochiral	67	57	57	11	7	5	5	5
		Heterochiral	46	42	42	12	6	5	3	3
	closed	Homochiral	620	532	489	33	9	5	5	5
		Heterochiral	864	599	525	46	6	6	5	5
Mosher's Acid	*open*	Homochiral	84	74	70	25	3	2	2	2
		Heterochiral	142	65	52	11	7	6	1	1
	closed	Homochiral	134	116	109	26	1	1	1	1
		Heterochiral	72	54	50	13	5	5	3	3

4.2 General Trends in Computed Enantiomeric Discrimination

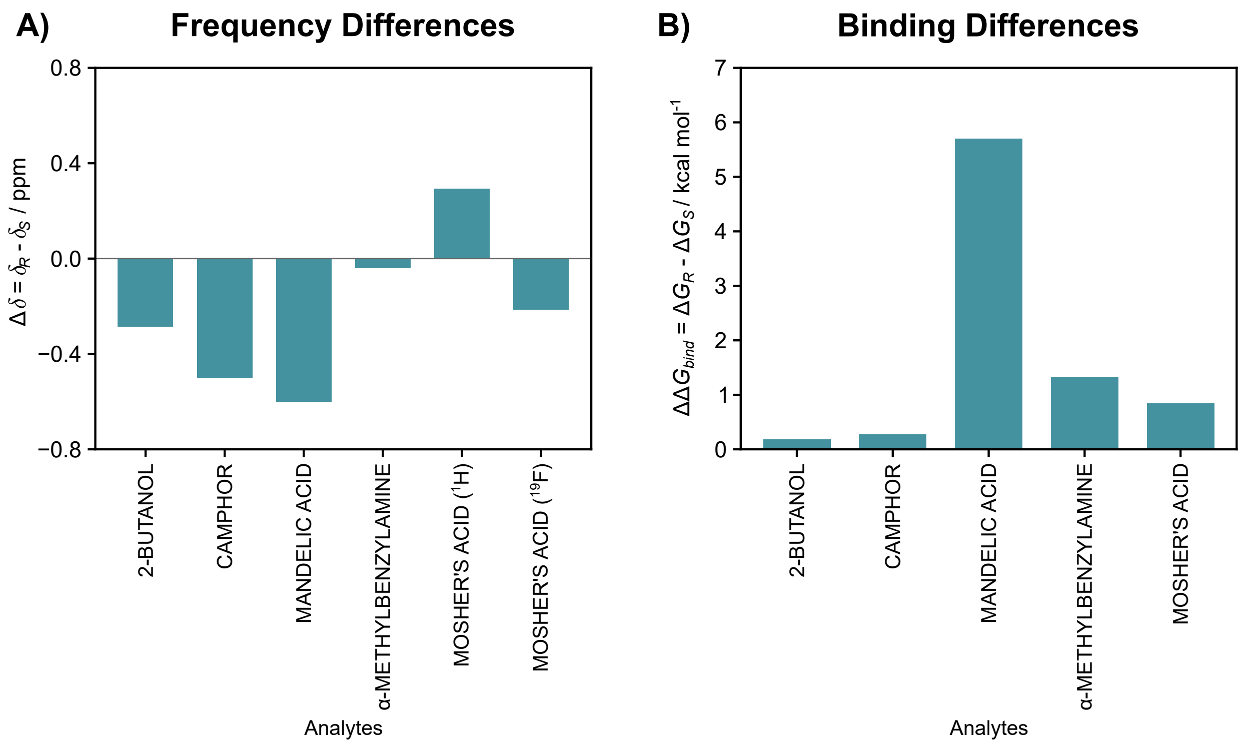

Figure S49: Comparison of the computed enantiomeric discrimination obtained for different analytes in the presence of the ***closed* chiral macrocycle**. **A)** Differences in chemical shifts (frequency separation), expressed as $\Delta\delta_{RS} = \delta_R - \delta_S$ (in ppm), where δ_R and δ_S correspond to the chemical shifts of the (*R*)- and (*S*)-enantiomers, respectively. **B)** Differences in binding Gibbs free energies, expressed as $\Delta\Delta G = \Delta G_R - \Delta G_S$ (in kcal mol^{-1}), where ΔG_R and ΔG_S correspond to the binding Gibbs free energies of the (*R*)- and (*S*)-enantiomers, respectively.

Table S16: Computed enantiomeric differences in chemical shifts ($\Delta\delta$, ppm) and binding Gibbs free energies ($\Delta\Delta G$, kcal mol^{-1}) for the investigated analytes in complexes with the ***closed*** conformation of the chiral macrocycle. The individual values for each enantiomer in the homochiral and heterochiral complexes are also reported.

**Analyte**	**Enantiodifferentiation**			**Homochiral (*RR*)**			**Heterochiral (*RS*)**		
	$\Delta\delta_H$	$\Delta\delta_F$	$\Delta\Delta G$	δ_H	δ_F	ΔG_{bind}	δ_H	δ_F	ΔG_{bind}
Mandelic Acid	-0.603	–	5.700	4.988	–	-1.100	5.591	–	-6.800
2-Butanol	-0.286	–	0.177	0.721	–	2.759	1.007	–	2.582
Camphor	-0.501	–	0.273	0.206	–	1.661	0.707	–	1.387
α-Methylbenzylamine	-0.039	–	1.328	1.212	–	1.130	1.251	–	-0.198
Mosher's Acid	0.293	-0.213	0.845	3.990	259.347	0.246	3.697	259.134	-0.600

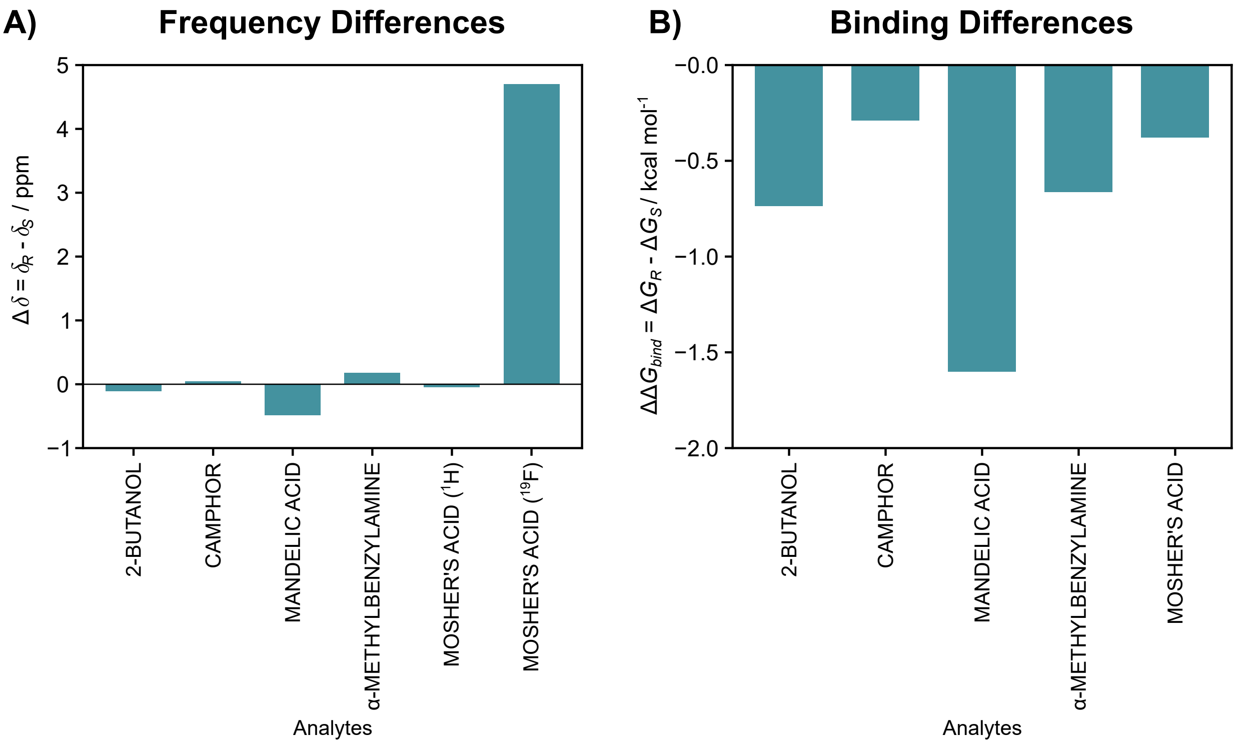

Figure S50: Comparison of the computed enantiomeric discrimination obtained for different analytes in the presence of the ***open* chiral macrocycle**. **A)** Differences in chemical shifts (frequency separation), expressed as $\Delta\delta_{RS} = \delta_R - \delta_S$ (in ppm), where δ_R and δ_S correspond to the chemical shifts of the (*R*)- and (*S*)-enantiomers, respectively. **B)** Differences in binding Gibbs free energies, expressed as $\Delta\Delta G = \Delta G_R - \Delta G_S$ (in kcal mol^{-1}), where ΔG_R and ΔG_S correspond to the binding Gibbs free energies of the (*R*)- and (*S*)-enantiomers, respectively.

Table S17: Computed enantiomeric differences in chemical shifts ($\Delta\delta$, ppm) and binding Gibbs free energies ($\Delta\Delta G$, kcal mol^{-1}) for the investigated analytes in complexes with the ***open*** conformation of the chiral macrocycle. The individual values for each enantiomer in the homochiral and heterochiral complexes are also reported.

**Analyte**	**Enantiodifferentiation**			**Homochiral (*RR*)**			**Heterochiral (*RS*)**		
	$\Delta\delta_{\mathrm{H}}$	$\Delta\delta_{\mathrm{F}}$	$\Delta\Delta G$	δ_{H}	δ_{F}	ΔG_{bind}	δ_{H}	δ_{F}	ΔG_{bind}
Mandelic Acid	-0.479	–	-1.600	5.126	–	-5.700	5.605	–	-4.100
2-Butanol	-0.109	–	-0.736	0.629	–	-1.577	0.738	–	-0.841
Camphor	0.043	–	-0.289	0.257	–	-1.358	0.213	–	-1.069
α-Methylbenzylamine	0.173	–	-0.662	1.106	–	-2.893	0.934	–	-2.231
Mosher's Acid	-0.044	4.700	-0.377	3.452	254.795	-4.134	3.495	259.495	-3.757

4.3 Diastereomeric Binding and Gibbs Free Energies

4.3.1 Refined Ensemble of the (*R*,*S*)-MA in Complex with the *Open* MAC

Table S18: Gibbs free energies of binding (in Hartree) computed for each homochiral complex conformation ($G_{complex}$), along with the individual contributions from the (*R*)-macrocycle (G_{MAC}) and (*R*)-mandelic acid ($G_{(R)\text{-MA}}$). The corresponding binding Gibbs free energies (ΔG_{bind}, in kcal/mol) and the Boltzmann-weighted values are also reported. All values are derived from the low-energy structures obtained after PART3, with energies computed at the ωB97X-V/def2-TZVPP/SMD level.

Conformer	$G_{complex}$	$G_{(R)\text{-MA}}$	G_{MAC}	ΔG_{bind}
CONF1	-3138.1164325	-535.3199811	-2602.7873463	-5.714
CONF2	-3138.1128443	-535.3199381	-2602.7873003	-3.518
**Boltzmann Avg.**	**-3138.1163540**	**-535.3199802**	**-2602.7873453**	**-5.666**

Table S19: Gibbs free energies of binding (in Hartree) computed for each heterochiral complex conformation ($G_{complex}$), along with the individual contributions from the (*R*)-macrocycle (G_{MAC}) and (*S*)-mandelic acid ($G_{(S)\text{-MA}}$). The corresponding binding Gibbs free energies (ΔG_{bind}, in kcal/mol) and the Boltzmann-weighted values are also reported. All values are derived from the low-energy structures obtained after PART3, with energies computed at the ωB97X-V/def2-TZVPP/SMD level.

Conformer	$G_{complex}$	$G_{(S)\text{-MA}}$	G_{MAC}	ΔG_{bind}
CONF1	-3138.1167181	-535.3199976	-2602.7901658	-4.113
CONF2	-3138.1129565	-535.3160440	-2602.7872246	-6.079
CONF3	-3138.1125532	-535.3199670	-2602.7882079	-2.747
**Boltzmann Avg.**	**-3138.1166012**	**-535.3199259**	**-2602.7900897**	**-4.133**

4.3.2 Refined Ensemble of the (*R*,*S*)-MA in Complex with the *Closed* MAC

Table S20: Gibbs free energies of binding (in Hartree) computed for each homochiral complex conformation ($G_{complex}$), along with the individual contributions from the (*R*)-macrocycle (G_{MAC}) and (*R*)-mandelic acid ($G_{(R)\text{-MA}}$). The corresponding binding Gibbs free energies (ΔG_{bind}, in kcal/mol) and the Boltzmann-weighted values are also reported. All values are derived from the low-energy structures obtained after PART3, with energies computed at the ωB97X-V/def2-TZVPP/SMD level.

Conformer	$G_{complex}$	$G_{(R)\text{-MA}}$	G_{MAC}	ΔG_{bind}
CONF1	-3138.1092775	-535.3200184	-2602.7876867	-0.987
CONF2	-3138.1067617	-535.3200010	-2602.7810445	-3.587
**Boltzmann Avg.**	**-3138.1091138**	**-535.3200173**	**-2602.7872544**	**-1.156**

Table S21: Gibbs free energies of binding (in Hartree) computed for each heterochiral complex conformation (G_{complex}), along with the individual contributions from the (*R*)-macrocycle (G_{MAC}) and (*S*)-mandelic acid ($G_{(S)\text{-MA}}$). The corresponding binding Gibbs free energies (ΔG_{bind}, in kcal/mol) and the Boltzmann-weighted values are also reported. All values are derived from the low-energy structures obtained after PART3, with energies computed at the ωB97X-V/def2-TZVPP/SMD level.

**Conformer**	G_{complex}	$G_{(S)\text{-MA}}$	G_{MAC}	ΔG_{bind}
CONF1	-3138.1147939	-535.3198443	-2602.7840870	-6.816

4.3.3 Refined Ensemble of the (*R*,*S*)-2-Butanol in Complex with the *Open* MAC

Table S22: Gibbs free energies of binding (in Hartree) computed for each homochiral complex conformation (G_{complex}), along with the individual contributions from the (*R*)-macrocycle (G_{MAC}) and (*R*)-2-butanol ($G_{(R)\text{-2-butanol}}$). The corresponding binding Gibbs free energies (ΔG_{bind}, in kcal/mol) and the Boltzmann-weighted values are also reported. All values are derived from the low-energy structures obtained after PART3, with energies computed at the ωB97X-V/def2-TZVPP/SMD level.

**Conformer**	G_{complex}	$G_{(R)\text{-2-butanol}}$	G_{MAC}	ΔG_{bind}
CONF1	-2836.3903272	-233.6008917	-2602.7874815	-1.226
CONF2	-2836.3901721	-233.6014508	-2602.7844359	-2.689
CONF3	-2836.3897534	-233.6021157	-2602.7875515	-0.054
CONF4	-2836.3896474	-233.6014713	-2602.7873587	-0.513
CONF5	-2836.3896260	-233.6001208	-2602.7844647	-3.163
**Boltzmann Avg.**	**-2836.3899968**	**-233.6012065**	**-2602.7862772**	**-1.577**

Table S23: Gibbs free energies of binding (in Hartree) computed for each heterochiral complex conformation (G_{complex}), along with the individual contributions from the (*R*)-macrocycle (G_{MAC}) and (*S*)-2-butanol ($G_{(S)\text{-2-butanol}}$). The corresponding binding Gibbs free energies (ΔG_{bind}, in kcal/mol) and the Boltzmann-weighted values are also reported. All values are derived from the low-energy structures obtained after PART3, with energies computed at the ωB97X-V/def2-TZVPP/SMD level.

**Conformer**	G_{complex}	$G_{(S)\text{-2-butanol}}$	G_{MAC}	ΔG_{bind}
CONF1	-2836.3936995	-233.6020502	-2602.7901287	-0.954
CONF2	-2836.3924910	-233.6014613	-2602.7902791	-0.471
CONF3	-2836.3905805	-233.6020681	-2602.7876184	-0.561
**Boltzmann Avg.**	**-2836.3933568**	**-233.6019262**	**-2602.7900903**	**-0.841**

4.3.4 Refined Ensemble of the (*R*,*S*)-2-Butanol in Complex with the *Closed* MAC

Table S24: Gibbs free energies of binding (in Hartree) computed for each homochiral complex conformation ($G_{complex}$), along with the individual contributions from the (*R*)-macrocycle (G_{MAC}) and (*R*)-2-butanol ($G_{(R)\text{-2-butanol}}$). The corresponding binding Gibbs free energies (ΔG_{bind}, in kcal/mol) and the Boltzmann-weighted values are also reported.

Conformer	$G_{complex}$	$G_{(R)\text{-2-butanol}}$	G_{MAC}	ΔG_{bind}
CONF1	-2836.3819760	-233.6014547	-2602.7850869	2.865
CONF2	-2836.3806662	-233.6014720	-2602.7828234	2.277
CONF3	-2836.3804350	-233.6014771	-2602.7828500	2.442
CONF4	-2836.3782456	-233.6014050	-2602.7829315	3.822
CONF5	-2836.3781380	-233.6014520	-2602.7863206	6.046
**Boltzmann Avg.**	**-2836.3814590**	**-233.6014599**	**-2602.7843965**	**2.759**

Table S25: Gibbs free energies of binding (in Hartree) computed for each heterochiral complex conformation ($G_{complex}$), along with the individual contributions from the (*R*)-macrocycle (G_{MAC}) and (*S*)-2-butanol ($G_{(S)\text{-2-butanol}}$). The corresponding binding Gibbs free energies (ΔG_{bind}, in kcal/mol) and the Boltzmann-weighted values are also reported.

Conformer	$G_{complex}$	$G_{(S)\text{-2-butanol}}$	G_{MAC}	ΔG_{bind}
CONF1	-2836.3819834	-233.6014491	-2602.7852434	2.955
CONF2	-2836.3819029	-233.6008581	-2602.7852897	2.664
CONF3	-2836.3818966	-233.6014358	-2602.7846916	2.655
CONF4	-2836.3818715	-233.6018901	-2602.7846313	2.918
CONF5	-2836.3816807	-233.6014112	-2602.7825999	1.462
**Boltzmann Avg.**	**-2836.3818772**	**-233.6014062**	**-2602.7845857**	**2.582**

4.3.5 Refined Ensemble of the (*R*,*S*)-Camphor in Complex with the *Open* MAC

Table S26: Gibbs free energies of binding (in Hartree) computed for each homochiral complex conformation ($G_{complex}$), along with the individual contributions from the (*R*)-macrocycle (G_{MAC}) and (*R*)-Camphor ($G_{(R)\text{-Camphor}}$). The corresponding binding Gibbs free energies (ΔG_{bind}, in kcal/mol) and the Boltzmann-weighted values are also reported. All values are derived from the low-energy structures obtained after PART3, with energies computed at the ωB97X-V/def2-TZVPP/SMD level.

Conformer	$G_{complex}$	$G_{(R)\text{-Camphor}}$	G_{MAC}	ΔG_{bind}
CONF1	-3068.5913485	-465.8012711	-2602.7871345	-1.847
CONF2	-3068.5911214	-465.8012861	-2602.7872650	-1.613
CONF3	-3068.5910498	-465.8012637	-2602.7872065	-1.619
CONF4	-3068.5907116	-465.8012673	-2602.7897227	0.175
CONF5	-3068.5895901	-465.8012292	-2602.7872050	-0.725
**Boltzmann Avg.**	**-3068.5910360**	**-465.8012705**	**-2602.7876013**	**-1.358**

Table S27: Gibbs free energies of binding (in Hartree) computed for each heterochiral complex conformation (G_{complex}), along with the individual contributions from the (*R*)-macrocycle (G_{MAC}) and (*S*)-Camphor ($G_{(S)\text{-Camphor}}$). The corresponding binding Gibbs free energies (ΔG_{bind}, in kcal/mol) and the Boltzmann-weighted values are also reported. All values are derived from the low-energy structures obtained after PART3, with energies computed at the ωB97X-V/def2-TZVPP/SMD level.

**Conformer**	G_{complex}	$G_{(S)\text{-Camphor}}$	G_{MAC}	ΔG_{bind}
CONF1	-3068.5920958	-465.8012608	-2602.7907551	-0.050
CONF2	-3068.5914510	-465.8012607	-2602.7872277	-1.859
CONF3	-3068.5913769	-465.8012593	-2602.7871777	-1.845
CONF4	-3068.5911067	-465.8012849	-2602.7872107	-1.638
CONF5	-3068.5904181	-465.8012297	-2602.7872749	-1.201
CONF6	-3068.5904063	-465.8012302	-2602.7871295	-1.284
**Boltzmann Avg.**	**-3068.5915037**	**-465.8012598**	**-2602.7885401**	**-1.069**

4.3.6 Refined Ensemble of the (*R*,*S*)-Camphor in Complex with the *Closed* MAC

Table S28: Gibbs free energies of binding (in Hartree) computed for each homochiral complex conformation (G_{complex}), along with the individual contributions from the (*R*)-macrocycle (G_{MAC}) and (*R*)-Camphor ($G_{(R)\text{-Camphor}}$). The corresponding binding Gibbs free energies (ΔG_{bind}, in kcal/mol) and the Boltzmann-weighted values are also reported.

**Conformer**	G_{complex}	$G_{(R)\text{-Camphor}}$	G_{MAC}	ΔG_{bind}
CONF1	-3068.5842870	-465.8012573	-2602.7870311	2.511
CONF2	-3068.5839849	-465.8012376	-2602.7823654	-0.240
CONF3	-3068.5833391	-465.8012537	-2602.7870358	3.106
**Boltzmann Avg.**	**-3068.5840162**	**-465.8012498**	**-2602.7854128**	**1.661**

Table S29: Gibbs free energies of binding (in Hartree) computed for each heterochiral complex conformation (G_{complex}), along with the individual contributions from the (*R*)-macrocycle (G_{MAC}) and (*S*)-Camphor ($G_{(S)\text{-Camphor}}$). The corresponding binding Gibbs free energies (ΔG_{bind}, in kcal/mol) and the Boltzmann-weighted values are also reported.

**Conformer**	G_{complex}	$G_{(S)\text{-Camphor}}$	G_{MAC}	ΔG_{bind}
CONF1	-3068.5849188	-465.8012550	-2602.7852463	0.993
CONF2	-3068.5844455	-465.8012176	-2602.7876395	2.768
CONF3	-3068.5833324	-465.8012622	-2602.7822454	0.110
CONF4	-3068.5832989	-465.8012599	-2602.7824391	0.251
**Boltzmann Avg.**	**-3068.5844758**	**-465.8012446**	**-2602.7854419**	**1.387**

4.3.7 Refined Ensemble of the (*R*,*S*)-Methylbenzylamine in Complex with the *Open* MAC

Table S30: Gibbs free energies of binding (in Hartree) computed for each homochiral complex conformation (G_{complex}), along with the individual contributions from the (*R*)-macrocycle (G_{MAC}) and (*R*)-Methylbenzylamine ($G_{(R)\text{-Methylbenzylamine}}$). The corresponding binding Gibbs free energies (ΔG_{bind}, in kcal/mol) and the Boltzmann-weighted values are also reported. All values are derived from the low-energy structures obtained after PART3, with energies computed at the ωB97X-V/def2-TZVPP/SMD level.

Conformer	G_{complex}	$G_{(R)\text{-Methylbenzylamine}}$	G_{MAC}	ΔG_{bind}
CONF1	-2968.9314889	-366.1368654	-2602.7899710	-2.919
CONF2	-2968.9292771	-366.1349465	-2602.7901686	-2.612
**Boltzmann Avg.**	**-2968.9312951**	**-366.1366972**	**-2602.7899883**	**-2.893**

Table S31: Gibbs free energies of binding (in Hartree) computed for each heterochiral complex conformation (G_{complex}), along with the individual contributions from the (*R*)-macrocycle (G_{MAC}) and (*S*)-Methylbenzylamine ($G_{(S)\text{-Methylbenzylamine}}$). The corresponding binding Gibbs free energies (ΔG_{bind}, in kcal/mol) and the Boltzmann-weighted values are also reported. All values are derived from the low-energy structures obtained after PART3, with energies computed at the ωB97X-V/def2-TZVPP/SMD level.

Conformer	G_{complex}	$G_{(S)\text{-Methylbenzylamine}}$	G_{MAC}	ΔG_{bind}
CONF1	-2968.9263502	-366.1354305	-2602.7871724	-2.351
CONF2	-2968.9259271	-366.1348125	-2602.7871692	-2.476
CONF3	-2968.9243294	-366.1372647	-2602.7872698	0.129
**Boltzmann Avg.**	**-2968.9260610**	**-366.1353285**	**-2602.7871778**	**-2.231**

4.3.8 Refined Ensemble of the (*R*,*S*)-Methylbenzylamine in Complex with the *Closed* MAC

Table S32: Gibbs free energies of binding (in Hartree) computed for each homochiral complex conformation (G_{complex}), along with the individual contributions from the (*R*)-macrocycle (G_{MAC}) and (*R*)-Methylbenzylamine ($G_{(R)\text{-Methylbenzylamine}}$). The corresponding binding Gibbs free energies (ΔG_{bind}, in kcal/mol) and the Boltzmann-weighted values are also reported.

Conformer	G_{complex}	$G_{(R)\text{-Methylbenzylamine}}$	G_{MAC}	ΔG_{bind}
CONF1	-2968.9220679	-366.1368882	-2602.7868006	1.017
CONF2	-2968.9199191	-366.1368935	-2602.7845333	0.946
CONF3	-2968.9188839	-366.1368818	-2602.7852093	2.013
CONF4	-2968.9187111	-366.1354454	-2602.7874320	2.614
CONF5	-2968.9184628	-366.1354335	-2602.7868104	2.373
CONF6	-2968.9183865	-366.1354537	-2602.7872337	2.699
**Boltzmann Avg.**	**-2968.9215881**	**-366.1368038**	**-2602.7865850**	**1.130**

Table S33: Gibbs free energies of binding (in Hartree) computed for each heterochiral complex conformation (G_{complex}), along with the individual contributions from the (*R*)-macrocycle (G_{MAC}) and (*S*)-Methylbenzylamine ($G_{(S)\text{-Methylbenzylamine}}$). The corresponding binding Gibbs free energies (ΔG_{bind}, in kcal/mol) and the Boltzmann-weighted values are also reported.

**Conformer**	G_{complex}	$G_{(S)\text{-Methylbenzylamine}}$	G_{MAC}	ΔG_{bind}
CONF1	-2968.9228641	-366.1354491	-2602.7863830	-0.648
CONF2	-2968.9216016	-366.1354217	-2602.7885916	1.513
**Boltzmann Avg.**	**-2968.9226015**	**-366.1354434**	**-2602.7868423**	**-0.198**

4.3.9 Refined Ensemble of the (*R*,*S*)-Mosher's Acid in Complex with the *Open* MAC

Table S34: Gibbs free energies of binding (in Hartree) computed for each homochiral complex conformation (G_{complex}), along with the individual contributions from the (*R*)-macrocycle (G_{MAC}) and (*R*)-Mosher's Acid ($G_{(R)\text{-Mosher's Acid}}$). The corresponding binding Gibbs free energies (ΔG_{bind}, in kcal/mol) and the Boltzmann-weighted values are also reported. All values are derived from the low-energy structures obtained after PART3, with energies computed at the ωB97X-V/def2-TZVPP/SMD level.

**Conformer**	G_{complex}	$G_{(R)\text{-Mosher's Acid}}$	G_{MAC}	ΔG_{bind}
CONF1	-3514.4713168	-911.6745379	-2602.7901799	-4.141
CONF2	-3514.4673020	-911.6742719	-2602.7872475	-3.629
**Boltzmann Avg.**	**-3514.4712605**	**-911.6745342**	**-2602.7901388**	**-4.134**

Table S35: Gibbs free energies of binding (in Hartree) computed for each heterochiral complex conformation (G_{complex}), along with the individual contributions from the (*R*)-macrocycle (G_{MAC}) and (*S*)-Mosher's Acid ($G_{(S)\text{-Mosher's Acid}}$). The corresponding binding Gibbs free energies (ΔG_{bind}, in kcal/mol) and the Boltzmann-weighted values are also reported. All values are derived from the low-energy structures obtained after PART3, with energies computed at the ωB97X-V/def2-TZVPP/SMD level.

**Conformer**	G_{complex}	$G_{(S)\text{-Mosher's Acid}}$	G_{MAC}	ΔG_{bind}
CONF1	-3514.4711216	-911.6779032	-2602.7872319	-3.757

4.3.10 Refined Ensemble of the (*R*,*S*)-Mosher's Acid in Complex with the *Closed* MAC

Table S36: Gibbs free energies of binding (in Hartree) computed for each homochiral complex conformation (G_{complex}), along with the individual contributions from the (*R*)-macrocycle (G_{MAC}) and (*R*)-Mosher's Acid ($G_{(R)\text{-Mosher's Acid}}$). The corresponding binding Gibbs free energies (ΔG_{bind}, in kcal/mol) and the Boltzmann-weighted values are also reported.

**Conformer**	G_{complex}	$G_{(R)\text{-Mosher's Acid}}$	G_{MAC}	ΔG_{bind}
CONF1	-3514.4672735	-911.6793333	-2602.7883316	0.246

Table S37: Gibbs free energies of binding (in Hartree) computed for each heterochiral complex conformation (G_{complex}), along with the individual contributions from the (*R*)-macrocycle (G_{MAC}) and (*S*)-Mosher's Acid ($G_{(S)\text{-Mosher's Acid}}$). The corresponding binding Gibbs free energies (ΔG_{bind}, in kcal/mol) and the Boltzmann-weighted values are also reported.

**Conformer**	G_{complex}	$G_{(S)\text{-Mosher's Acid}}$	G_{MAC}	ΔG_{bind}
CONF1	-3514.4634048	-911.6792567	-2602.7841590	0.007
CONF2	-3514.4631010	-911.6780579	-2602.7830898	-1.226
CONF3	-3514.4627382	-911.6779856	-2602.7833033	-0.909
**Boltzmann Avg.**	**-3514.4631572**	**-911.6785822**	**-2602.7836193**	**-0.600**

4.4 Calculated NMR Chemical Shifts

4.4.1 Refined Ensemble of the (*R*,*S*)-MA in Complex with the *Open* MAC

Table S38: Calculated ^{1}H-NMR chemical shifts (δ, ppm) for the investigated nuclei of mandelic acid (MA) in each homochiral complex conformation. All geometries originate from the CREST conformational sampling and CENSO refinement. Chemical shifts were computed at the ωB97X–V/def2-TZVPP/SMD level of theory.

**Conformer**	$\delta_{(\boldsymbol{R})\text{-MA}}$
CONF1	5.151
CONF2	4.025
**Boltzmann Avg.**	**5.126**

Table S39: Calculated ^{1}H-NMR chemical shifts (δ, ppm) for the investigated nuclei of mandelic acid (MA) in each heterochiral complex conformation. All geometries originate from the CREST conformational sampling and CENSO refinement. Chemical shifts were computed at the ωB97X–V/def2-TZVPP/SMD level of theory.

**Conformer**	$\delta_{(\boldsymbol{S})\text{-MA}}$
CONF1	5.602
CONF2	5.792
CONF3	5.574
**Boltzmann Avg.**	**5.605**

4.4.2 Refined Ensemble of the (*R*,*S*)-MA Complex with the *Closed* MAC

Table S40: Calculated ^{1}H-NMR chemical shifts (δ, ppm) for the investigated nuclei of mandelic acid (MA) in each homochiral complex conformation. All geometries originate from the CREST conformational sampling and CENSO refinement. Chemical shifts were computed at the ωB97X–V/def2-TZVPP/SMD level of theory.

Conformer	$\delta_{(R)\text{-MA}}$
CONF1	5.037
CONF2	4.280
**Boltzmann Avg.**	**4.988**

Table S41: Calculated ^{1}H-NMR chemical shifts (δ, ppm) for the investigated nuclei of mandelic acid (MA) in each heterochiral complex conformation. All geometries originate from the CREST conformational sampling and CENSO refinement. Chemical shifts were computed at the ωB97X–V/def2-TZVPP/SMD level of theory.

Conformer	$\delta_{(S)\text{-MA}}$
CONF1	5.591

4.4.3 Refined Ensemble of the (*R*,*S*)-2-butanol in Complex with the *Open* MAC

Table S42: Calculated ^{1}H-NMR chemical shifts (δ, ppm) for the investigated nuclei of 2-butanol in each homochiral complex conformation. All geometries originate from the CREST conformational sampling and CENSO refinement. Chemical shifts were computed at the ωB97X–V/def2-TZVPP/SMD level of theory.

Conformer	$\delta_{(R)\text{-2-butanol}}$ CH_3 (triplet)	$\delta_{(R)\text{-2-butanol}}$ CH_3 (doublet)
CONF1	-0.349	0.871
CONF2	0.377	0.989
CONF3	0.391	1.870
CONF4	0.152	0.952
CONF5	0.776	0.841
**Boltzmann Avg.**	**0.187**	**1.070**

Table S43: Calculated ^{1}H-NMR chemical shifts (δ, ppm) for the investigated nuclei of 2-butanol in each heterochiral complex conformation. All geometries originate from the CREST conformational sampling and CENSO refinement. Chemical shifts were computed at the ωB97X–V/def2-TZVPP/SMD level of theory.

Conformer	$\delta_{(S)\text{-2-butanol}}$ CH_3 (triplet)	$\delta_{(S)\text{-2-butanol}}$ CH_3 (doublet)
CONF1	1.053	0.441
CONF2	0.187	1.284
CONF3	0.639	0.372
**Boltzmann Avg.**	**0.859**	**0.617**

4.4.4 Refined Ensemble of the (*R*,*S*)-2-butanol Complex with the *Closed* MAC

Table S44: Calculated ^{1}H-NMR chemical shifts (δ, ppm) for the investigated nuclei of 2-butanol in each homochiral complex conformation. All geometries originate from the CREST conformational sampling and CENSO refinement. Chemical shifts were computed at the ωB97X–V/def2-TZVPP/SMD level of theory.

Conformer	$\delta_{(R)\text{-2-butanol}}$ CH_3 (triplet)	$\delta_{(R)\text{-2-butanol}}$ CH_3 (doublet)
CONF1	0.143	1.213
CONF2	1.318	0.772
CONF3	0.449	0.548
CONF4	0.216	1.357
CONF5	0.750	1.155
**Boltzmann Avg.**	**0.389**	**1.052**

Table S45: Calculated ^{1}H-NMR chemical shifts (δ, ppm) for the investigated nuclei of 2-butanol in each heterochiral complex conformation. All geometries originate from the CREST conformational sampling and CENSO refinement. Chemical shifts were computed at the ωB97X–V/def2-TZVPP/SMD level of theory.

Conformer	$\delta_{(S)\text{-2-butanol}}$ CH_3 (triplet)	$\delta_{(S)\text{-2-butanol}}$ CH_3 (doublet)
CONF1	0.806	1.432
CONF2	1.038	1.272
CONF3	0.483	1.319
CONF4	0.737	1.440
CONF5	-0.127	1.527
**Boltzmann Avg.**	**0.621**	**1.393**

4.4.5 Refined Ensemble of the (*R*,*S*)-Camphor in Complex with the *Open* MAC

Table S46: Calculated ^{1}H-NMR chemical shifts (δ, ppm) for the investigated nuclei of Camphor in each homochiral complex conformation. All geometries originate from the CREST conformational sampling and CENSO refinement. Chemical shifts were computed at the ωB97X–V/def2-TZVPP/SMD level of theory.

Conformer	$\delta_{(R)\text{-Camphor}}$ CH_3 (singlet)
CONF1	0.269
CONF2	0.380
CONF3	-0.048
CONF4	0.434
CONF5	0.406
**Boltzmann Avg.**	**0.257**

Table S47: Calculated ^{1}H-NMR chemical shifts (δ, ppm) for the investigated nuclei of Camphor in each heterochiral complex conformation. All geometries originate from the CREST conformational sampling and CENSO refinement. Chemical shifts were computed at the ωB97X–V/def2-TZVPP/SMD level of theory.

Conformer	$\delta_{(S)\text{-Camphor}}$ CH_3 (singlet)
CONF1	0.383
CONF2	0.332
CONF3	0.328
CONF4	-0.439
CONF5	0.070
CONF6	0.035
**Boltzmann Avg.**	**0.213**

4.4.6 Refined Ensemble of the (*R*,*S*)-Camphor Complex with the *Closed* MAC

Table S48: Calculated ^{1}H-NMR chemical shifts (δ, ppm) for the investigated nuclei of Camphor in each homochiral complex conformation. All geometries originate from the CREST conformational sampling and CENSO refinement. Chemical shifts were computed at the ωB97X–V/def2-TZVPP/SMD level of theory.

Conformer	$\delta_{(R)\text{-Camphor}}$ CH_3 (singlet)
CONF1	0.460
CONF2	-0.459
CONF3	0.829
**Boltzmann Avg.**	**0.206**

Table S49: Calculated ^{1}H-NMR chemical shifts (δ, ppm) for the investigated nuclei of Camphor in each heterochiral complex conformation. All geometries originate from the CREST conformational sampling and CENSO refinement. Chemical shifts were computed at the ωB97X–V/def2-TZVPP/SMD level of theory.

Conformer	$\delta_{(S)\text{-Camphor}}$ CH_3 (singlet)
CONF1	0.536
CONF2	1.113
CONF3	0.843
CONF4	0.151
**Boltzmann Avg.**	**0.707**

4.4.7 Refined Ensemble of the (*R*,*S*)-Methylbenzylamine in Complex with the *Open* MAC

Table S50: Calculated ^{1}H-NMR chemical shifts (δ, ppm) for the investigated nuclei of Methylbenzylamine in each homochiral complex conformation. All geometries originate from the CREST conformational sampling and CENSO refinement. Chemical shifts were computed at the ωB97X–V/def2-TZVPP/SMD level of theory.

**Conformer**	$\delta_{(R)\text{-Methylbenzylamine}}$ **CH_3 (doublet)**
CONF1	1.056
CONF2	1.627
**Boltzmann Avg.**	**1.106**

Table S51: Calculated ^{1}H-NMR chemical shifts (δ, ppm) for the investigated nuclei of Methylbenzylamine in each heterochiral complex conformation. All geometries originate from the CREST conformational sampling and CENSO refinement. Chemical shifts were computed at the ωB97X–V/def2-TZVPP/SMD level of theory.

**Conformer**	$\delta_{(S)\text{-Methylbenzylamine}}$ **CH_3 (doublet)**
CONF1	0.894
CONF2	0.772
CONF3	2.149
**Boltzmann Avg.**	**0.934**

4.4.8 Refined Ensemble of the (*R*,*S*)-Methylbenzylamine Complex with the *Closed* MAC

Table S52: Calculated ^{1}H-NMR chemical shifts (δ, ppm) for the investigated nuclei of Methylbenzylamine in each homochiral complex conformation. All geometries originate from the CREST conformational sampling and CENSO refinement. Chemical shifts were computed at the ωB97X–V/def2-TZVPP/SMD level of theory.

**Conformer**	$\delta_{(R)\text{-Methylbenzylamine}}$ **CH_3 (doublet)**
CONF1	1.196
CONF2	1.557
CONF3	0.856
CONF4	1.350
CONF5	0.549
CONF6	1.351
**Boltzmann Avg.**	**1.212**

Table S53: Calculated ^{1}H-NMR chemical shifts (δ, ppm) for the investigated nuclei of Methylbenzylamine in each heterochiral complex conformation. All geometries originate from the CREST conformational sampling and CENSO refinement. Chemical shifts were computed at the ωB97X–V/def2-TZVPP/SMD level of theory.

**Conformer**	$\delta_{(S)\text{-Methylbenzylamine}}$ **CH_3 (doublet)**
CONF1	1.232
CONF2	1.322
**Boltzmann Avg.**	**1.251**

4.4.9 Refined Ensemble of the (*R*,*S*)-Mosher's Acid in Complex with the *Open* MAC

Table S54: Calculated ^{1}H-NMR chemical shifts (δ, ppm) and ^{19}F-NMR chemical shielding (σ, ppm) for the investigated nuclei of Mosher's Acid in each homochiral complex conformation. All geometries originate from the CREST conformational sampling and CENSO refinement. Chemical shifts were computed at the ωB97X–V/def2-TZVPP/SMD level of theory.

**Conformer**	$\delta_{(R)\text{-Mosher}}$ **CH_3 (singlet)**	$\sigma_{(R)\text{-Mosher}}$ **CF_3 (singlet)**
CONF1	3.457	254.776
CONF2	3.042	256.163
**Boltzmann Avg.**	**3.452**	**254.795**

Table S55: Calculated ^{1}H-NMR chemical shifts (δ, ppm) and ^{19}F-NMR chemical shielding (σ, ppm) for the investigated nuclei of Mosher's Acid in each heterochiral complex conformation. All geometries originate from the CREST conformational sampling and CENSO refinement. Chemical shifts were computed at the ωB97X–V/def2-TZVPP/SMD level of theory.

**Conformer**	$\delta_{(S)\text{-Mosher}}$ **CH_3 (singlet)**	$\sigma_{(S)\text{-Mosher}}$ **CF_3 (singlet)**
CONF1	3.495	259.495

4.4.10 Refined Ensemble of the (*R*,*S*)-Mosher's Acid Complex with the *Closed* MAC

Table S56: Calculated ^{1}H-NMR chemical shifts (δ, ppm) and ^{19}F-NMR chemical shielding (σ, ppm) for the investigated nuclei of Mosher's Acid in each homochiral complex conformation. All geometries originate from the CREST conformational sampling and CENSO refinement. Chemical shifts were computed at the ωB97X–V/def2-TZVPP/SMD level of theory.

**Conformer**	$\delta_{(R)\text{-Mosher}}$ **CH_3 (singlet)**	$\sigma_{(R)\text{-Mosher}}$ **CF_3 (singlet)**
CONF1	3.990	259.347

Table S57: Calculated ^{1}H-NMR chemical shifts (δ, ppm) and ^{19}F-NMR chemical shielding (σ, ppm) for the investigated nuclei of Mosher's Acid in each heterochiral complex conformation. All geometries originate from the CREST conformational sampling and CENSO refinement. Chemical shifts were computed at the ωB97X–V/def2-TZVPP/SMD level of theory.

**Conformer**	$\delta_{(S)\text{-Mosher}}$ **CH_3 (singlet)**	$\sigma_{(S)\text{-Mosher}}$ **CF_3 (singlet)**
CONF1	3.889	260.359
CONF2	3.531	258.249
CONF3	3.552	257.950
**Boltzmann Avg.**	**3.697**	**259.134**

4.5 Interatomic Distances for Selected Hydrogen Atoms of the MAC

Table S58: Calculated distances (in Å) between the hydrogen H_e and the hydrogens: H_a, H_b, H_c, and H_d of the free chiral macrocycle for both *open* and *closed* conformers.

**MAC Conformation**	**H_e—H_a**	**H_e—H_b**	**H_e—H_c**	**H_e—H_d**
Open	6.919	3.093	8.147	11.081
closed	4.284	3.027	6.530	5.741

Table S59: Calculated distances (in Å) between the hydrogen H_e and the hydrogens: H_a, H_b, H_c, and H_d of the chiral macrocycle in heterochiral and homochiral complexes with mandelic acid, for both *open* and *closed* conformers.

**MAC Conformer**	**Complex Type**	**H_e—H_a**	**H_e—H_b**	**H_e—H_c**	**H_e—H_d**
Open	Homochiral	7.213	3.146	8.416	11.351
	Heterochiral	7.422	3.319	8.964	11.392
closed	Homochiral	7.661	3.228	6.463	8.631
	Heterochiral	6.606	3.308	7.768	8.187

4.6 Gibbs Free Energy of the Free *Open* and *Closed* MAC

Table S60: Gibbs free energies (ΔG, in Hartree) of the isolated *open* and *closed* MAC forms computed at the GFN2-xTB (CREST) and ωB97X–V/def2-TZVPP/SMD (CENSO) levels of theory. The relative Gibbs free energy differences ($\Delta\Delta G_{\text{closed}-\text{open}}$, in kcal mol^{-1}) are also reported.

**MAC Conformation**	ΔG **at GFN2-xTB/ALPB**	ΔG **at** ω**B97X–V/def2-TZVPP/SMD**
Open	-157.2558951	-2602.7862847
Closed	-157.2622558	-2602.7861368
$\Delta\Delta G_{\text{closed}-\text{open}}$	**-3.991**	**0.093**

References

[1] Pelta, M. D.; Morris, G. A.; Stchedroff, M. J.; Hammond, S. J. A one-shot sequence for high-resolution diffusion-ordered spectroscopy. *Magnetic Resonance in Chemistry* **2002**, *40*, S147–S152.

[2] Dal Poggetto, G.; Favaro, D. C.; Nilsson, M.; Morris, G. A.; Tormena, C. F. 19F DOSY NMR analysis for spin systems with nJFF couplings. *Magnetic Resonance in Chemistry* **2014**, *52*, 172–177.

[3] Castañar, L.; Poggetto, G. D.; Colbourne, A. A.; Morris, G. A.; Nilsson, M. The GNAT: A new tool for processing NMR data. *Magnetic Resonance in Chemistry* **2018**, *56*, 546–558.

[4] Cabral, T. L. G.; Dal Poggetto, G.; Tormena, C. F.; Nilsson, M. Practical Guide and Best Practices for Diffusion NMR Processing With GNAT. *Magnetic Resonance in Chemistry* **2026**, 1–16.

[5] Hanwell, M. D.; Curtis, D. E.; Lonie, D. C.; Vandermeersch, T.; Zurek, E.; Hutchison, G. R. Avogadro: an advanced semantic chemical editor, visualization, and analysis platform. *Journal of Cheminformatics* **2012**, *4*, 17.

[6] Bannwarth, C.; Caldeweyher, E.; Ehlert, S.; Hansen, A.; Pracht, P.; Seibert, J.; Spicher, S.; Grimme, S. Extended tight-binding quantum chemistry methods. *Wiley Interdisciplinary Reviews: Computational Molecular Science* **2021**, *11*, e1493.

[7] Bannwarth, C.; Ehlert, S.; Grimme, S. GFN2-xTB—An accurate and broadly parametrized self-consistent tight-binding quantum chemical method with multipole electrostatics and density-dependent dispersion contributions. *Journal of Chemical Theory and Computation* **2019**, *15*, 1652–1671.

[8] Plett, C.; Grimme, S. Automated and efficient generation of general molecular aggregate structures. *Angewandte Chemie International Edition* **2023**, *62*, e202214477.

[9] Ehlert, S.; Stahn, M.; Spicher, S.; Grimme, S. Robust and efficient implicit solvation model for fast semiempirical methods. *Journal of Chemical Theory and Computation* **2021**, *17*, 4250–4261.

[10] Pracht, P.; Bohle, F.; Grimme, S. Automated exploration of the low-energy chemical space with fast quantum chemical methods. *Physical Chemistry Chemical Physics* **2020**, *22*, 7169–7192.

[11] Pracht, P.; Grimme, S.; Bannwarth, C.; Bohle, F.; Ehlert, S.; Feldmann, G.; Gorges, J.; Müller, M.; Neudecker, T.; Plett, C.; Spicher, S.; Steinbach, P.; Wesołowski, P. A.; Zeller, F. CREST—A program for the exploration of low-energy molecular chemical space. *The Journal of Chemical Physics* **2024**, *160*, 114110.

[12] Cabral, T. L. G.; Brussolo da Silva, J. P.; Tormena, C. F.; Stein, M. Molecular Recognition and Chiral Discrimination from NMR and Multi-Scale Simulations. *Chemistry–A European Journal* **2025**, *31*, e202404694.

[13] Spicher, S.; Grimme, S. Single-point Hessian calculations for improved vibrational frequencies and rigid-rotor-harmonic-oscillator thermodynamics. *Journal of Chemical Theory and Computation* **2021**, *17*, 1701–1714.

[14] Grimme, S.; Bohle, F.; Hansen, A.; Pracht, P.; Spicher, S.; Stahn, M. Efficient quantum chemical calculation of structure ensembles and free energies for nonrigid molecules. *The Journal of Physical Chemistry A* **2021**, *125*, 4039–4054.

[15] Neese, F.; Wennmohs, F.; Becker, U.; Riplinger, C. The ORCA quantum chemistry program package. *The Journal of Chemical Physics* **2020**, *152*, 224108.

[16] Marenich, A. V.; Cramer, C.; Truhlar, D. Universal solvation model based on solute electron density and a continuum model of the solvent defind by the bulk dielectric constant and atomic surface tensions. *The Journal of Physical Chemistry B* **2009**, *113*, 6378–6396.

[17] Cabral, T. L. G.; Dal Poggetto, G.; Brussolo da Silva, J. P.; Nilsson, M.; Tormena, C. F. Determining the absolute configuration of small molecules by diffusion NMR experiments. *Angewandte Chemie International Edition* **2025**, *137*, e202418508.
